# The genome and life-stage specific transcriptomes of *Globodera pallida* elucidate key aspects of plant parasitism by a cyst nematode

**DOI:** 10.1186/gb-2014-15-3-r43

**Published:** 2014-03-03

**Authors:** James A Cotton, Catherine J Lilley, Laura M Jones, Taisei Kikuchi, Adam J Reid, Peter Thorpe, Isheng J Tsai, Helen Beasley, Vivian Blok, Peter J A Cock, Sebastian Eves-van den Akker, Nancy Holroyd, Martin Hunt, Sophie Mantelin, Hardeep Naghra, Arnab Pain, Juan E Palomares-Rius, Magdalena Zarowiecki, Matthew Berriman, John T Jones, Peter E Urwin

**Affiliations:** 1 Wellcome Trust Sanger Institute, Wellcome Trust Genome Campus, Cambridge CB10 1SA, UK; 2 Centre for Plant Sciences, University of Leeds, Leeds LS2 9JT, UK; 3 The James Hutton Institute, Invergowrie, Dundee DD2 5DA, UK; 4 Forestry and Forest Products Research Institute, Tsukuba, Japan; 5 Division of Parasitology, Department of Infectious Disease, Faculty of Medicine, University of Miyazaki, Miyazaki 889-1692, Japan; 6 Biodiversity Research Center, Academia Sinica, Taipei 11529, Taiwan; 7 Present address: School of Life Sciences, Centre for Biomolecular Sciences, University of Nottingham, Nottingham NG7 2RD, UK; 8 Present address: Computational Bioscience Research Center (CBRC), Biological and Environmental Sciences and Engineering (BESE) Division, King Abdullah University of Science and Technology, Thuwal 23955-6900, Kingdom of Saudi Arabia; 9 Present address: Institute for Sustainable Agriculture (IAS), Spanish National Research Council (CSIC), Alameda del Obispo s/n Apdo 4084, 14080 Córdoba, Spain

## Abstract

**Background:**

*Globodera pallida* is a devastating pathogen of potato crops, making it one of the most economically important plant parasitic nematodes. It is also an important model for the biology of cyst nematodes. Cyst nematodes and root-knot nematodes are the two most important plant parasitic nematode groups and together represent a global threat to food security.

**Results:**

We present the complete genome sequence of *G. pallida*, together with transcriptomic data from most of the nematode life cycle, particularly focusing on the life cycle stages involved in root invasion and establishment of the biotrophic feeding site. Despite the relatively close phylogenetic relationship with root-knot nematodes, we describe a very different gene family content between the two groups and in particular extensive differences in the repertoire of effectors, including an enormous expansion of the SPRY domain protein family in *G. pallida*, which includes the SPRYSEC family of effectors. This highlights the distinct biology of cyst nematodes compared to the root-knot nematodes that were, until now, the only sedentary plant parasitic nematodes for which genome information was available. We also present in-depth descriptions of the repertoires of other genes likely to be important in understanding the unique biology of cyst nematodes and of potential drug targets and other targets for their control.

**Conclusions:**

The data and analyses we present will be central in exploiting post-genomic approaches in the development of much-needed novel strategies for the control of *G. pallida* and related pathogens.

## Background

There are over 4,100 species of plant parasitic nematodes [1] which collectively are an important threat to global food security. Damage caused to crops worldwide by plant parasitic nematodes has been estimated at $80 billion per year [2]. The largest economic losses to agriculture are imposed by root-knot nematodes and cyst nematodes that both belong to the order Tylenchida. The most widespread and damaging species of root-knot nematodes have a wide host range and are prevalent in Mediterranean, subtropical and tropical regions while cyst nematode species have more restricted host ranges and the most damaging species are found predominantly in more temperate agricultural regions. Both root-knot and cyst nematodes are obligate, sedentary endoparasites that have unique, biotrophic interactions with their host plants. A central feature of the parasitism is the establishment and maintenance of a permanent feeding site that sustains the nematode throughout its growth in the plant [3]. However, biotrophic parasitism of plants by root-knot nematodes and cyst nematodes has evolved independently [4] and this is reflected in the different feeding structures of these nematodes.

The most economically important cyst nematode species are within the *Heterodera* and *Globodera* genera. Cyst nematodes cause significant damage to a range of crops worldwide, particularly potato, soybean, wheat and rice. Potato cyst nematode (PCN) is the collective term for the two species *G. pallida* and *G. rostochiensis* that are restricted to infecting a few species of Solanaceous plants. PCN is a major pest of the potato crop in cool-temperate areas of the world. Yield losses of potato in excess of 50% due to PCN are reported in the literature (for example, [5]). Although PCN is indigenous to South America, it was introduced into Europe in the 19th century with potato material used for resistance breeding against late blight [6] and is now widely distributed in Europe [7]. From here, PCN has spread to all major potato growing areas of the world including Ukraine and, more recently, Idaho in the USA [8,9]. Integrated pest management of *G. pallida* is based on partially resistant cultivars, crop rotation and nematicides. Resistance against the pathotypes of *G. rostochiensis* predominant in Europe is provided by the H1 gene, which is now available in many potato cultivars, for example, ‘Maris Piper’. However, the lack of a comparable single, dominant natural resistance gene for *G. pallida* has resulted in an emphasis on multi-trait quantitative resistance that is difficult to breed and is more readily overcome by virulent pathotypes. Repeated use of cultivars resistant to *G. rostochiensis* has selected for *G. pallida* in mixed populations [10]. The slow decline rate of the dormant soil population of *G. pallida* makes crop rotation an extremely inefficient management practice [11,12]. Nematicides are thus currently essential to control *G. pallida* and allow favoured, susceptible potato cultivars to be grown at an economically viable cropping frequency. Recent legislation, however, has withdrawn or severely limited their use [13]. Consequently there is an urgent need to develop novel approaches for control of this and other cyst nematodes. Research in this direction will be significantly enhanced by a greater understanding of the molecular basis of the parasitic interaction and the key nematode genes required for this.

Cyst nematodes hatch as second stage juveniles (J2) from eggs contained within cysts in the soil. This process is usually initiated in response to chemicals released from roots of a potential host plant. Upon locating host roots they use their stylet to disrupt the plant tissue and migrate intracellularly through cortical cells towards the vascular cylinder where an initial feeding cell is selected. The nematode secretes proteins from pharyngeal gland cells through the bore of the stylet into the initial feeding cell thus inducing the formation of a syncytial feeding site. Localised cell wall dissolution and protoplast fusion cause the syncytium to progressively enlarge until it eventually incorporates up to 200 neighbouring cells [14]. The syncytium develops wall ingrowths to facilitate water and nutrient uptake from the xylem and acts as a strong nutrient sink, with phloem solutes transported at first apoplasmically and later via plasmodesmata. The syncytium is continually stimulated by stylet secretions and provides the growing cyst nematode with all the nutrients required for development into an adult male or an egg-laying female, a process that takes 3 to 6 weeks. Sex is determined by the size of the syncytium that is induced and whether it gains access to vascular tissues in order to supply plentiful nutrients (reviewed by [15]). The cuticle of the mature female, harbouring eggs containing quiescent J2s within her body, is tanned by a polyphenol oxidase to form the tough cyst that protects the eggs. The cyst becomes detached from the root following death of the plant and the eggs within can remain viable for many years.

Nematodes have been a focus of genomic projects since the 1990s when the free-living bacteriovore *Caenorhabditis elegans* became the first multicellular organism to have a completely sequenced genome [16]. This provided a valuable platform for genomics research in other nematode species, but it was a further decade before the first genome sequence became available for a parasitic nematode, the human filarial parasite *Brugia malayi*[17]. Genome sequences have subsequently been reported for a range of other nematode species [18-20], but only three plant parasitic nematodes: two root-knot nematode species [21] (*Meloidogyne incognita*[22] and *M. hapla*[23]) and most recently the pine wood nematode *Bursaphelenchus xylophilus*, a migratory endoparasite [24]. The draft genome sequence of *G. pallida* reported here is, to our knowledge, the first cyst nematode genome to be described and will serve as a valuable comparator for understanding the evolution of plant parasitism in nematodes. We describe the genome in detail, examining the gene content of *G. pallida* in the context of other published plant parasitic nematode genomes. Significantly, we use RNA-seq to examine changes in gene expression throughout the lifecycle of *G. pallida*, which provides important insights into the genes involved particularly in root invasion and establishment of the feeding site.

## Results and discussion

### General overview of the *G. pallida* genome

The genome of *G. pallida* was sequenced using a mixture of sequencing technologies (see Additional file 1: Table S1 for details), with reads from each technology assembled independently before merging, scaffolding and automated improvement (see Materials and methods, Additional file 1: Figure S1, Table S1 for details). This process produced a draft genome assembly of 124.7 Mb in 6,873 scaffolds of at least 500 bp, with an N50 scaffold length of 122 kbp (Table 1, Additional file 1: Table S2), and with a GC content of 36.7% (Additional file 1: Figure S2). *G. pallida* is highly polymorphic [25], with at least 1.2% of sites being polymorphic in our experimental population alone, and its small size meant multiple individuals were pooled to generate sequencing libraries. The sequencing and assembly of highly polymorphic genomes remains challenging with current sequencing technology, even with a large amount of data from three complementary platforms. Current and future developments in both technology [26] and molecular biology techniques, such as methods for directly sequencing haplotypes [27] may perhaps facilitate the genome analysis of organisms such as *G. pallida*.

**Table 1:** **Comparison of the *Globodera pallida *** **genome with selected other published nematode genomes**

	**Clade IV**				**Clade V**	**Clade III**
	***Globodera pallida***	***Bursaphelenchus xylophilus***	***Meloidogyne hapla***	***Meloidogyne incognita***	***C. elegans***	***Brugia malayi***
**Estimated genome size (Mb)**	100	63–75	54	47–51	100	90–95
**Haploid chromosome number**	9	6	16	Varies	6	6
**Assembly length (Mb)**	124.7	74.6	53	86	100	95.8
**Scaffolds ( *n *)**	6,873	1,231	1,523	2,817	7	8,180
**Scaffold N50 (kb)**	122	1,158	84	83	17,493	94
**Longest scaffold (kb)**	600	3,612	360	593	20,924	6,534
**GC content**	36.7	40.4	27.4	31.4	35.4	30.5
**Gene models ( *n *)**	16,419	18,074	14,420	19,212	20,056	18,348
**Mean protein length (aa)**	361	345	310	354	440	312
**CEGMA completeness**	81/85	97/98	95/96	73/77	100/100	95/96
**(% complete/partial)**						

See Additional file 1: Table S2 for a more complete and broader comparison.

**Figure 1:**
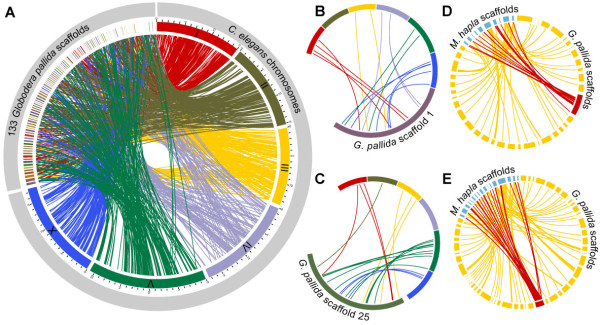
**Scaffolds of *Globodera pallida *show little or no synteny with other nematodes. (A)** Shows all 133 *G. pallida* scaffolds that contain at least five one-to-one orthologs with *Caenorhabditis elegans* with scaffolds ordered to maximise colinearity with the *C. elegans* genome. Lines connect orthologs, and *G. pallida* scaffolds are coloured with a mixture of the colours used for *C. elegans* scaffolds they have orthologs with, weighted by the numbers of orthologs to each. The relative positions of one-to-one orthologs between **(B)** the largest *G. pallida* scaffold (scaffold 1) and **(C)** the *G. pallida* scaffold with the largest number of one-to-one orthologs to *C. elegans* (scaffold 25). Colour and orientation of scaffolds and chromosomes are as in **(A)**. Note that the *G. pallida* and *C. elegans* sequences are not drawn to scale in **(B)** or **(C)**. **(D, E)** Show one-to-one orthologs between *M. hapla* and *G. pallida*, including those *M. hapla* scaffolds (blue) that have orthologs to **(D)***G. pallida* scaffold 1 and **(E)***G. pallida* scaffold 25 (red) and orthologs from those scaffolds to other *G. pallida* scaffolds (yellow).

Comparison of the longest scaffolds from this assembly with the *C. elegans* genome shows no evidence of large-scale synteny or of significant conservation of gene order between the genomes. All of the 133 *G. pallida* scaffolds with at least five one-to-one orthologs to *C. elegans* have orthologs on more than one *C. elegans* chromosome (Figure 1A-C). This is in marked contrast to other nematode species at a similar or even greater phylogenetic distance from *C. elegans* such as the filarial nematodes *B. malayi*[17] and *Loa loa*[20], the plant parasitic nematode *B. xylophilus*[24] or even the very divergent *Trichinella spiralis*[28]. There is limited conservation of synteny between *G. pallida* and *M. hapla* - of 216 *G. pallida* scaffolds with at least five one-to-one orthologs to *M. hapla*, six have orthologs to a single *M. hapla* scaffold, despite the draft nature of both assemblies, and some conservation of gene order within scaffolds is observed (Figure 1D, E). There is wider variation in karyotype within clade IV, of which *G. pallida* is a member, than other nematode clades, with haploid chromosome number varying within genera [29-32] and even within species [33] in this group but being stable at *n* = 6 for all members of clade V [34]. The recombination rate in *M. hapla* is more than 50-fold higher than the estimated rate for *C. elegans*[35]. Together, these data suggest that there has been a high rate of large-scale genome rearrangement in the evolutionary history of the lineage leading to *G. pallida* and other Tylenchids and, in particular, present the possibility that inter-chromosomal rearrangements may be more common in clade IV than elsewhere in the phylum. Confirmation of this will require higher-quality reference genomes for multiple members of this clade.

Although the *G. pallida* genome is fragmented, it still appears to be fairly complete, as approximately 85% of conserved eukaryotic genes can be identified in our assembly (Additional file 1: Table S2), and 81% of EST clusters map to the genome suggesting that at least that proportion of *G. pallida* genes are represented. The assembly is approximately 17% repetitive, with only around 1.8% showing similarity to transposable elements (Additional file 1: Table S3). No intact transposable elements were identified in the genome, confirming that most, or all, transposable elements are inactive. The longest LTR consensus is 5.3 kb long and the closest match is the Pao retrotransposon peptidase family protein from *B. malayi*.

### The protein-coding repertoire of *G. pallida*

Using a combination of manual curation and transcriptomic evidence (see Materials and methods and Additional file 1: Table S4) a total of 16,419 genes were predicted in the *G. pallida* genome, intermediate between the gene counts reported for the two *Meloidogyne* genomes currently available. RNA-seq evidence from the extensive transcriptomic dataset we have generated (see below) supports the transcription of a total of 15,329 (93.4%) of the predicted gene models. At least one predicted protein domain or other InterPro feature was predicted for 14,139 of the gene models and 8,700 genes could be annotated with at least one Gene Ontology term.

A compact genome with high gene density may be characteristic of obligate parasitic lineages (for example, [36]). This is clearly the case for some plant parasitic nematodes; the *M. hapla* genome is the smallest published animal genome [21] and the tylenchid *Pratylenchus coffeae* is estimated to have the smallest genome of any animal [37,38], but *G. pallida* does not follow this pattern. The significantly lower gene density of the *G. pallida* genome compared to other plant parasitic nematodes cannot be attributed to any single factor: on average, *G. pallida* has rather longer gene models than either *Meloidogyne* species, with more exons per gene and slightly longer introns (Additional file 1: Table S2), but both gene number and the proportion of the genome that is repetitive (12% in *M. hapla*, 36% in *M. incognita*, 22% in *B. xylophilus*) are similar to those for the other published species, suggesting that a greater proportion of the *G. pallida* genome is non-repetitive, non-coding DNA.

Two different approaches were used to compare the *G. pallida* proteome with those of other nematodes (see Materials and methods). We found 6,714 gene families that contain at least one *G. pallida* protein, with 3,890 *G. pallida* genes not clustered into any family and 825 gene families unique to *G. pallida*. Functional analysis of both of these sets of *G. pallida*-restricted proteins using annotated GO terms (Additional file 1: Table S5) suggests that they are significantly enriched in membrane and extracellular proteins and proteins involved in carbohydrate and protein catalysis, which might play a role in the host-parasite interaction. Furthermore, there is enrichment of proteins potentially involved in activities related to mediating the complex life-cycle such as neurogenesis and neurotransmission, cuticle development and defence responses. The set of unique genes in *G. pallida* is also predictably enriched for proteins with little or no functional annotation, highlighting the need for further functional characterisation of *G. pallida* proteins. Among the largest gene families in the *G. pallida* genome are the SPRY domain proteins which include the SPRYSECs (secreted proteins containing a SPRY domain) and a family of proteins similar to the *Heterodera glycines* (soybean cyst nematode) effectors 4D06 and G16B09 (see below). In addition, a family of 474 *G. pallida* genes show similarity to a gene annotated as ‘dorsal gland cell-specific expression protein’ from the cereal cyst nematode *Heterodera avenae* (Genbank HM147943.1). These proteins are highly divergent and the consensus sequence has no homolog in *C. elegans.* The absence of functional data for any of these ‘dorsal gland’ proteins makes it difficult to analyse the significance of the expansion in *G. pallida*. However, RNA-seq data show that some of the gene copies are highly expressed exclusively in the male samples. Some members of this gene family clearly have a different function in *G. pallida* compared to *H. avenae; in situ* hybridisation analysis of a small number of the *G. pallida* genes has shown that some are expressed in the digestive system (Additional file 1: Figure S3) with none of the sequences tested to date showing expression in the gland cells. However, the sequences chosen for analysis were selected on the basis of expression at the early stages of parasitism, rather than by similarity to the *H. avenae* sequence. Another expanded gene family, encoding glutathione synthetase proteins, is discussed in detail below.

**Figure 2:**
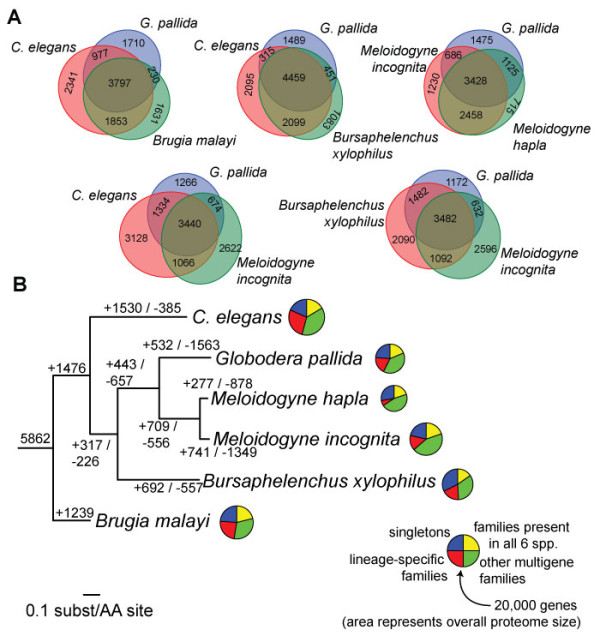
**Comparative genomics of *Globodera pallida *and other plant parasitic nematodes. (A)** Euler diagrams of shared presence-and-absence of gene families in plant-parasitic nematodes with published genome descriptions, the free-living model *Caenorhabditis elegans* and the spirurid animal parasite *Brugia malayi*. **(B)** Phylogenetic analysis of genome content. Tree shown is a maximum-likelihood phylogeny based on concatenated alignment of single-copy orthologs. Values on edges represent the inferred numbers of births (+) and deaths (-) of gene families along that edge. Note that our approach cannot distinguish gene family losses from gains on the basal branches of this tree, so for example the value of 1,476 gene family gains on the basal branch will include gene families lost on the branch leading to *B. malayi*. Pie charts represent the gene family composition of each genome - the area of the circle is proportional to the predicted proteome size, and wedges represent the numbers of proteins predicted to be either singletons (that is, not members of any gene family), members of gene families common to all six genomes, members of gene families present only in a single genome, and members of all other gene families.

Extensive genetic and genomic resources and a powerful molecular genetic toolkit make the free-living nematode *C. elegans* an important model system for studying a range of aspects of plant parasitic nematode biology [39,40]. Supporting this, the majority of *G. pallida* gene families contain *C. elegans* homologs (4,774 or 71%), although only 2,044 *G. pallida* genes have a one-one ortholog in *C. elegans*. However, many aspects of plant parasitic nematode biology cannot be studied in a free-living system. This is reflected in the substantial genetic repertoire that *G. pallida* shares with related nematodes but that is not found in *C. elegans*: 331 gene families are uniquely found in the three tylenchid species (*G. pallida* and two *Meloidogyne* spp.) and another 121 families are found in *B. xylophilus* and tylenchids (Figure 2A). While 2,976 genes have one-one orthologs between *G. pallida* and *M. hapla*, we find substantial variation in gene content between *G. pallida* and the root-knot nematodes - in total, *G. pallida* shares 741 gene families with other nematodes that are not present in either species of *Meloidogyne*. Indeed, *G. pallida* shares fewer gene families with *M. incognita* or *M. hapla* than with *B. xylophilus* (but more one-one orthologs with *M. hapla*), despite *Meloidogyne* and *Globodera* being more closely related. Phylogenetic reconstruction of the pattern of gene duplication and loss in the genomes of plant parasitic nematodes (Figure 2B) suggests this pattern is largely driven by differential gene loss between the cyst nematode and root-knot nematode lineages, although these figures could be somewhat inflated by the incompleteness of these draft genomes. Our findings confirm that the different molecular mechanisms of parasitism exploited by cyst and root-knot nematodes are reflected in a different complement of genes, particularly with respect to the repertoire of effector genes specifically involved in establishing and maintaining the host-parasite interface (see below), reflecting the independent origins of biotrophic parasitism in the two groups.

Organisation of genes into co-located and co-transcribed operons is a major feature of nematode genomes, with approximately 17% of *C. elegans* genes organised in operons [41]. Only 7% of *C. elegans* operons appear to be conserved in *G. pallida*, but transcriptomic evidence suggests that *G. pallida* genes are arranged in operons (see Supplementary Results and Additional file 1: Figure S4). In *C. elegans*, polycistronic pre-mRNAs transcribed from operons are processed to form the mature mRNA by trans-splicing with spliced leader (SL) sequences. SL1 is trans-spliced to the first gene in an operon, while downstream genes are trans-spliced with SL2 [41]. Our RNA-seq data confirm that a diverse range of different SL types previously reported in *G. rostochiensis*[42] are also found trans-spliced to *G. pallida* transcripts. SL1-type sequences are found predominantly, but most genes appear to be promiscuously spliced to any of the SLs. In contrast to the situation in *C. elegans*[41], there is little evidence of a strong correlation in SL usage with distance between adjacent genes or expression pattern. The functional relevance of the diverse SL sequences in *G. pallida* is thus unclear.

### Transcriptome and differential gene expression in the *G. pallida* life cycle

**Figure 3:**
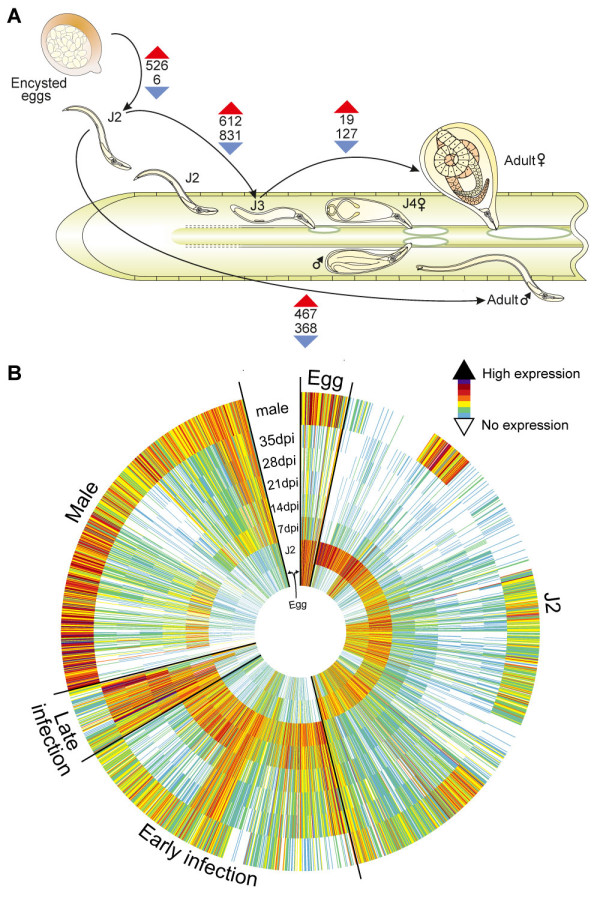
**Transcriptional profiling of the *G. pallida *lifecycle. (A)** Number of genes up- and downregulated between different stages in the *G. pallida* lifecycle. Labelled transitions are between egg and J2, J2 and early infection (7 and 14 dpi), early and late infection (21, 28 and 35 dpi) and J2 larvae and adult males. **(B)** Heatmap showing clustered expression profiles for all 2,052 differentially expressed genes. Genes are clustered to reflect similarity of expression profiles and then ordered by stage of highest expression, as labelled on the circumference of the figure.

The relative expression of all *G. pallida* genes was determined by replicated Illumina RNA-seq across eight life stages. We examined unhatched J2 larvae within eggs, hatched invasive stage J2, adult males and parasitic individuals at early (7 and 14 days post infection (dpi)) and late (21, 28 and 35 dpi) stages post-infection of potato roots (Additional file 1: Table S4). The results reveal the dynamics of transcription across the *G. pallida* life cycle (Figure 3) with only 2,052 genes showing highly significant (FDR <10^−5^) changes in expression between different life stages (see Additional file 2 for full lists of differentially expressed genes). Many of these differentially expressed genes encode hypothetical proteins (1,417 - 57%), a significantly greater proportion than for non-differentially expressed genes. The number of genes expressed in each life stage varies (Additional file 1: Figure S5) with J2 larvae and adult males, the motile stages, showing high numbers of expressed genes. The number of genes expressed generally declines as the nematodes develop, with particularly low levels of gene activation during the development of adult females. A modest increase in the latest adult female stage presumably correlates with the development of embryos within the female. Transcript diversity follows a similar trend, except that the adult male transcriptome lacks diversity. It is dominated by a relatively small number of highly expressed transcripts, of which the major sperm protein has 10-fold higher expression than any other transcript. Other highly expressed transcripts in male nematodes are two of unknown function, a creatine kinase and one of the large ‘dorsal gland cell specific’ gene family discussed above. The transcriptome of adult females at 35 dpi is notably more diverse than expected from the low absolute number of different transcripts present.

Following stimulation of hatching in response to host root exudates, motile infective J2 larvae emerge from eggs within cysts, locate and then penetrate the potato root. A large-scale activation of transcription accompanies the hatching of J2s. Among the most enriched functional classes in this stage are 11 genes with poly-A transferase activity, most of which show similarity to poly-A polymerase gamma genes from other species that add poly-A to pre-mRNAs. This may reflect the need for large scale upregulation of transcription as the nematode emerges from dormancy. Carbohydrate metabolism is also upregulated in the transition to the hatched J2, including six cellulase genes and three pectate lyase genes presumably involved in host invasion and a chitinase that could be involved in the hatching process. The pre-parasitic J2 is protected neither by the eggshell nor within the host and is exposed both to pathogens and to plant defence molecules during initial root invasion. Correspondingly, a number of genes involved in defence responses are upregulated in this stage. In addition, genes upregulated in J2 are enriched for products that localise outside the cell, in the lysosome and the ER, possibly reflecting the secretion of proteins that mediate interactions with the host (effectors; see below).

After the syncytium is induced and feeding commences, the J2 nematode undergoes three moults to reach the adult stage. At 7 dpi both late parasitic J2 stage and early J3 larvae were present, whilst nematodes collected at 14 dpi were J4 females. The transition from infective J2 to these early parasitic stages is accompanied by the largest changes in gene expression during the lifecycle (Figure 3A). The clearest group of upregulated genes is a large set of glutathione synthetase genes - these are discussed in more detail later. Other changes include the upregulation of many genes involved in lipid metabolism and proteolysis, in particular astacin proteases, and some cuticle collagens. These changes likely reflect the start of feeding by the post-parasitic J2 and moulting to the J3 and J4 female. Most downregulated gene classes appear to correlate well with transition from a motile free-living organism to sedentary parasitism. Expression of genes involved in signal transduction such as G protein-coupled receptors (GPCRs), GPCR signalling through cyclic nucleotides, sodium and potassium ion transport, neurotransmitter metabolism and oxygen transport is reduced. This interpretation is re-enforced by the downregulation of homologs of a number of genes with well-understood functions in neurotransmission and chemotaxis in *C. elegans* (*egl-3*, *osm-3* and a EXP-family potassium channel [43-45]).

The female worms are adult by 21 dpi, and their continued development through to 35 dpi is accompanied by enlargement and swelling to a spherical shape. Embryos develop within fertilised females and the J1 larvae undergo the first moult inside the eggs contained within the female body. Despite this development, we find relatively few changes in expression between the early and late parasitic stages. Upregulated genes are enriched for functions in lipid transport and chitin catabolism as lipid stores are provided to the developing embryos and chitin is laid down in egg shells. The most highly expressed genes in both 28 and 35 dpi samples encode vitellogenin and a number of cuticle collagens. These reflect the accumulation of yolk proteins within oocytes and the subsequent synthesis of cuticular material for the J1 and J2 nematodes that develop within the eggs.

The sexual fate of cyst nematodes first becomes apparent at the end of the parasitic J2 stage, shortly before the moult to J3. Males feed until the end of the J3 stage before a motile, vermiform adult male develops within the J4 cuticle, then emerges and leaves the root to locate and fertilise females. Although some genes associated with motility are shared between males and pre-parasitic J2s, the transcriptome of males is very distinct from both the early parasitic stages and the J2. Eight α,α-trehalase genes, which encode the enzyme responsible for hydrolysing trehalose to produce glucose, are upregulated in males. While these could be involved in mobilising stored trehalose for energy in the motile stage, it is not clear why this should differ between J2 and adult males. However, trehalose plays a number of different roles in nematodes and is particularly enriched in reproductive tissues [46]. Upregulation in males of genes involved in proteolysis, ubiquitination and other aspects of protein metabolism such as glycosylation and phosphorylation might reflect the protein turnover that presumably accompanies a change back to a free-living lifestyle. Changes in lipid metabolism genes were also consistent with this; the adult male does not feed and relies on the mobilisation of stored lipid. A number of proteins that localise to nucleosomes were significantly enriched, perhaps suggesting some chromatin remodelling or cell divisions associated with production of sperm. Several expression changes, such as a homolog of a testis specific protein kinase and major sperm protein (MSP) are clear markers for male reproductive machinery - indeed, the latter is the most highly expressed gene in the male samples.

Complementing the pairwise comparisons between lifecycle stages, clustering of gene expression profiles clearly demonstrated that changes in the transcript profiles accurately reflect changes in *G. pallida* biology across the life cycle. For example, the J2 and adult male are the only mobile stages of the nematode. A cluster of 154 genes was identified as being specifically upregulated in both of these life stages; analysis of gene ontology terms significantly enriched in this cluster showed that all were related to neuromuscular function (Additional file 1: Figure S6A). Similarly, a cluster of 59 genes upregulated in parasitic stages was significantly enriched for gene ontology terms relating to cuticle synthesis and protein digestion, reflecting the fact that these life stages are actively feeding and undergoing repeated moults (Additional file 1: Figure S6B).

### Genomic insights into the mechanisms of plant parasitism in *Globodera*

*G. pallida* is a complex, biotrophic pathogen that has intimate interactions with its host. These interactions are mediated by effector proteins (also termed parasitism proteins) responsible for a variety of processes: modification of the host cell wall during invasion, induction of the feeding structure, manipulation of host metabolism for the nutritional benefit of the nematode and suppression of host defence responses to ensure maintenance of the feeding site. Effectors have previously been identified from plant parasitic nematodes through EST sequencing (for example, [47]), expression profiling [48] and through sequencing of mRNA extracted from aspirated gland cell cytoplasm [49], each followed by *in situ* hybridisation to confirm gland cell expression of the candidate genes. Analysis of the *G. pallida* genome showed that it contains orthologs of many of the effectors previously identified from other cyst nematodes (Additional file 1: Table S7). However, with the exception of enzymes that degrade the plant cell wall and chorismate mutases (see below), there is almost no overlap between effectors identified from root-knot nematodes and cyst nematodes. These findings are consistent with the idea that biotrophic interactions with plants have arisen independently in root-knot and cyst nematodes (for example, [50]). Just two candidate effector types from *G. pallida* (GPLIN_000604400 (similar to GPLIN_000555600) and GPLIN_001475500) have matches in root-knot nematodes and the first of these, similar to *M. incognita* effector accession number AY135365, is also present in (non-biotrophic) migratory endoparasitic nematodes (for example, *P. coffeae* - A. Haegeman, pers comm) but is not present in non-plant parasitic species. This effector may have a conserved role in the infection of plants by nematodes.

Plant parasitic nematodes are known to possess a variety of plant cell wall modifying enzymes, many of which have been acquired by horizontal gene transfer from bacteria (reviewed by [51]). *G. pallida* has a complex array of cell wall modifying enzymes (Additional file 1: Table S8) with a broadly similar repertoire of enzymes to that described for *M. incognita*[22], except that *G. pallida* lacks GH28 polygalacturonases, and the GH53 arabinogalactan endo-1,4 beta galactosidases may be specific to cyst nematodes as they are present in *G. pallida* and *Heterodera schachtii*[52] but are absent from *M. incognita* and *M. hapla*. In addition four genes (GPLIN_000483300, GPLIN_000949800, GPLIN_000950300 and GPLIN_001068900) that could encode secreted GH32 fructosidases are present in *G. pallida*. These enzymes could metabolise sucrose into fructose and glucose and are similar to the invertases previously described from *M. incognita* and *M. hapla. Globodera pallida* also contains two putative chorismate mutases that are likely to have been acquired by horizontal gene transfer from bacteria [53]. Similar genes have been described from a range of plant parasitic nematodes. In addition, although they are not effectors, two genes potentially involved in Vitamin B6 biosynthesis are present in cyst nematodes that are likely to have been acquired from bacteria [54]. These two sequences are present in *G. pallida*, are located side by side on the same scaffold (Gpal_scaffold_166) in the assembly and show almost identical expression profiles.

**Figure 4:**
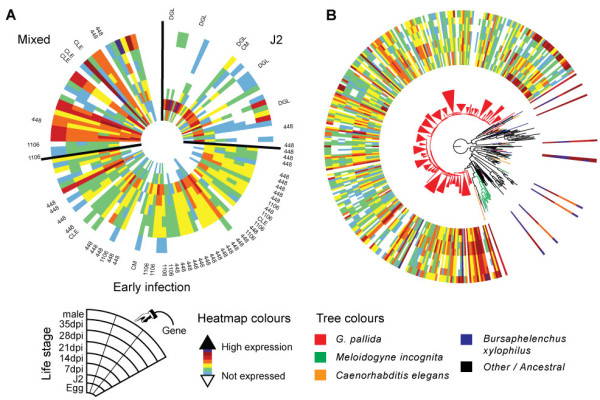
**Expression dynamics of *Globodera pallida *effectors. (A)** Heatmap of 123 effector genes highlights the expression of dorsal-gland like protein (DGL) genes in J2 and 4D06 (448) family effector homologs in early infection. A variety of effector-like genes is also expressed across stages. One chorismate mutase (CM) is expressed in J2, while another is expressed principally in early infection. **(B)** SPRYSEC genes are present in a wide range of nematodes, but are massively expanded in *G. pallida*. The phylogenetic tree shows that some homologs, including the two most highly expressed across stages, are distributed among those from other species. The *G. pallida* radiation is monophyletic however. Most copies are expressed and expression does not often correlate with phylogenetic clusters. Expression tends to be high during the early stage of parasitism, however one particular phylogenetic cluster shows high expression in eggs and males.

Effector proteins are secreted from two sets of gland cells (dorsal and subventral), through the stylet and into the host. These cells show distinct developmental profiles. The subventral glands are large and full of secretory granules in preparasitic and early parasitic J2s, but contain fewer secretory granules during parasitism before becoming active again in adult males. In contrast, the dorsal gland cells are smaller in J2 but increase in size and activity throughout the sedentary parasitic stages [55]. The expression of effectors we have identified reflects this, with particular families showing peak expression in either the J2 or early infection stages (Figure 4A). Effectors identified as being J2-specific included those for which there is experimental verification of subventral gland cell expression in *G. pallida*, such as the chorismate mutases [53]. Several plant cell wall-degrading enzymes were expressed in both J2s and in males, stages that need to enter and escape from the host root, respectively, and reflecting experimentally verified expression profiles (for example, [56]). Two additional effectors of unknown function also shared this expression profile. Many other effectors showed elevated expression in parasitic stages and these included *G. pallida* orthologs of effectors known to be dorsal gland specific in other plant parasitic nematodes (for example, [49,57]).

Some of the *G. pallida* effectors are present in large multigene families. One family of proteins, similar to *H. glycines* effectors 4D06 and G16B09, has approximately 40 members in *G. pallida* (Additional file 1: Table S7). Over 30 of these are significantly upregulated in parasitic stages. However, perhaps the most significant example of an expanded gene family is provided by the SPRY domain proteins, a family that includes the SPRYSECs: a family of known effector proteins in *G. pallida*[47] and *G. rostochiensis*[58] (see Additional file 1: Table S9). One *G. rostochiensis* SPRYSEC (*G. rostochiensis* Sprysec 19) is known to interact with a resistance protein [58] and one *G. pallida* SPRYSEC (RBP1) has been identified as the avirulence factor recognised by the resistance protein Gpa2 [59], suggesting that this gene family may be under strong selection pressure to evade recognition by the host. While all nematodes examined to date have SPRY domain containing proteins, these are typically not secreted and the 299 *G. pallida* proteins predicted to have one or more SPRY domains represent an enormous expansion over that found in other nematodes (for example, *C. elegans*, *B. xylophilus* and *M. incognita* have 8, 12 and 27, respectively). Some of the *G. pallida* SPRY domain proteins are closely related to homologs from *B. xylophilus* and *M. incognita* and are constitutively expressed, but most form part of a large lineage-specific expansion of proteins, with many showing peaks of expression in J2s (Figure 4B). All of the secreted SPRY domain proteins (a minimum of 37 sequences) are included in this expansion.

A bioinformatic approach combining the genome and transcriptome information was also used to identify novel candidate effectors from *G. pallida*. Secreted proteins that are significantly upregulated in J2s (as compared to eggs) or in nematodes at 7 dpi (*versus* J2) were first identified and BLAST was then used to remove proteins that clearly have another functional role (for example, collagens and digestive proteases). The results of this analysis are summarised in Additional file 1: Table S10. A total of 117 proteins were identified that met the criteria and represent potential novel effectors; some of these genes were previously identified as potential novel effectors in an analysis of *G. pallida* ESTs [47].

### Protection against plant defences and other environmental stresses

Some plant defence responses involve production of reactive oxidative radicals [60] and plant-parasitic nematodes are likely to have evolved specialised systems to neutralise these cytotoxic responses. A key step in this process is the generation of hydrogen peroxide, catalysed by superoxide dismutase (SOD) enzymes and the *G. pallida* genome contains an expanded family of 10 SOD genes (Additional file 1: Table S11). These enzymes mostly show homology to *C. elegans* Cu/Zn *sod-1* involved in stress responses [61]. Cyst nematode J2s migrate intracellularly through host roots, causing considerable tissue damage and necrosis, whereas J2s of root-knot nematode migrate intercellularly, eliciting little response from the host. This difference may account for the increased repertoire of *G. pallida* genes involved in neutralisation of the oxidative free radicals produced by the plant. As expected, *G. pallida* also contains sets of genes involved in the rapid breakdown of the cytotoxic hydrogen peroxide released during this process, including catalase, peroxiredoxin and glutathione peroxidase genes.

**Figure 5:**
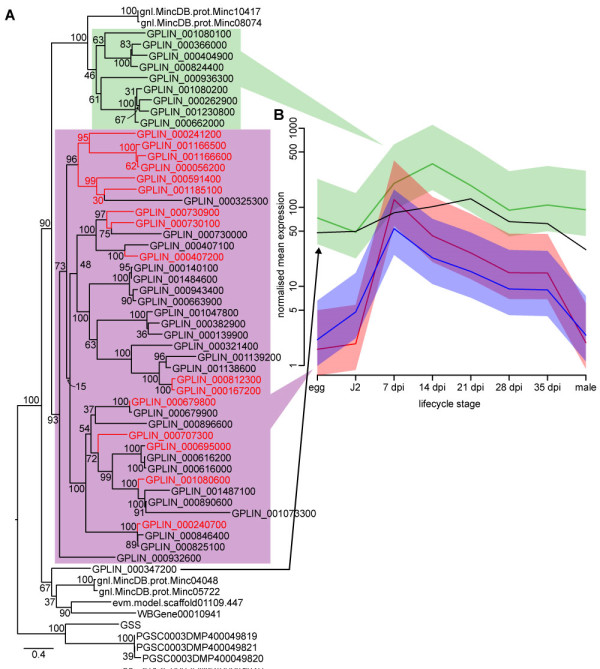
**Phylogeny and expression of glutathione synthetase genes in *G. pallida*. (A)** Phylogeny of all *G. pallida* glutathione synthetase genes that can be aligned over at least 200 bp. Non-*G. pallida* sequences are from *Meloidogyne incognita*, *Bursaphelenchus xylophilus*, *Wuchereria bancrofti* and from mammals. Red sequences are predicted to have signal peptides. **(B)** Different clades of these genes show different expression dynamics. The single-copy outgroup sequence, apparently shared by all nematodes, is constitutively expressed across the lifecycle (black line). Members of the clade shared with some *M. incognita* sequences (green) show a peak of expression at 14 dpi, while the *G. pallida*-specific expansion (purple in panel A) shows a peak of expression at 7 dpi, a pattern more pronounced in copies predicted to have signal peptides (red) than those without (blue). Lines are mean expression across gene copies for each lifecycle stage; shading covers a 99% exponential confidence interval for the mean.

These redox processes all require glutathione and *G. pallida* contains 52 glutathione synthetase genes compared to typically one to four copies in other nematodes. Even more surprisingly, about one-quarter of the genes contain a signal peptide and these all show a peak of expression in the early parasitic stages (7 dpi). Those genes with a predicted cytoplasmic location tend to be expressed more stably throughout the nematode lifecycle (Figure 5). Previous work has shown that, like animal parasites, potato cyst nematodes secrete antioxidant proteins on to their surface including peroxiredoxins [62] and glutathione peroxidase [63] and the expanded repertoire of glutathione synthetase genes in *G. pallida* may produce glutathione to act as co-factors for these proteins. Moreover, glutathione plays a range of functions in plants, including involvement in signalling and regulation of plant development [64], and is essential for reproduction and proper development of nematodes during their infective stage in the host. Depletion of glutathione in host plants reduces the availability of starch and sugars to *M. incognita* during parasitism by this nematode*,* resulting in fewer egg masses and altered sex ratio [65]. While glutathione levels are usually controlled by regulation of γ-glutamylcysteine synthetase, which catalyses the first committed step in glutathione synthesis [66], we propose that *G. pallida* may have evolved to produce high levels of glutathione both internally and within the host cells to stimulate the plant to provide the nematode with adequate carbohydrate nutrition.

Since *G. pallida* feeds only from the host plant, it is unlikely to encounter as wide a range of xenobiotics as free-living nematodes, although its host plants produce a number of toxic tropane alkaloids [67]. This may explain a vast reduction in predicted genes encoding enzymes and transporters involved in costly and specialised cellular metabolism and detoxification of such compounds compared to those found in *C. elegans* (see Additional file 1: Table S11). There are fewer genes involved in all three phases of detoxification of secondary metabolites [68] in *G. pallida*. There is a reduced number and diversity of cytochrome P450 genes (Phase I), fewer glutathione and UDP-glucuronosyl transferases (GSTs and UGTs) (Phase II) and a reduction in ABC transporters (Phase III). The CYP-35 subclass of CYP450 genes (which is particularly associated with xenobiotic metabolism in *C. elegans*) is completely absent in *G. pallida* while the CYP-33 subclass, associated with lipid storage and regulation of endogenous processes [69], is conserved and contains the majority of the *G. pallida* genes. Two CYP-33 genes are highly expressed in J2 compared to parasitic stages and may play a role in lipid regulation in the non-feeding pre-infective stage. Most of the GST genes in *G. pallida* belong to the Sigma class, as found for *C. elegans* and *M. incognita*[70]. The parasitic life-style of *G. pallida* means that it is also likely to directly encounter a reduced array of pathogens compared to the free-living *C. elegans*. We find that most immune signalling pathways appear to be highly conserved between *G. pallida* and other nematodes (Additional file 1: Table S12), with the exception of some members of the Toll pathway which is the pathway responsible for recognising different types of pathogens. In contrast, immune effectors such as lysozymes, C-type lectins and chitinases are much less abundant in *G. pallida* (and *M. incognita*[22]) than in *C. elegans*, and whole classes of antibacterial and antifungal genes, including those encoding antibacterial factors (*abf*), saposin-like proteins (*spp*), fungus-induced proteins (*fip*) and the anti-bacterial neuropeptide-like proteins (*nlp24-33*) are entirely absent.

### Nuclear hormone receptors

Nuclear hormone receptors (NHRs) are a conserved family of ligand-binding transcription factors that regulate diverse physiological processes including metabolism, development, reproduction and immune responses. The receptors bind to an extensive range of lipophilic molecules including fatty acids, vitamins, steroids and xenobiotics, providing a direct link between these ligands and the expression of target genes. They are therefore likely to play a central role in the regulation of lipid metabolism and responses to plant-host defences. The family has undergone a massive expansion to 284 genes in *C. elegans*, the majority of which belong to the group of nematode-specific supplementary NHRs (SupNRs) [71]. *G. pallida* has only 54 NHRs (Additional file 1: Table S13), similar to the predicted repertoire in mammals [72]. Most of the *G. pallida* NHR genes are SupNRs that share little homology between nematode species. The lack of conservation between SupNR members in *G. pallida* and *M. incognita* would suggest that expansion of SupNRs has proceeded independently in the two species. An exception is the homolog to *nhr-88,* which regulates lipid storage in *C. elegans* and is highly expressed in J2s, possibly reflecting the mobilisation of lipid reserves at this stage. One *G. pallida* SupNR conserved only in *C. elegans* (*nhr-25*) is highly expressed during the early stages of infection and may regulate responses to neutralise plant cytotoxic activity.

### Sex determination and diapause

We investigated the conservation in *G. pallida* of two developmental signalling pathways that are well understood in *C. elegans* and underlie key aspects of *G. pallida* biology. Sex determination in *C. elegans* is controlled genetically [73], while in *G. pallida* the sex of each nematode is environmentally influenced, with the food supply determining the sexual fate of developing J2 larvae. Individuals that induce a larger feeding site are more likely to develop into females [15,74-76]. This leads to a greater proportion of males when infection levels are high, and is exploited by plants, as some resistance genes operate by restricting development of the feeding site resulting in fewer of the more damaging females (for example, [15,77]). The *C. elegans* sex determination pathway is only poorly conserved in *G. pallida*, with clear orthologs found only to *C. elegans fem-2*, *mag-1* and *mog-1*, together with *G. pallida* genes showing some similarity to *laf-1*, *gld-1*, *tra-1* and *fem-1. Globodera pallida* is a host-specific pathogen that must coordinate its life cycle with the availability of a suitable host plant. Like many nematodes, including *C. elegans*, *G. pallida* has a survival stage which is adapted for long-term survival in the absence of a food source. The survival stage in *G. pallida* is the unhatched J2, which can survive in cysts for up to 30 years in the absence of a host [78], and is functionally similar to the dauer larva of *C. elegans*[79,80]. However, we find relatively poor conservation of most of the four signalling pathways that control the developmental decision to enter and leave the dauer stage [81] (Additional file 1: Table S14) and not all of the conserved genes show the expected peak of expression either in the egg or the mature female within which the juveniles are developing (Additional file 1: Figure S7). These signalling pathways appear to be a mosaic of conserved genes and genes missing from *G. pallida*, underlining how variable developmental pathways can control development of quite conserved morphology as shown in other nematodes (for example, see [82] for review), but functional studies will be needed to understand development and sex determination in *G. pallida*.

### Conservation of the RNAi pathway in *G. pallida*

RNA interference (RNAi), the process by which double stranded RNA (dsRNA) initiates homology-dependent transcriptional gene silencing, was first described for *C. elegans*[83] where it has become an invaluable tool for functional analysis. Since it was first demonstrated that RNAi could be used to silence genes in J2 cyst nematodes [84] it has been exploited in a range of plant parasitic nematode species both *in vitro*, as a tool for functional genomics, and *in planta* as a strategy for transgenic control. While the technique seems more reliable than for many animal parasitic species [85], inconsistent levels of gene silencing have been reported and the molecular details of the pathways involved have not been elucidated.

A recent study identified 77 *C. elegans* proteins involved in the five key stages of the RNAi pathway [86]. We present a complete catalogue of the repertoire of *G. pallida* genes involved in these processes (Additional file 1: Table S15). Like other parasitic nematodes studied, *G. pallida* contains genes involved in most aspects of the *C. elegans* RNAi pathway, but has fewer genes overall and is particularly deficient in those encoding proteins responsible for uptake of dsRNA and spreading dsRNA between cells to enable systemic RNAi. Many features of the *G. pallida* repertoire appear to be widely conserved in both plant- and animal-parasitic nematodes, such as the conservation of *rsd-3*, thought to be involved in the intercellular distribution of dsRNA following uptake [87] and a reduced total complement of AGO genes in comparison to *C. elegans*[86]. Indeed, in most respects, the RNAi pathway in *G. pallida* appears similar to those described for *M. hapla* and *M. incognita*[86], including similar complements of RNAi inhibitors and nuclear RNAi effectors. *G. pallida* also shares an expansion of genes homologous to *ego-1* RNA-dependent RNA polymerase (RdRP), and expansion of particular AGOs with *B. xylophilus*[24]. Unique features of the *G. pallida* RNAi gene complement include the apparent loss of the Dicer-related helicase, *drh-1*, and loss of a number of components of the *C. elegans* RISC complex, although these remain poorly characterised. The similar RNAi pathways found in *G. pallida* and other parasitic nematode species lack several important components of the *C. elegans* RNAi machinery, suggesting that alternative proteins, or proteins only poorly conserved at the sequence level may be behind the effective, systemic RNAi possible in these species (for example, [84,88,89]).

### Neurotransmission

Despite a relatively simple structure, the nematode nervous system is able to service complex and subtle behavioural responses, accomplished by sophisticated signalling with a diverse array of signalling molecules such as neuropeptides and inherent heterogeneity of receptors for classical neurotransmitters. For example, nematode receptors for acetylcholine (ACh) and glutamate consist of distinct subunits that can assemble in multiple combinations to provide a high degree of receptor plasticity. Beside its inherent interest, the nematode nervous system is a particular target for chemical control methods [90], so greater understanding of the available target molecules may help in the rational design of new nematicides. We present a comprehensive analysis of *G. pallida* neurotransmitter receptors (Additional file 1: Table S16), genes involved in the synthesis, transport and metabolism of neurotransmitters (Additional file 1: Table S17) and genes encoding neuropeptide precursors (Additional file 1: Tables S18, S19; see Supporting Results in Additional file 1 for a detailed description). Genes responsible for the production and utilisation of the neurotransmitters ACh, serotonin, dopamine, tyramine, octopamine, glutamate and gamma-aminobutyric acid (GABA) are all present in *G. pallida* with a very similar complement to *C. elegans*. Similarly, most subtypes of neurotransmitter receptors found in *C. elegans* are present in *G. pallida*, but there are differences in the complement of particular types*. G. pallida* has a somewhat smaller repertoire of nicotinic acetylcholine receptors (nAChRs) than *C. elegans*, with a particularly reduced number of ACR-16 class receptors. It does, however, contain members of each of the five distinct groups of nAChRs [91] and operon organisation of some of these genes (*acr-2* and *acr-3*, *des-2* and *deg-3*) appears conserved.

## Conclusions

*Globodera pallida* is an economically important pathogen of potatoes, as well as a key model system for understanding the biology of cyst nematodes, one of the most important groups of plant pathogens worldwide. The analysis presented here for *G. pallida* is, to our knowledge, the first description of the genome organisation and content of a cyst nematode, complementing the previously characterised genomes of root-knot nematodes. We describe gene expression changes throughout the *G. pallida* lifecycle, including eight different life-stages - among the most comprehensive data available for any parasitic nematode. The combined genome and transcriptome dataset represents a vital platform in understanding the biology of cyst nematodes*,* enabling generation of testable hypotheses about gene function and offering valuable insight into many key processes associated with the parasitic lifestyle.

Biotrophic plant parasitism has arisen independently in cyst and root-knot nematodes, with convergent evolution resulting in the two sedentary endoparasites that induce functionally similar feeding sites. We describe the repertoire of known effector gene families, and exploit our expression data to predict novel effector classes, confirming the distinctive nature of biotrophic parasitism in cyst nematodes. The set of *G. pallida* effectors is strikingly distinct from those previously described in root knot nematodes. Further investigation of this complement of effectors is likely to reveal the genetic basis of the detailed differences in the induced feeding sites of cyst and root-knot nematodes, the greater host specificity of cyst nematodes and the virulence characteristics of *G. pallida* towards different host cultivars. This knowledge will help inform new technologies to control *G. pallida*, and we have described the genetic basis of key nematode biological processes such as neurotransmission, sex determination and diapause that are targets of intervention for the development of new nematode control or management strategies. For example, heterologous expression of *G. pallida* receptors will now be possible to enable functional characterisation and testing of specific chemicals aimed at their disruption. The RNAi pathway is of interest as a target for control and is also a key technology both in functional genomics and in development of transgenic plants that express dsRNAs to target genes essential to the nematode.

Our transcriptome data allow us to go well beyond a genomic ‘parts list’ of proteins and genetic elements that underlie organism function, as the temporal pattern of gene expression gives vital clues to the roles genes play in different processes. The next step is to fully understand how these parts function and interact to cause plant parasitism. Genomic data are becoming key in the fight against a number of groups of plant pathogens [92-94]. The publication of a cyst nematode genome sequence opens the door to applying post-genomic technologies to this important group.

## Materials and methods

### Biological material and nucleic acids extraction

The *G. pallida* population ‘Lindley’, a standard Pa2/3 pathotype held at the James Hutton Institute, Dundee, UK [95] was used to provide source biological material for both DNA and RNA extraction. Cysts were extracted after 10 to 12 weeks of growth of nematodes on host potato plants, and pooled eggs from multiple cysts used for genomic DNA isolation. Total RNA was extracted from eggs of *G. pallida*, freshly hatched J2s, parasitic stages at 7, 14, 21, 28 and 35 dpi, and adult males. Two RNA samples of 5 to 10 μg were produced for RNA-seq of each life-stage, with each replicate sample derived from pooled nematodes collected on multiple occasions. See Supporting Methods in Additional file 1 for full details of all methods.

### Genome sequencing and assembly

We assembled a draft sequence of the *G. pallida* genome based on data from a mixture of sequencing technologies. Additional file 1: Table S1 gives full details of the sequencing libraries used. Genomic and transcriptomic sequence data were generated using largely standard molecular biology methods, except that whole-genome amplified (WGA) material was used to generate sufficient DNA for some libraries (see Supporting Methods in Additional file 1). However, analysis of WGA DNA sequence revealed that the amplification technique used had introduced large numbers of inverted repeats into the amplified material. The vast majority of the sequence data generated from this material therefore had to be discarded. Sequence reads from each technology were initially assembled independently using assembly algorithms most suited to the typical coverage and read length of each, followed by a process of merging, scaffolding with long-insert read pair data from the Roche and Illumina platforms and improvement by automated gap-filling and error correction. *G. pallida* is an obligate parasite, and so cannot be cultured axenically, and highly inbred material is not available. The initial assembly thus contained contamination from both fungal and bacterial sources, as well as a small number of contigs likely to represent haplotypic variants of other contigs in the assembly, which were removed in a conservative approach. Full details of the assembly construction and cleaning are presented in the Supporting Methods section in Additional file 1.

### Protein coding gene prediction, functional and comparative annotation

Protein-coding genes were predicted using Augustus [96], trained with manually curated gene models and using evidence from mapped RNA-seq data. Functional annotation information came from sequence similarity searches, Interproscan [97] and Blast2GO [98] together with manual annotation and additional approaches specific to particular functional categories. Comparative analysis of protein-coding genes between nematode genomes was based on OrthoMCL [99] (called gene families above) and a stand-alone version of the OMA algorithm [100] (called one-to-one ortholog groups). Additional details are presented in the Supporting Methods section in Additional file 1. A total of 2,966 EST clusters were obtained from NEMBASE4 [101] and mapped against the *G. pallida* genome assembly using nucmer version 3.07, keeping hits with at least 95% nucleotide identity.

### Gene expression analysis

Analysis of RNA-seq data was based on counting reads mapping to each protein-coding gene model. Values for relative expression between stages and counts of expressed genes were based on mean RPKM values across the two replicate samples for each life stage. Descriptions of genes as being up- or downregulated between life stages are based on statistical analysis of RNA-seq data using pairwise tests for significant differential expression between stages. We also used model-based clustering of genes to identify sets of genes with similar gene expression dynamics across the stages. See Supporting Methods in Additional file 1 for full details.

### Data access

Sequence data described in this paper have been submitted to the Genbank database. Data and annotation have been submitted to Wormbase and Genbank. The *G. pallida* genome assembly and functional annotation is available from ftp://ftp.sanger.ac.uk/pub/project/pathogens/Globodera/pallida and via GeneDB at http://www.genedb.org/Homepage/Gpallida. Raw sequence reads are available from the ENA SRA as listed in Supporting Information.

## Abbreviations

ABC: ATP-binding cassette; ACh: Acetylcholine; AGO: Argonaute protein; CYP: Cytochrome P450; dpi: Days post infection; ER: Endoplasmic reticulum; GPCR: G protein-coupled receptor; GST: glutathione-S-transferase; J1 to J4: First- to fourth-stage juvenile; nAChR: Nicotinic acetylcholine-gated receptor; NHR: Nuclear hormone receptor; PCN: Potato cyst nematode; RISC: RNA-induced silencing complex; RNAi: RNA interference; SL: Spliced leader; SOD: Superoxide dismutase; SPRYSEC: Secreted protein with a SPRY domain; UGT: UDP-glucuronosyl transferase.

## Competing interests

The authors declare that they have no competing interests.

## Authors’ contributions

MB, JTJ and PEU conceived and designed the research that was coordinated by MB, CJL, NH, HB, AP, JTJ and PEU. CJL and VB collected the biological material and prepared DNA and RNA. JAC, CJL, TK, AJR, PT, IJT, VB, PJAC, SEvdA, MH, LMJ, SM, HN, MZ and JEP-R analysed the data. MB, JAC, CJL, JTJ and PEU drafted the complete manuscript with contributions from the other authors. All authors read and approved the final manuscript.

## Supplementary Material

Additional file 1 Supporting Methods, Results, Figures and Tables. Click here for file

Additional file 2 **Tables of genes with significant differences in expression between life-stages of *Globodera pallida*.** Click here for file

## References

[B1] Decraemer W Hunt DJ Perry RN, Moens M Structure and classification Plant Nematology 2006 Wallingford: CABI Publishing 3 32

[B2] Nicol JM Turner SJ Coyne DL den Nijs L Hockland S Maafi ZT Jones JT, Gheysen G, Fenoll C Current nematode threats to world agriculture Genomics and Molecular Genetics of Plant-nematode Interactions 2011 Dordrecht, The Netherlands: Springer 21 43

[B3] Perry RN Moens M Jones JT, Gheysen G, Fenoll C Introduction to plant-parasitic nematodes: modes of parasitism Genomics and Molecular Genetics of Plant-nematode Interactions 2011 Dordrecht, The Netherlands: Springer 3 20

[B4] Baldwin JG Nadler SA Adams BJ Evolution of plant parasitism among nematodes Ann Rev Phytopathol 2004 42 83 105 10.1146/annurev.phyto.42.012204.130804 15283661

[B5] Trudgill DL Yield losses caused by potato cyst nematodes - a review of the current position in Britain and prospects for improvements Ann Appl Biol 1986 108 181 198 10.1111/j.1744-7348.1986.tb01979.x

[B6] Evans K Franco J Descurrah MM Distribution of species of potato cyst nematodes in South America Nematologica 1975 21 365 369 10.1163/187529275X00103

[B7] Hockland S Niere B Grenier E Blok V Phillips M Den Nijs L Anthoine G Pickup J Viaene N **An evaluation of the implications of virulence in non-European populations of **** *Globodera pallida * ****and **** *G. rostochiensis * ****for potato cultivation in Europe.** Nematol 2012 14 1 13 10.1163/138855411X587112

[B8] Pylypenko LA Uehara T Phillips MS Sigareva DD Blok VC Identification of *Globodera rostochiensis* and *G. pallida* in the Ukraine by PCR Eur J Plant Pathol 2005 111 39 46 10.1007/s10658-004-2732-9

[B9] Skantar AM Handoo ZA Carta LK Chitwood DJ Morphological and molecular identification of *Globodera pallida* associated with potato in Idaho J Nematol 2007 39 133 144 19259482 PMC2586493

[B10] Minnis ST Haydock PPJ Ibrahim SK Grove IG Evans K Russell MD Potato cyst nematodes in England and Wales-occurrence and distribution Ann Appl Biol 2002 140 187 195 10.1111/j.1744-7348.2002.tb00172.x

[B11] Turner SJ Population decline of potato cyst nematodes (*Globodera rostochiensis*, *G. pallida*) in field soils in Northern Ireland Ann Appl Biol 1996 129 315 322 10.1111/j.1744-7348.1996.tb05754.x

[B12] Trudgill DL Phillips MS Elliott MJ Dynamics and management of the white potato cyst nematode *Globodera pallida* in commercial potato crops Ann Appl Biol 2014 164 18 34 10.1111/aab.12085

[B13] Clayton R Storey M Parker B Ballingall M Davies K Impact of reduced pesticide availability on control of potato cyst nematodes and weeds in potato crops 2008 Kenilworth: Potato Council Ltd.

[B14] Lilley CJ Atkinson HJ Urwin PE Molecular aspects of cyst nematodes Mol Plant Pathol 2005 6 577 588 10.1111/j.1364-3703.2005.00306.x 20565681

[B15] Sobczak M Golinowski W Jones JT, Gheysen G, Fenoll C Cyst nematodes and syncytia Genomics and Molecular Genetics of Plant-nematode Interactions 2011 Dordrecht, The Netherlands: Springer 61 82

[B16] C. elegans sequencing consortium Genome sequence of the nematode *C. elegans*: a platform for investigating biology Science 1998 282 2012 2018 9851916 10.1126/science.282.5396.2012

[B17] Ghedin E Wang SL Spiro D Caler E Zhao Q Crabtree J Allen JE Delcher AL Guiliano DB Miranda-Saavedra D Angiuoli SV Creasy T Amedeo P Haas B El-Sayed NM Wortman JR Feldblyum T Tallon L Schatz M Shumway M Koo H Salzberg SL Schobel S Pertea M Pop M White O Barton GJ Carlow CKS Crawford MJ Daub J Draft genome of the filarial nematode parasite *Brugia malayi* Science 2007 317 1756 1760 10.1126/science.1145406 17885136 PMC2613796

[B18] Blaxter M Kumar S Kaur G Koutsovoulos G Elsworth B Genomics and transcriptomics across the diversity of the nematoda Parasite Immunol 2012 34 108 120 10.1111/j.1365-3024.2011.01342.x 22044053

[B19] Godel C Kumar S Koutsovoulos G Ludin P Nilsson D Comandatore F Wrobel N Thompson M Schmid CD Goto S Bringaud F Wolstenholme A Bandi C Epe C Kaminsky R Blaxter M Mäser P The genome of the heartworm, *Dirofilaria immitis*, reveals drug and vaccine targets FASEB J 2012 26 4650 4661 10.1096/fj.12-205096 22889830 PMC3475251

[B20] Desjardins CA Cerqueira GC Goldberg JM Dunning Hotopp JC Haas BJ Zucker J Ribeiro JM Saif S Levin JZ Fan L Zeng Q Russ C Wortman JR Fink DL Birren BW Nutman TB Genomics of *Loa loa*, a Wolbachia-free filarial parasite of humans Nat Genet 2013 45 495 500 10.1038/ng.2585 23525074 PMC4238225

[B21] Bird DM Williamson VM Abad P McCarter J Danchin EGJ Castagnone-Sereno P Opperman CH The genomes of root-knot nematodes Ann Rev Phytopathol 2009 47 333 351 10.1146/annurev-phyto-080508-081839 19400640

[B22] Abad P Gouzy J Aury JM Castagnone-Sereno P Danchin EG Deleury E Perfus-Barbeoch L Anthouard V Artiguenave F Blok VC Caillaud M-C Coutinho PM Dasilva C De Luca F Deau F Esquibet M Flutre T Goldstone JV Hamamouch N Hewezi T Jaillon O Jubin C Leonetti P Magliano M Maier TR Markov GV McVeigh P Pesole G Poulain J Robinson-Rechavi M Genome sequence of the metazoan plant-parasitic nematode *Meloidogyne incognita* Nat Biotechnol 2008 26 909 915 10.1038/nbt.1482 18660804

[B23] Opperman CH Bird DM Williamson VM Rokhsar DS Burke M Cohn J Cromer J Diener S Gajan J Graham S Houfek TD Liu Q Mitros T Schaff J Schaffer R Scholl E Sosinski BR Thomas VP Windham E Sequence and genetic map of *Meloidogyne hapla*: a compact nematode genome for plant parasitism Proc Natl Acad Sci U S A 2008 105 14802 14807 10.1073/pnas.0805946105 18809916 PMC2547418

[B24] Kikuchi T Cotton JA Dalzell JJ Hasegawa K Kanzaki N McVeigh P Takanashi T Tsai IJ Assefa SA Cock PJ Otto TD Hunt M Reid AJ Sanchez-Flores A Tsuchihara K Yokoi T Larsson MC Miwa J Maule AG Sahashi N Jones JT Berriman M Genomic insights into the origin of parasitism in the emerging plant pathogen *Bursaphelenchus xylophilus* PLoS Pathog 2011 7 e1002219 10.1371/journal.ppat.1002219 21909270 PMC3164644

[B25] Hoolahan AH Blok VC Gibson T Dowton M A comparison of three molecular markers for the identification of populations of *Globodera pallida* J Nematol 2012 44 7 17 23482966 PMC3593263

[B26] Stranneheim H Lundeberg J Stepping stones in DNA sequencing Biotechnol J 2012 7 1063 1073 10.1002/biot.201200153 22887891 PMC3472021

[B27] Peters BA Kermani BG Sparks AB Alferov O Hong P Alexeev A Jiang Y Dahl F Tang YT Haas J Robasky K Zaranek AW Lee J-H Ball MP Peterson JE Perazich H Yeung G Liu J Chen L Kennemer MI Pothuraju K Konvicka K Tsoupko-Sitnikov M Pant KP Ebert JC Nilsen GB Baccash J Halpern AL Church GM Drmanac R Accurate whole-genome sequencing and haplotyping from 10 to 20 human cells Nature 2012 487 190 195 10.1038/nature11236 22785314 PMC3397394

[B28] Mitreva M Jasmer DP Zarlenga DS Wang Z Abubucker S Martin J Taylor CM Yin Y Fulton L Minx P Yang S-P Warren WC Fulton RS Bhonagiri V Zhang X Hallsworth-Pepin K Clifton SW McCarter JP Appleton J Mardis ER Wilson RK The draft genome of the parasitic nematode *Trichinella spiralis* Nat Genet 2011 43 228 235 10.1038/ng.769 21336279 PMC3057868

[B29] Grisi E Burrows PR Perry RN Hominick W The genome size and chromosome complement of the potato cyst nematode *Globodera pallida* Fund Appl Nematol 1995 18 67 70

[B30] van der Voort JNAMR van Enckevort ELJG Pijnacker LP Helder J Gommers FJ Bakker J Chromosome number of the potato cyst nematode *Globodera rostochiensis* Fund Appl Nematol 1996 19 369 374

[B31] Merlin J Goldstein A Goldstein P Three-dimensional ultrastructural karyotype analysis from the meiotic parthenogenetic nematode *Heterodera betulae* J Nematol 2003 35 228 231 19266000 PMC2620618

[B32] Goldstein P Triantaphyllou AC Karyotype analysis of the plant-parasitic nematode *Heterodera glycines* by electron microscopy 1 Diploid. J Cell Sci 1979 40 171 179 10.1242/jcs.40.1.171 536384

[B33] Goldstein P Triantaphyllou AC Karyotype analysis of *Meloidogyne hapla* by 3-D reconstruction of synaptonemal complexes from electron microscopy of serial sections Chromosoma 1978 70 131 139 10.1007/BF00292221

[B34] Coghlan A The C. elegans Research Community Nematode genome evolution WormBookhttp://www.wormbook.org

[B35] Thomas VP Fudali SL Schaff JE Liu QL Scholl EH Opperman CH Bird DM Williamson VM **sequence-anchored linkage map of the plant-parasitic nematode **** *Meloidogyne * **** *hapla * ****reveals exceptionally high genome-wide recombination. G3-Genes Genomes.** G3-Genes Genomes Genetics 2012 2 815 824 10.1534/g3.112.002261 PMC3385987 22870404

[B36] Keeling PJ Reduction and compaction in the genome of the apicomplexan parasite *Cryptosporidium parvum* Dev Cell 2004 6 614 616 10.1016/S1534-5807(04)00135-2 15130487

[B37] Animal genome size database http://www.genomesize.com

[B38] Leroy S Bouamer S Morand S Fargette M Genome size of plant-parasitic nematodes Nematol 2007 9 449 450 10.1163/156854107781352089

[B39] Costa JC Lilley CJ Urwin PE *Caenorhabditis elegans* as a model for plant-parasitic nematodes Nematol 2007 9 3 16 10.1163/156854107779969664

[B40] Jones LM De Giorgi C Urwin PE Jones JT, Gheysen G, Fenoll C *C. elegans* as a resource for studies on plant parasitic nematodes Genomics and Molecular Genetics of Plant-nematode Interactions 2011 Dordrecht, The Netherlands: Springer 175 220

[B41] Allen MA Hillier LW Waterston RH Blumenthal T A global analysis of *C. elegans* trans-splicing Genome Res 2011 21 255 264 10.1101/gr.113811.110 21177958 PMC3032929

[B42] van Bers NEM Characterization of genes coding for small hypervariable peptides in Globodera rostochiensis, PhD thesis 2008 Wageningen: Wageningen University

[B43] Kass J Jacob TC Kim P Kaplan JM The EGL-3 proprotein convertase regulates mechanosensory responses of *Caenorhabditis elegans* J Neurosci 2001 21 9265 9272 11717360 10.1523/JNEUROSCI.21-23-09265.2001 PMC6763909

[B44] Shakir MA Fukushige T Yasuda H Miwa J Siddiqui SS** *C. elegans osm-3 gene * ****mediating osmotic avoidance behaviour encodes a kinesin-like protein.** Neuroreport 1993 4 891 894 10.1097/00001756-199307000-00013 7690265

[B45] Salkoff L Wei AD Baban B Butler A Fawcett G Ferreira G Santi CM The C. elegans Research Community Potassium channels in *C. elegans* WormBookhttp://www.wormbook.org 10.1895/wormbook.1.42.1 PMC4781360 18050399

[B46] Behm CA The role of trehalose in the physiology of nematodes Int J Parasitol 1997 27 215 229 10.1016/S0020-7519(96)00151-8 9088992

[B47] Jones JT Kumar A Pylypenko LA Thirugnanasambandam A Castelli L Chapman S Cock PJA Grenier E Lilley CJ Phillips MS Blok VC Identification and functional characterization of effectors in expressed sequence tags from various life cycle stages of the potato cyst nematode *Globodera pallida* Mol Plant Pathol 2009 10 815 828 10.1111/j.1364-3703.2009.00585.x 19849787 PMC6640342

[B48] Qin L Overmars B Helder J Popeijus H van der Voort JR Groenink W van Koert P Schots A Bakker J Smant G An efficient cDNA-AFLP-based strategy for the identification of putative pathogenicity factors from the potato cyst nematode *Globodera rostochiensis* Mol Plant-Microbe Int 2000 13 830 836 10.1094/MPMI.2000.13.8.830 10939254

[B49] Gao BL Allen R Maier T Davis EL Baum TJ Hussey RS The parasitome of the phytonematode *Heterodera glycines* Mol Plant-Microbe Int 2003 16 720 726 10.1094/MPMI.2003.16.8.720 12906116

[B50] van Megen H van den Elsen S Holterman M Karssen G Mooyman P Bongers T Holovachov O Bakker J Helder J A phylogenetic tree of nematodes based on about 1200 full-length small subunit ribosomal DNA sequences Nematology 2009 11 927 950 10.1163/156854109X456862

[B51] Haegeman A Jones JT Danchin EGJ Horizontal gene transfer in nematodes: A catalyst for plant parasitism? Mol Plant-Microbe Int 2011 24 879 887 10.1094/MPMI-03-11-0055 21539433

[B52] Vanholme B Haegeman A Jacob J Cannoot B Gheysen G Arabinogalactan endo-1,4-β-galactosidase: a putative cell wall-degrading enzyme of plant-parasitic nematodes Nematology 2009 11 739 747 10.1163/156854109X404599

[B53] Jones JT Furlanetto C Bakker E Banks B Blok V Chen Q Phillips M Prior A Characterization of a chorismate mutase from the potato cyst nematode *Globodera pallida* Mol Plant Pathol 2003 4 43 50 10.1046/j.1364-3703.2003.00140.x 20569361

[B54] Craig JP Bekal S Niblack T Domier L Lambert KN Evidence for horizontally transferred genes involved in the biosynthesis of vitamin B-1, B-5, and B-7 in *Heterodera glycines* J Nematol 2009 41 281 290 22736827 PMC3381462

[B55] Hussey RS Mimms CW Ultrastructure of esophogeal glands and their secretory granules in the root-knot nematode *Meloidogyne incognita* Protoplasma 1990 162 99 107

[B56] Smant G Goverse A Stokkermans JPWG De Boer JM Pomp H Zilverentant JF Overmars HA Helder J Schots A Bakker J Potato root diffusate-induced secretion of soluble, basic proteins originating from the subventral esophageal glands of potato cyst nematodes Phytopathology 1997 87 839 845 10.1094/PHYTO.1997.87.8.839 18945052

[B57] Lu SW Chen S Wang J Yu H Chronis D Mitchum MG Wang X Structural and functional diversity of CLAVATA3/ESR (CLE)-like genes from the potato cyst nematode *Globodera rostochiensis* Mol Plant Microbe Int 2009 22 1128 1142 10.1094/MPMI-22-9-1128 19656047

[B58] Rehman S Postma W Tytgat T Prins P Qin L Overmars H Vossen J Spiridon LN Petrescu AJ Goverse A Bakker J Smant G A secreted SPRY domain-containing protein (SPRYSEC) from the plant-parasitic nematode *Globodera rostochiensis* interacts with a CC-NB-LRR protein from a susceptible tomato Mol Plant-Microbe Int 2009 22 330 340 10.1094/MPMI-22-3-0330 19245327

[B59] Sacco MA Koropacka K Grenier E Jaubert MJ Blanchard A Goverse A Smant G Moffett P The cyst nematode SPRYSEC protein RBP-1 elicits Gpa2- and RanGAP2-dependent plant cell death PLoS Pathog 2009 5 e1000564 10.1371/journal.ppat.1000564 19714238 PMC2727447

[B60] O'Brien JA Daudi A Butt VS Bolwell GP Reactive oxygen species and their role in plant defence and cell wall metabolism Planta 2012 236 765 779 10.1007/s00425-012-1696-9 22767200

[B61] Cabreiro F Ackerman D Doonan R Araiz C Back P Papp D Braeckman BP Gems D Increased life span from overexpression of superoxide dismutase in *Caenorhabditis elegans* is not caused by decreased oxidative damage Free Radical Bio Med 2011 51 1575 1582 10.1016/j.freeradbiomed.2011.07.020 21839827 PMC3202636

[B62] Robertson L Robertson WM Sobczak M Bakker J Tetaud E Arinagayayam MR Ferguson MAJ Fairlamb AH Jones JT Cloning, expression and functional characterisation of a thioredoxin peroxidase from the potato cyst nematode *Globodera rostochiensis* Mol Biochem Parasitol 2000 111 41 49 10.1016/S0166-6851(00)00295-4 11087915

[B63] Jones JT Reavy B Smant G Prior AE Glutathione peroxidases of the potato cyst nematode *Globodera rostochiensis* Gene 2004 324 47 54 14693370 10.1016/j.gene.2003.09.051

[B64] Noctor G Mhamdi A Chaouch S Han Y Neukermans J Marquez-Garcia B Queval G Foyer CH Glutathione in plants: an integrated overview Plant Cell Environ 2012 35 454 484 10.1111/j.1365-3040.2011.02400.x 21777251

[B65] Baldacci-Cresp F Chang C Maucourt M Deborde C Hopkins J Lecomte P Bernillon S Brouquisse R Moing A Abad P Hérouart D Puppo A Favery B Frendo P (Homo)glutathione deficiency impairs root-knot nematode development in *Medicago truncatula* PLoS Pathog 2012 8 e1002471 10.1371/journal.ppat.1002471 22241996 PMC3252378

[B66] Lu SC Glutathione synthesis Biochim Biophys Acta 2013 1830 3143 3153 10.1016/j.bbagen.2012.09.008 22995213 PMC3549305

[B67] Jirschitzka J Schmidt GW Reichelt M Schneider B Gershenzon J D'Auria JC Plant tropane alkaloid biosynthesis evolved independently in the Solanaceae and Erythroxylaceae Proc Natl Acad Sci U S A 2012 109 10304 10309 10.1073/pnas.1200473109 22665766 PMC3387132

[B68] Lindblom TH Dodd AK Xenobiotic detoxification in the nematode *Caenorhabditis elegans* J Exp Zool A Comp Exp Biol 2006 305 720 730 16902959 10.1002/jez.a.324 PMC2656347

[B69] Laing ST Ivens A Butler V Ravikumar SP Laing R Woods DJ Gilleard JS The transcriptional response of *Caenorhabditis elegans* to ivermectin exposure identifies novel genes involved in the response to reduced food intake PLoS ONE 2012 7 e31367 10.1371/journal.pone.0031367 22348077 PMC3279368

[B70] van Rossum AJ Brophy PM Tait A Barrett J Jefferies JR Proteomic identification of glutathione S-transferases from the model nematode *Caenorhabditis elegans* Proteomics 2001 1 1463 1468 10.1002/1615-9861(200111)1:11<1463::AID-PROT1463>3.0.CO;2-H 11922606

[B71] Robinson-Rechavi M Maina CV Gissendanner CR Laudet V Sluder A Explosive lineage-specific expansion of the orphan nuclear receptor HNF4 in nematodes J Mol Evol 2005 60 577 586 10.1007/s00239-004-0175-8 15983867

[B72] Taubert S Ward JD Yamamoto KR Nuclear hormone receptors in nematodes: evolution and function Mol Cell Endocrinol 2011 334 49 55 10.1016/j.mce.2010.04.021 20438802 PMC3042524

[B73] Zarkower D The C. elegans Research Community Somatic sex determination WormBookhttp://www.wormbook.org

[B74] Mugniery D Fayet G Sex determination in *Globodera pallida* Rev Nematol 1981 4 41 45

[B75] Trudgill DL The effect of environment on sex determination in *Heterodera glycines* Nematologica 1967 13 263 272 10.1163/187529267X00120

[B76] Triantaphyllou AC Environmental sex differentation of nematodes in relation to pest management Ann Rev Phytopathol 1973 11 441 462 10.1146/annurev.py.11.090173.002301

[B77] Phillips M Forrest J Farrer A Invasion and development of juveniles of *Globodera pallida* in hybrids of *Solanum vernei* x *S. tuberosum* Ann Appl Biol 1982 100 337 344 10.1111/j.1744-7348.1982.tb01947.x

[B78] Spears JF The golden nematode handbook: survey, laboratory, control and quarantine procedures 1968 Washington, DC: U.S. Department of Agriculture, Agricultural Research Service

[B79] Elling AA Mitreva M Recknor J Gai X Martin J Maier TR McDermott JP Hewezi T Bird DM Davis EL Hussey RS Nettleton D McCarter JP Baum TJ Divergent evolution of arrested development in the dauer stage of *Caenorhabditis elegans* and the infective stage of *Heterodera glycines* Genome Biol 2007 8 R211 10.1186/gb-2007-8-10-r211 17919324 PMC2246285

[B80] Endo PJ Ultrastructure of the intestine of the second and third juvenile stages of *Heterodera glycines* P Helm Soc Wash 1988 55 117 131

[B81] Hu PJ The C. elegans Research Community Dauer WormBookhttp://www.wormbook.org

[B82] Sommer RJ Homology and the hierarchy of biological systems BioEssays 2008 30 653 658 10.1002/bies.20776 18536034

[B83] Fire A Xu SQ Montgomery MK Kostas SA Driver SE Mello CC Potent and specific genetic interference by double-stranded RNA in *Caenorhabditis elegans* Nature 1998 391 806 811 10.1038/35888 9486653

[B84] Urwin PE Lilley CJ Atkinson HJ Ingestion of double-stranded RNA by preparasitic juvenile cyst nematodes leads to RNA interference Mol Plant-Microbe Interact 2002 15 747 752 10.1094/MPMI.2002.15.8.747 12182331

[B85] Lilley CJ Davies LJ Urwin PE RNA interference in plant parasitic nematodes: a summary of the current status Parasitology 2012 139 630 640 10.1017/S0031182011002071 22217302

[B86] Dalzell JJ McVeigh P Warnock ND Mitreva M Bird DM Abad P Fleming CC Day TA Mousley A Marks NJ Maule AG RNAi effector diversity in nematodes PLoS Negl Trop Dis 2011 5 e1176 10.1371/journal.pntd.0001176 21666793 PMC3110158

[B87] Tijsterman M May RC Simmer F Okihara KL Plasterk RHA Genes required for systemic RNA interference in *Caenorhabditis elegans* Curr Biol 2004 14 111 116 10.1016/j.cub.2003.12.029 14738731

[B88] Dalzell JJ McMaster S Fleming CC Maule AG Short interfering RNA-mediated gene silencing in *Globodera pallida* and *Meloidogyne incognita* infective stage juveniles Int J Parasitol 2010 40 91 100 10.1016/j.ijpara.2009.07.003 19651131

[B89] Kimber MJ McKinney S McMaster S Day TA Fleming CC Maule AG *flp* gene disruption in a parasitic nematode reveals motor dysfunction and unusual neuronal sensitivity to RNA interference FASEB J 2007 21 1233 1243 10.1096/fj.06-7343com 17200420

[B90] Kimber MJ Fleming CC Neuromuscular function in plant parasitic nematodes: a target for novel control strategies? Parasitology 2005 131 129 142 10.1017/S0031182005009157 16569286

[B91] Brown LA Jones AK Buckingham SD Mee CJ Sattelle DB Contributions from *Caenorhabditis elegans* functional genetics to antiparasitic drug target identification and validation: nicotinic acetylcholine receptors, a case study Int J Parasitol 2006 36 617 624 10.1016/j.ijpara.2006.01.016 16620825

[B92] Pais M Win J Yoshida K Etherington GJ Cano LM Raffaele S Banfield MJ Jones A Kamoun S Saunders DGO From pathogen genomes to host plant processes: the power of plant parasitic oomycetes Genome Biol 2013 14 211 10.1186/gb-2013-14-6-211 23809564 PMC3706818

[B93] Soanes DM Richards TA Talbot NJ Insights from sequencing fungal and oomycete genomes: What can we learn about plant disease and the evolution of pathogenicity? Plant Cell 2007 19 3318 3326 10.1105/tpc.107.056663 18024565 PMC2174898

[B94] Spanu PD The genomics of obligate (and nonobligate) biotrophs Ann Rev Phytopathol 2012 50 91 109 10.1146/annurev-phyto-081211-173024 22559067

[B95] Phillips MS Trudgill DL **Variation of virulence, in terms of quantitative reproduction of **** *Globodera pallida * ****populations, from Europe and South America, in relation to resistance from **** *Solanum vernei * ****and**** *S. tuberosum ssp andigena * ****CPC 2802.** Nematologica 1998 44 409 423 10.1163/005525998X00070

[B96] Stanke M Schoffmann O Morgenstern B Waack S Gene prediction in eukaryotes with a generalized hidden Markov model that uses hints from external sources BMC Bioinformatics 2006 7 62 10.1186/1471-2105-7-62 16469098 PMC1409804

[B97] Hunter S Jones P Mitchell A Apweiler R Attwood TK Bateman A Bernard T Binns D Bork P Burge S de Castro E Coggill P Corbett M Das U Daugherty L Duquenne L Finn RD Fraser M Gough J Haft D Hulo N Kahn D Kelly E Letunic I Lonsdale D Lopez R Madera M Maslen J McAnulla C McDowall J InterPro in 2011: new developments in the family and domain prediction database Nucl Acids Res 2012 40 D306 D312 10.1093/nar/gkr948 22096229 PMC3245097

[B98] Conesa A Gotz S Garcia-Gomez JM Terol J Talon M Robles M Blast2GO: a universal tool for annotation, visualization and analysis in functional genomics research Bioinformatics 2005 21 3674 3676 10.1093/bioinformatics/bti610 16081474

[B99] Li L Stoeckert CJ Roos DS OrthoMCL: identification of ortholog groups for eukaryotic genomes Genome Res 2003 13 2178 2189 10.1101/gr.1224503 12952885 PMC403725

[B100] Roth AC Gonnet GH Dessimoz C Algorithm of OMA for large-scale orthology inference BMC Bioinformatics 2008 9 518 10.1186/1471-2105-9-518 19055798 PMC2639434

[B101] Elsworth B Wasmuth J Blaxter M NEMBASE4: the nematode transcriptome resource Int J Parasitol 2011 41 881 894 10.1016/j.ijpara.2011.03.009 21550347

