# Supplementary material for: The genome and life-stage specific transcriptomes of Globodera pallida elucidate key aspects of plant parasitism by a cyst nematode
Source: Genome Biol. 2014 Mar 3;15(3):R43. doi: 10.1186/gb-2014-15-3-r43 (PMC4054857; doi:10.1186/gb-2014-15-3-r43)
Supplement: Additional file 1 — Supporting Methods, Results, Figures and Tables. [file gb-2014-15-3-r43-S1.doc]

**SUPPORTING INFORMATION**

**CONTENTS**

Method S1. Biological material and nucleic acids extraction

Method S2. Sequencing and library construction

Method S3. Sequence assembly

Method S4. Gene prediction and annotation

Method S5. Comparative genomics analysis

Method S6. RNA-seq analysis

Method S7. Identifying and annotating repeats

Method S8. Annotation and analysis of functional gene categories

Results S1. Analysis of spliced leaders, operons, RNAi pathway genes and genes involved in neurotransmission.

Figure S1. Flowchart of *Globodera pallida* assembly process.

Figure S2. GC content and taxonomic distribution of contigs in *Globodera pallida* assembly at different stages of contamination filtering.

Figure S3. Intestinal expression of one member of the *Globodera pallida* “dorsal gland-specific” gene family.

Figure S4. Frequency distribution of expression correlation between pairs of *Globodera pallida* genes.

Figure S5. Global variation in expression levels across *Globodera pallida* lifecycle stages.

Figure S6. Clustering of genes by expression dynamics.

Figure S7. Expression levels of diapause-related genes.

Figure S8. Heatmap showing similarity of different transcriptome libraries.

Table S1. Genomic sequencing libraries included in the assembly.

Table S2. Genome and gene model statistics for *Globodera pallida* compared to those for other published nematode genomes.

Table S3. Summary of repeat families in the *Globodera pallida* genome.

Table S4. Transcriptome (RNA-seq) sequencing libraries.

Table S5. Functional properties of *Globodera pallida*-restricted proteins.

Table S6. RNA-seq evidence for diverse spliced leader sequences.

Table S7. *Globodera pallida* effectors similar to effectors from other plant-parasitic nematodes.

Table S8. Cell wall modifying proteins in *Globodera pallida.*

Table S9. *Globodera pallida* proteins containing a SPRY domain, including SPRYSECS.

Table S10. Novel *Globodera pallida* secreted proteins up-regulated in J2 or early parasitic stages that may represent novel effector candidates.

Table S11. Comparison of putative detoxification genes identified in *Globodera pallida* with those found in *Meloidogyne incognita* and *Caenorhabditis elegans*.

Table S12. Presence of *C. elegans* immune response genes in *Globodera pallida* and other organisms.

Table S13. Comparison of nuclear hormone receptors identified in *Globodera pallida* with those found in other organisms.

Table S14. *Globodera pallida* orthologs and genes with high similarity to *Caenorhabditis elegans* genes related to diapause*.*

Table S15. Presence of *C. elegans* RNAi pathway genes in *Globodera pallida* and other nematodes.

Table S16. Comparison of neurotransmitter receptor families between *Caenorhabditis elegans* and *Globodera pallida*.

Table S17. Presence of neurotransmitter biosynthesis, transport and metabolism genes in *Globodera pallida.*

Table S18. Presence of *flp* neuropeptide-encoding genes in *G. pallida* and comparison with *M. incognita* and *B. xylophilus*.

Table S19. Presence of *nlp* neuropeptide-encoding genes in *Globodera pallida* and comparison with *Meloidogyne incognita* and *Bursaphelenchus xylophilus*.

**SUPPORTING METHODS**

**1. Biological material and nucleic acids extraction**

*Globodera pallida* nematodes were cultured on potato plants (*Solanum tuberosum* ‘Desiree’) grown in a 50:50 mix of sterilised sand and loam soil infested with cysts at approximately 25 eggs/g. After 10-12 weeks of growth, the soil was dried and cysts were extracted by flotation using a Fenwick can. Healthy, undamaged cysts were used for extraction of eggs by either gentle crushing in sterile water or release following treatment of the cysts in 1 % sodium hypochlorite. Eggs were cleaned by flotation on 1:1 (w/v) sucrose followed by extensive washes in sterile distilled water. Egg preparations were checked for the presence of obvious contaminating material and then used for DNA extractions. Genomic DNA was extracted from 50 µl packed volume aliquots of *G. pallida* eggs according to the method for small scale preparation of DNA from *C. elegans* as described by Sulston and Hodgkin . For collection of the sterile material that provided DNA for whole genome amplification (WGA), cysts were first treated with 0.1 % malachite green for 1 h then washed extensively and incubated for 24 h in an antibiotic cocktail . After 5-6 washes in sterile tap water, individual cysts were transferred to the wells of a sterile 96-well plate each containing 150 μl of filter-sterilised potato root diffusate and incubated at 20 ^o^C. Hatched 2^nd^-stage juvenile (J2) nematodes were collected separately from each cyst and treated with 0.1 % (v/v) chlorhexidine digluconate and 0.5 mg/ml hexadecyltrimethylammonium bromide for 30 mins. J2 were pelleted by brief centrifugation and washed three times in sterile 0.01% Tween-20. The sibling J2s from each cyst were used to infect individual potato plantlets maintained on Murashige and Skoog basal medium (Duchefa) with 2 % sucrose in 9 cm tissue culture dishes. Approximately 35 J2 were applied on a square of GF/A filter (Whatman, Maidstone, UK) to each of three root tips per plantlet. The filters were removed after 48 h and pairs of young sibling female nematodes were dissected from the roots after 14-17 days. DNA was extracted from each pair of nematodes using a QIAamp DNA micro kit (Qiagen, Crawley, Sussex, UK)

Total RNA was extracted from eggs of *G. pallida*, freshly hatched J2s, parasitic stages at 7, 14, 21, 28 and 35 days post infection (dpi) and adult males. Eggs were collected by gently crushing intact cysts in sterile water. Second stage juveniles were hatched from cysts in tomato root diffusate as described previously . Eggs and J2s were cleaned by flotation on 1:1 (w/v) sucrose in sterile distilled water.

For the parasitic stages, root tips of potato plantlets in growth pouches (Mega International, MN, USA) were infected with hatched J2s of *G. pallida*. Approximately 5 root tips per plant were each infected with 25 J2 of *G. pallida* applied on a 1cm^2^ GF/A filter (Whatman). The GF/A paper was removed after 24 h to aid synchronous infection. Plants were maintained in a growth chamber (MLR350 Environmental Test Chamber; Sanyo, Herts., UK) at 20ºC under 16 h/8 h light/dark cycles. The average light intensity was 140 µm/m^2^/s with a humidity of approximately 30%. For 14 dpi-35 dpi worms, the roots were examined under a stereobinocular microscope, nematodes were individually dissected using needles and fine forceps, and collected into a watch glass of tap water kept on ice. Any damaged or unhealthy worms and any that had significantly delayed development compared to the most advanced worms at that time point were discarded. Nematodes were then carefully cleaned to remove any adhering plant material by gently moving each worm through sterile 1 % water agar.

For 7 dpi nematodes the plant roots were blended briefly in water and the released early parasitic stages collected on a 30 µm sieve. Nematodes were then handpicked from debris into a watch glass as above and cleaned by successive transfers through sterile tap water. Adult male nematodes were collected from potato plants grown and infected in sand/loam mix as described above. Root systems of 3-4 week old plants were washed and male worms collected from roots suspended in aerated tap water as described previously . Nematodes of all stages were collected in 1.5 ml microcentrifuge tubes and flash frozen immediately after collection prior to storage at -80 ^o^C.

Total RNA was extracted from nematode samples using the RNeasy Mini Kit (Qiagen) with on-column DNase I treatment. Two RNA samples of 5-10 µg were produced for RNA-seq of each life-stage, with each replicate sample derived from pooled nematodes collected on multiple occasions.

**2. Sequencing and library construction**

(a) Capillary libraries

Plasmid (pOTW12 and pMAQ1Sac_BstXI) and fosmid (pCC1Fos) libraries containing a range of fragment sizes (Table S1A) of *G. pallida* genomic DNA were cultured in 96-well plates. After DNA extraction using standard protocols, clones were end-sequenced using ABI BigDye version 3.1 with standard primers and analysed on an ABI 3730 Capillary DNA Analyser.

(b) 454 libraries

Paired-end (3 kb, 8 kb and 20 kb) and shotgun 454 libraries (Table S1B) were generated using standard Roche protocols (www.454.com) and sequenced using the 454 Life Sciences GS-20 and GS-FLX sequencer (Roche).

(c) Illumina libraries

Genomic DNA was quantified on the Invitrogen Qubit and then sheared into 200-300 bp and 300-400 bp fragments using Covaris Adaptive Focused Acoustics technology (AFA). This was followed by end repair with T4 and Klenow DNA polymerases and T4 polynucleotide kinase to blunt-end the DNA fragments. A single 3’ A nucleotide was added to the repaired ends using Klenow exo- and dATP to deter concatemerization of templates, limit adapter dimers and increase the efficiency of adapter ligation. PE duplex adapter was ligated using a fast T4 DNA ligase. Ligated fragments were run on an agarose gel, size selected and DNA extracted using a gel extraction kit (Qiagen) according to the manufacturer’s protocol but with dissolution of gel slices at room temperature (rather than 50 ^o^C) to avoid heat induced bias. Extracted molecules were subjected to PCR using primers PE1.0 and PE2.0 for 8 cycles with Phusion thermostable DNA polymerase. The libraries were quantified using Agilent Bioanalyser chip and Kapa Illumina SYBR Fast qPCR kit. Details of libraries can be found in Table S1B.

Illumina transcriptome libraries (Table S4) were produced using polyadenylated mRNA purified from total RNA using methods previously described except size selection, which was either as described or using the Caliper LabChip XT.

Genome and transcriptome libraries were denatured with 0.1 M sodium hydroxide and diluted to 6 pM in a hybridisation buffer to allow the template strands to hybridise to adapters attached to the flowcell surface. Cluster amplification was performed on the Illumina cluster station or cBOT using the V4 cluster generation kit following the manufacturer’s protocol and then a SYBRGreen QC was performed to measure cluster density and determine whether to pass or fail the flowcell for sequencing, followed by linearization, blocking and hybridization of the R1 sequencing primer. The hybridized flow cells were loaded onto the Illumina Genome Analyser IIX for 76 or 100 cycles of sequencing-by-synthesis using the V4 or V5 SBS sequencing kit then, in situ, the linearization, blocking and hybridization step was repeated to regenerate clusters, release the second strand for sequencing and to hybridise the R2 sequencing primer followed by another 76 or 100 cycles of sequencing to produce paired end reads. These steps were performed using proprietary reagents according to the manufacturer's recommended protocol (<https://icom.illumina.com/>). Data were analysed from the Illumina Genome Analyser IIx or HiSeq sequencing machines using the RTA1.6 or RTA1.8 analysis pipelines.

**3. Sequence Assembly**

We assembled a draft sequence of the *G. pallida* genome based on data from a mixture of sequencing technologies (Sanger capillary sequencing to 0.6-fold coverage, Roche 454FLX to 54-fold coverage and Illumina to 90-fold coverage; see Table S1). Reads from each technology were initially assembled independently using algorithms most appropriate to each technology. 454 data from non-whole genome amplified samples was assembled with version 6.1 of the Celera assembler , with the *mer* overlapper and a kmer length of 27, and parameters *utgErrorRate*=0.04, *utgErrorLimit*=2.5, *ovlErrorRate*=0.06, *cnsErrorRate*=0.1, *cgwErrorRate*=0.1. This produced an assembly with contigs of 95.5Mb and an N50 of 3.2kb that was treated as the master assembly, which contigs from other assemblies were used to improve. Assembly of Illumina reads used Abyss v1.2.7 with a kmer of 55 and requiring 10 read pairs to build a contig, and other settings as default to produce a set of contigs. For assembly of amplified 454 data, the v2.5 Newbler assembler performed better, with an assembly with flags *–het –large –rip* producing a set of contigs with total length 169Mb and N50 1,934bp. Capillary data was assembled with Phusion v2.1 . Following the scheme shown in Figure S1, at each ‘contigs merged’ step, a Perl script – GARM – was used to merge contigs where contigs from the two assemblies had unique overlaps of at least 200bp with at least 99% identity. GARM uses nucmer to identify potential overlaps that are then filtered to identify unique and unambiguous overlaps, that are then used to extend and even join contigs within scaffolds using the overlap-layout-consensus algorithm implemented in the AMOS package . The GARM contig merging script is available from http://garm-meta-assem.sourceforge.net and described in additional detail elsewhere . In each step, this merging was used to extend the contigs from the left-hand input in the diagram, so that merged contigs and anything from this left-hand input that was not merged were kept following this step. Unmerged material from the RHS assembly at each step was discarded to avoid inflating the assembly with divergent haplotypes or additional contamination. Our complete assembly is thus based on the 454 non-WGA material, with contigs improved by input with the other sequence data. Because of concerns about the WGA process and the relatively low depth of capillary sequence data compared to that of other technologies, the final merging (of capillary data) was used on scaffolds from the previous merge, so that contigs could only be joined or extended where that was consistent with previous scaffolding information. Following these merging steps, we built scaffolds based on the Illumina data and non-WGA 454 long-insert libraries. We scaffolded using the 300bp insert Illumina libraries first, then a 1kb insert Illumina library (used only in the scaffolding step), then 3, 8 and 20kb 454 libraries in order using SSPACE v1 ,using 9 runs for each library with the number of links between contigs required being reduced iteratively (60,30,20,10,10,7,7,5,5,5) to allow strong scaffolding links to form before weaker evidence is considered, an approach that extensive experimentation suggested provided robust and sensitive scaffolding.

The assembly was cleaned in two steps – firstly, before gene model prediction, we removed 1,054 supercontigs that had BLASTX hits with E<10^-5^ only to bacterial sequence data in the nr database and to which no RNAseq reads mapped (the poly-A selection step in the RNAseq protocol means that no bacterial transcripts should be present). This produced an assembly of 132 Mb in 9,196 scaffolds with a scaffold N50 of 113 kb. After gene model prediction (see details below), further removal of scaffolds involved removing scaffolds with high GC that have no gene models, and scaffolds that have no gene models with blastp (E < 10^-5^) hits to animal sequences, but do have hits to bacterial, plant or environmental sequences (divisions BCT, PLN and ENV) in the Genbank nr database. Figure S2 shows that this approach removed mostly small scaffolds (2,054 scaffolds, total length 7.1 Mb). A small number of additional scaffolds (284, total length 496kb) were removed as putatively haplotypic scaffolds that were contained within larger scaffolds with 99% identity at the nucleotide level. This produced the final assembly described here. Assembly completeness statistics are shown in Table S2.

To assess the level of polymorphism in our sequencing libraries, we mapped the four illumina libraries to the final assemblies with SMALT (parameters –k 13 –s 1 –x –y 0.85) then called variants using samtools mpileup, followed by filtering with vcfutlls.pl with default parameters except ‘-d 5 -D 70’. This identified a total of 953,841 SNP variants and 139,639 small indels on the 77,985,583 sites at which variants were called (passing the coverage depth thresholds and sufficiently distant from gaps), giving a SNP density 1.22%. This approach is likely to underestimate the true polymorphism level, as these software are designed to call heterozygous sites in diploid organisms, rather than variants segregating in a large population of individuals.

**4. Gene prediction and annotation**

Transcriptome reads were mapped against the genome using TopHat v 2.0.6 with default options except that --mate-std-dev 20 -i 10 -I 30000 and mate inner distance (-r) set to the mean for each RNAseq library. A reference dataset of 407 manually curated *G. pallida* protein-coding genes was generated using evidence from CEGMA (version 2.4) predictions , the RNA-seq mapping and BLAST hits against nematode proteins from Genbank. These were used to train Augustus v2.5.5 , with a predicted sensitivity of 96% and specificity of 94% for nucleotides in coding regions, 89% and 84% for correctly predicting the entire coding sequences of exons and 54% and 46% for entire genes. Final gene prediction was performed by Augustus using parameters from this training set and evidence from introns predicted by cufflinks v.0.9.1 using a combination of all the RNAseq mapping described above.

Functional annotation information was obtained using Interproscan v4.5 and by obtaining product names from BLAST hits to the Genbank nr database using a custom perl script. Gene Ontology terms were annotated via InterPro2GO, Blast2GO , and from the curated *C. elegans* annotation in Wormbase by assigning GO terms shared by all *C. elegans* genes in a gene family to any *G. pallida* genes in the family. In addition to the InterProScan results, signal peptides were predicted using SignalP v3.0 . For particular functional categories of genes of particular relevance to understanding *G. pallida* biology, this primary *in-silico* annotation was supplemented by both manual annotation and further bioinformatic analysis using a range of different techniques focused on particular biological topics, described in Section 8 below. Prediction of tRNA genes used tRNAscanSE v1.2.3 and rRNA using rnammer v1.2 .

Spliced-leader reads were identified by using BLAST to compare RNA-seq reads against a database of the *G. rostochiensis* SL sequences previously identified , accepting perfect matches to at least 11 bp of an SL sequence in the expected position at the end of a read. Because of the high sequence similarity between SL sequences within each SL type, this approach can only classify reads to each SL type, rather than specific SL sequences. Genes were called as being trans-spliced with a particular SL type if at least 5 reads for a particular SL, or the mates of those reads were found to map uniquely either within the gene or within 200bp of the start codon, or if an upstream gene was within that distance, within the intergenic region upstream of the gene.

**5. Comparative genomics analysis**

We used two complementary approaches to compare the predicted proteome of *G. pallida* with that of other nematodes. The OMA algorithm identified one-one orthologs across species (called one-one orthology groups) and OrthoMCL provided a wider view of gene family evolution (called gene families). In both analyses, we included the predicted proteins of *G. pallida*, those for the three other published plant parasitic nematodes *(M. hapla, M. incognita, B. xylophilus),* together with predicted proteins from *C. elegans* and used the animal parasitic filarial nematode *B. malayi* as an outgroup. The phylogenetic tree in Figure 2 was estimated based on the concatenated alignment of 432 protein-coding genes that were inferred as single-copy orthologs across all species using the OMA orthology groups. Alignments for each gene were generated using mafft v6.857 with –auto, and cleaned with glbocks v.0.91b using the best fitting amino acid substitution model (WAG+F+I+G) under AIC and the default search strategy of RAxML v.7.2.8 . Birth and death of gene families was inferred under Dollo parsimony using the Dollop program from v3.69 of the Phylip package .

**6. RNA-seq analysis**

The numbers of RNA-seq reads per gene model were counted using custom-made scripts building on BEDtools v2.12 and a gff file of the genome annotation, using the read mapping described above. Description of gene expression levels and counts was based on mean RPKM values across the duplicate samples for each life stage. We used two formal statistical approaches to investigate how gene expression varies during the life cycle of *G. pallida*. Pairwise tests using the default normalization, and dispersion estimation procedures for the negative binomial test implemented in DESeq v1.8.1 were used to identify genes showing significantly different expression between parts of the life cycle. Genes with false discovery rate less than or equal to 1e-5 were retained. Inspection of expression level data suggested that the difference in expression between samples for some life stages was greater than that between some of the stages we investigated (Figure S8). We therefore adopted a conservative analysis approach by testing for significant differences only between specific sample groups: between egg and pre-infective J2 larvae, between J2 and early parasitic stages (7 and 14 dpi samples), between early parasitic stages and adult females (21, 28 and 35 dpi samples), between adult males and pre-infective J2 larvae, and between adult males and early parasitic stage samples. GO terms significantly enriched (p < 0.01) in the set of differentially expressed genes from each comparison were identified using the “weight01” algorithm of TopGO v 2.8.0 . Expression data was drawn using Circos-0.62 . Model-based clustering of gene expression profiles across the life cycle was used to identify groups of genes with similar patterns of expression. Differentially expressed genes were clustered using MBCluster.seq (unpublished; http://cran.r-project.org/web/packages/MBCluster.Seq/index.html) with 75 clusters. For Figures 3B and 4A clusters were then ordered based on the stage with highest mean expression in that cluster.

**7. Identifying and annotating repeats**

Transposable elements (TEs) in the assembly were identified using two approaches. The first stage consisted of *de novo* identification of repeat families in the assembly based on signatures of transposable elements and assuming fragments of TEs are present throughout the genome. Long terminal repeat (LTR) retrotransposons were identified using LTRharvest which searches for two near-identical copies of an LTR flanked by target site duplications that are close to each other. We also used RepeatModeler (http://www.repeatmasker.org/RepeatModeler.html) which aims to construct repeat consensus from two *de novo* detection programs (RepeatScout and RECON). Repeats present at less than 10 copies in the genome or that were less than 100 bp were excluded from further analysis. The second approach used homology searching of the assembly sequence against curated TEs using TransposonPSI (http://transposonpsi.sourceforge.net/). UCLUST was used to cluster the candidate sequences (with 80% identity) and create a non-redundant library of repeat consensus sequences. The annotation of repeat candidates involved a search against RepBase and NCBI non-redundant library. Some of these candidates that have some annotations available from program output (for example, from TransposonPSI) were further checked this way. Manual curation of the candidates was carried out to determine coding regions on intact TEs that are potentially active. RepeatMasker (v3.2.8) was used to calculate the distribution of each repeat and its abundance. Custom perl scripts were used to choose the best match from overlapping matches in RepeatMasker output to avoid calculating the same region twice or more when considering repeat content of the genome.

**8. Annotation and analysis of functional gene categories**

**CAZymes.** The CAZymes Analysis Toolkit (CAT) was used to identify putative carbohydrate active enzymes (CAZymes) using a predefined CAZy database on the *G.pallida* predicted protein set V1.0. Expansin-like genes were detected by BLAST searching using known nematode expansin proteins as queries. Putative CAZymes and expansins were manually annotated using a combination of BLASTp (vs nr database), NCBI's Conserved Domain Database service and InterProScan to determine to presence of the catalytic domains.

**Identification of effectors.** *G. pallida* orthologs of effectors identified in other plant parasitic nematodes were identified by BLAST searching of the *G. pallida* genome and predicted protein set. Cut off values of 10e-5 with a match across more than 50% of the query sequence were used for initial screens. Novel effectors were identified in a two stage process. All potentially secreted proteins from *G. pallida* were identified on the basis of the presence of a Signal peptide and the absence of a transmembrane domain (TMHMM - http://www.cbs.dtu.dk/services/TMHMM-2.0/) in a bespoke pipeline run through the JHI installation of Galaxy. Secreted proteins that were significantly up-regulated in J2 versus eggs or in 7 dpi parasitic nematodes versus J2 were then selected. These sequences were BLAST searched against the nr database and those that had functions unrelated to parasitism (*e.g.* collagens, digestive proteinases) but which came through this screen were manually removed.

**Identification of genes acquired by horizontal gene transfer (HGT).** The predicted *G. pallida* proteins were searched against the nr database with an e-value cut off of 10^-5^. Any proteins with a top match against a nematode protein, or that had no matches in the database were then discarded. The remaining matches were inspected manually and potential HGT events, in which the top match was to a bacteria or fungus, were identified. These protein sequences were examined for the presence of a signal peptide as described above.

**Neurotransmitter biosynthesis and metabolism.** *C. elegans* proteins involved in the synthesis, transport or catabolism of the neurotransmitters acetylcholine (ACh), serotonin (5HT), dopamine (DA), tyramine (TA), octopamine (OA), glutamate (Glu) and gamma-aminobutyric acid (GABA) as described by were used in BLASTP searches to identify putative orthologs amongst the predicted *G. pallida* proteins. Reciprocal BLAST searches of the *C. elegans* protein database on Wormbase (version WS232) using the predicted *G. pallida* proteins were then used to confirm the identity of orthologous genes. In cases where a *G. pallida* orthologue was not identified amongst the predicted proteins, tBLASTn searches of the scaffold sequences were carried out. Automated prediction errors leading to fused or split gene predictions or truncated proteins were corrected manually using alignment based-evidence from the BLAST searches described and analysis of transcript coverage plots mapped to the genome assembly on a GBrowse platform.

**Neuropeptide genes.** Neuropeptide genes encoding FLPs (FMRFamide-like peptides) and NLPs (neuropeptide-like proteins) were identified using BLASTP searches of the predicted *G. pallida* proteins and tBLASTn searches of the genome scaffolds. Search strings used initially were each predicted *C. elegans* FLP and NLP, plus those additional peptides identified from *Meloidogyne incognita* and *Bursaphelenchus xylophilus* . Additional searches were carried out using concatenated strings of the mature peptides encoded by each *C. elegans* or plant parasitic nematode ortholog, including the dibasic amino acid cleavage sites. All putative *flp* and *nlp* orthologs with an E-value threshold of ≤1e-3 were manually assessed to confirm the presence of the conserved mature peptide motifs and appropriately located cleavage sites. Automated prediction errors leading to fused or split gene predictions or truncated proteins were corrected manually using alignment based-evidence from the BLAST searches described.

**Neurotransmitter receptors.** Neurotransmitter function relies on the activation of specific receptors. The known *C. elegans* receptors for acetylcholine, dopamine, tyramine, octopamine, glutamate and GABA were identified in WormAtlas and used in BLASTP searches of the predicted *G. pallida* proteins. All primary BLASTP hits with an E-value threshold of ≤1e-10 were analysed further for presence of appropriate conserved domains using RPS-BLAST to search the NCBI Conserved Domain Database. Putative *G. pallida* receptor sequences were used in reciprocal BLAST searches of the *C. elegans* protein database on Wormbase to assign orthologous genes where possible. For those *C. elegans* genes where an ortholog was not identified amongst the *G. pallida* predicted proteins, tBLASTn searches of the scaffold sequences were carried out. Additional orphan ligand-gated ion channels (LGICs) were identified using the results of InterProScan of all predicted *G. pallida* proteins to find those containing the InterPro domain IPR006202 (neurotransmitter-gated ion-channel ligand-binding).

**RNAi pathway genes.** Seventy-seven *C. elegans* proteins with roles in small RNA biosynthesis, dsRNA uptake, the RNA-induced silencing complex (RISC), RNAi inhibition or as nuclear effectors have previously been identified as being involved in core aspects of the RNAi pathway . The sequences of these transcripts were obtained from NCBI and used in BLAST searches of the *G. pallida* nucleotide dataset for predicted genes. All BLAST hits with an E-value threshold of ≤1e-20 were manually analysed for accuracy of automated gene prediction, corrected if necessary and the corresponding *G. pallida* predicted proteins subjected to reciprocal BLASTP searches against the *C. elegans* protein database to assign orthologs where possible. Protein domains were identified using RPS-BLAST to search the NCBI Conserved Domain Database.

**Antioxidants.** Hidden Markov Models (HMMs) were downloaded from http://pfam.sanger.ac.uk/ for catalase (PF00199), glutathione peroxidase (PF00255), glutathione synthetase (PF03199 and PF03917), peroxiredoxin (PF00578) and superoxide dismutase (PF00080, PF00081 and PF02777). Searches were performed against the predicted *G. pallida* protein dataset using HMMER (downloaded from http://hmmer.janelia.org/). In addition, BLAST searches were carried out with full length *C. elegans* nucleotide sequences from each family against the *G. pallida* nucleotide dataset in order to identify predicted genes with incomplete domains. The *C. elegans* transcript sequence for the only copper chaperone gene (*cuc-1*) was obtained from NCBI and BLAST searches were performed against the predicted *G. pallida* nucleotide dataset. All BLAST hits were manually analysed for accuracy of automated gene prediction, corrected if necessary and subjected to reciprocal BLAST searches against the *C. elegans* protein database to assign orthologs where possible.

**Cellular metabolism and excretion.** HMMs were downloaded from http://pfam.sanger.ac.uk/ for cytochrome P450 (PF00067), glucuronosyl transferase (PF00201), glutathione transferase (PF00043 and PF02798) and membrane transporters (PF00005 and PF00664). Searches were performed against the predicted *G. pallida* protein dataset using HMMER (downloaded from http://hmmer.janelia.org/). In addition, BLAST searches were carried out with full length *C. elegans* nucleotide sequences from each family against the *G. pallida* nucleotide dataset in order to identify predicted genes with incomplete domains. All BLAST hits were manually analysed for accuracy of automated gene prediction, corrected if necessary and subjected to reciprocal BLAST searches against the *C. elegans* protein database to assign orthologs where possible.

**Immune Response.** *C. elegans* transcript sequences for proteins belonging to the TGF-beta signalling pathway, ERK-MAPK signalling pathway, P39 MAPK signalling pathway and Toll signalling pathways as well as antibacterial and antifungal genes as described by were obtained from NCBI. BLAST searches were performed against the predicted *G. pallida* nucleotide dataset. All BLAST hits were manually analysed for accuracy of automated gene prediction, corrected if necessary and subjected to reciprocal TBLASTX searches against the *C. elegans* protein database to assign orthologs where possible. Protein domains were identified using RPS-BLAST to search the NCBI Conserved Domain Database.

**Nuclear hormone receptors.** Hidden Markov Models were downloaded from http://pfam.sanger.ac.uk/ for both ligand binding domains (PF00104) and DNA binding domains (PF00105). Searches were performed against the predicted *G. pallida* protein dataset using HMMER (downloaded from http://hmmer.janelia.org/). In addition BLAST searches were carried out with full length *C. elegans* nucleotide sequences from each family against the *G. pallida* nucleotide dataset in order to identify predicted genes with incomplete domains. All BLAST hits were manually analysed for accuracy of automated gene prediction, corrected if necessary and subjected to reciprocal BLAST searches against the *C. elegans* protein database to assign orthologs where possible.

**SUPPORTING RESULTS**

**Operons and spliced leaders**

We looked for homologs of the genes from 1,353 *C. elegans* operons that consist of more than one functional gene (451 had more than two genes). 782 have *G.* *pallida* homologs to all genes in the operon, and a total of 982 have 2 or more homologs. While the gene content of *C. elegans* operons is largely conserved, there is little evidence that these genes are still arranged in operons in *G. pallida*. Just 99 (7%) have at least two *G. pallida* copies adjacent in the genome, while 883 have no adjacent homologs. The fragmentary nature of a draft genome may have biased this downwards: 371 operons could not show adjacency because one gene is at a scaffold end. The low conservation of operons in *G. pallida* could represent either a general loss of operon-type organization in this species, or extensive re-organisation of operons. The transcription data confirm that closely neighbouring genes (less than 200 bp apart, reflecting the approximate distances between genes within operons in a range of nematode species ) on the same DNA strand show correlated expression levels, a pattern not shown by other adjacent gene pairs (Figure S4).

Genome analysis of other plant parasitic nematodes has found only SL1-type sequences , but more recently SL2-like sequences have been identified in *Aphelenchus avenae*, a clade IV nematode only distantly related to *Globodera* , and both SL2-like and more diverse SL sequences are found within clade I . In addition, there is evidence that a diverse range of 27 different SL sequences are trans-spliced to a single gene in *G. rostochiensis*, with a total of 30 distinct SLs in four classes reported from this species , forming four distinct clusters of similar sequences. To clarify the importance and roles of these different SL types, we mapped identified RNA-seq reads containing sequences similar to the published clusters of *G. rostochiensis* SLs to the genome. We found significant numbers of reads matching all but 4 of the published sequences, suggesting that there are at least 26 different SL sequences in *G. pallida* (see Table S6). A total of 7,569 genes can be identified as being trans-spliced from the *G. pallida* data, with most (7,185) spliced to cluster SL1 and fewer showing evidence of the involvement of sequences belonging to the other SL clusters (1,496 SL2; 2,647 SL3 and 87 SL4). Many genes appear to be trans-spliced promiscuously – while 4,393 genes were uniquely trans-spliced with SL1-type sequences, only 323 genes were uniquely spliced with any of the other SL types, so that almost all genes that receive non-SL1 sequences are also spliced to SL1. The pattern of SL usage for genes in the few gene pairs that are conserved in order and orientation from *C. elegans* operons was similar to that across the genome, if slightly enriched for non-SL1 types (134; 45; 64; 3 genes spliced with the SL1-SL4 classes respectively). There was also no clear pattern in the use of the different SLs with distance between genes, except that SL2-spliced genes tend to have a slightly closer upstream neighbor, following the (much stronger) trend in *C. elegans* . Examining SL usage in 109 adjacent gene pairs that are less than 200 bp apart on the same strand, and show highly correlated expression levels (R^2^ > 0.85), and so form potential operons in *G. pallida*, we found no significant relationship between SL usage and the position of genes in the potential operon.

**Conservation of the RNAi pathway in *G. pallida***

RNA interference (RNAi), the process by which double stranded RNA (dsRNA) initiates homology-dependent transcriptional gene silencing, was first described for *C. elegans* where it has become an invaluable gene silencing tool for functional analysis. Since it was first demonstrated that RNAi could be used to silence genes in J2 cyst nematodes dsRNA has been delivered to a range of plant parasitic nematode species both *in vitro*, as a tool for functional genomics, and *in planta* as a strategy for transgenic control. However, the molecular details of the pathways involved have not been elucidated and inconsistent levels of gene silencing have been reported, although the technique seems more reliable than for many animal parasitic species . For nematode species in which RNAi is less effective than in *C. elegans*, particular genes involved in the RNAi pathway may be absent or not well conserved.

A recent study identified 77 *C. elegans* proteins involved in the five key stages of the RNAi pathway: small RNA biosynthesis, dsRNA uptake and spreading, Argonautes (AGOs) and RNA-induced silencing complex (RISC) components, RNAi inhibitors and nuclear effectors . Like other parasitic nematodes studied, *G. pallida* contains genes involved in most aspects of the RNAi pathway characterised in *C. elegans,* but has fewer genes overall and is particularly deficient in those proteins responsible for uptake of dsRNA and systemic RNAi effects (Table S15). Orthologs encoding many of the proteins required for siRNA and miRNA processing have been found, including RNase III enzymes (*drsh-1*, *psh-1*, *dcr-1*), RNA helicases (*drh -3*) and exportins (*xpo-1*) as found in other nematodes. However *drh-1*, *rde-4* and *xpo-3* do not appear to be conserved in *G. pallida*, although an ortholog for *drh-1* has been identified in both *M. hapla and M. incognita.* Components of the amplification complex (*ego-1*, *smg-2* and *smg-6*) have also been putatively identified in *G. pallida* with three genes displaying clear homology to the RNA-dependent RNA polymerase (RdRP) *ego-1*. A similar expansion of *ego-1* orthologs was observed in *B. xylophilus* . Similarly to *Meloidogyne* and some other parasitic nematode species no orthologs were found in *G. pallida* for the amplification genes *rrf-1*, *rrf-3*, *smg-5* and *rsd-2*, or the genes involved in uptake of dsRNA and its spreading to surrounding cells; *sid-1*, *sid-2*, and *rsd-6*. Of this latter category, only the well-conserved *rsd-3* gene thought to be involved in the intercellular distribution of dsRNA following uptake was found to be present.

Eleven Argonaute genes appear to be present in *G. pallida*. Both *alg-1* and *R06C7.1* (*wago-1*) are also well conserved in *Meloidogyne* and other parasitic nematode species. As for *B. xylophilus*, there is some expansion of particular AGOs, with two *wago-2*-like AGOs, three *wago-5*-like AGOs and two *wago-11*-like AGOs. The reduced total complement of AGOs in comparison to *C. elegans* is typical of that seen in other parasitic nematodes . Additional components of the RISC complex, including exonucleases and dsRNA-binding proteins, remain poorly characterised in *C. elegans* and only one of these the exonuclease TSN-1 is predicted to be present in *G. pallida*.

Genes encoding only two RNAi inhibitors (*eri-1* and *xrn-2*) are predicted in *G. pallida*, a situation also found in *M*. *incognita*. Of the 15 *C. elegans* genes designated as having putative roles as nuclear RNAi effectors orthologs for five genes (*cid-1*, *gfl-1*, *mes-2*, *ekl-4*, *rha-1*) have been identified in *G. pallida* which are all conserved in *M. incognita*. *G. pallida* appears to have homologues for most of the genes encoding the RNAi pathway which are also present in *Meloidogyne* and other parasitic nematode species. Where homologues appear to be missing in these organisms it is possible that alternative proteins or poorly conserved proteins may facilitate effective uptake and spreading of dsRNA and siRNA in *G. pallid*a as these nematodes do display systemic RNAi following soaking of J2s in dsRNA or siRNA .

**Neurotransmission**

Despite a relatively simple structure, the nematode nervous system is able to service complex and subtle behavioural responses, accomplished by sophisticated signaling with a diverse array of signaling molecules such as neuropeptides and inherent heterogeneity of receptors for classical neurotransmitters. For example, nematode receptors for acetylcholine (ACh) and glutamate are comprised of distinct subunits that can assemble in multiple combinations to provide a high degree of receptor plasticity. Beside its inherent interest, the nematode nervous system is a particular target for chemical control methods, so greater understanding of the available target molecules may help in the rational design of new nematicides.

We confirm the presence of genes responsible for the production and utilization of the neurotransmitters acetylcholine (ACh), serotonin (5HT), dopamine, tyramine, octopamine, glutamate and gamma-aminobutyric acid (GABA), with a very similar complement of genes to *C. elegans*. The similarity extends to the conserved structure of the two key genes involved in the synthesis and vesicular transport of acetylcholine. The *G. pallida* orthologs of *cha-1* and *unc-17*, encoding choline acetyltransferase and a synaptic vesicle ACh transporter respectively, are organised in an operon, with the *cha-1* and *unc-17* transcripts probably derived from alternative splicing of a single precursor RNA. Similarly, most subtypes of neurotransmitter receptors found in *C. elegans* are present in *G. pallida,* but there are differences in the complement of particular types*. G. pallida* has a somewhat smaller repertoire of nicotinic acetylcholine receptors (nAChRs) than *C. elegans*, with a particularly reduced number of ACR-16 class receptors. It does, however, contain members of each of the five distinct groups of nAChRs and again, operon organization of some of these genes (*acr-2* and *acr-3*, *des-2* and *deg-3*) appears conserved. Another intriguing exception is the lack of a clear ortholog for *C. elegans* serotonin receptor SER-1; this has a key role in the regulation of egg-laying in *C. elegans*, through control of the vulval muscle . As all potato cyst nematode eggs are retained inside the female body this role may be redundant in *Globodera* spp. *G. pallida* is also missing both NMDA class subunits, *nmr-1* and *nmr-2* of the ionotropic glutamate receptors , and has only four of the six glutamate-gated chloride channels found in *C. elegans* – these are of particular importance as targets of the anthelminthic avermectin .

Neuropeptides, derived from precursor proteins that are processed to yield short, active amino acid sequences, can act as neurotransmitters but their main role is as modulators of synaptic activity in a range of processes including sensory perception, locomotion, development, egg-laying and dauer formation. More than 100 neuropeptide-encoding genes have been identified in the *C. elegans* genome, corresponding to more than 250 distinct peptides in three classes: the FMRFamide-like peptides (FLPs), the insulin-like peptides (ILPs) and the more diverse group of neuropeptide-like proteins (NLPs). In common with other plant parasitic species for which detailed data is available *, G. pallida* has a reduced complement of *flp* genes compared to *C. elegans* and does harbor a homolog of *flp-30*, one of two genes identified to-date only in *Meloidogyne* spp., but apparently lacks *flp-31*. Uniquely amongst nematodes, two distinct *G. pallida* genes give rise to the FLP-16 peptide, one encoding three copies of the peptide and the other just a single copy. There are also two identical copies of the *flp-6* gene, located approximately 15 kb apart on the same scaffold and 3 genes that each encode peptides similar to FLP11. *G. pallida* also has a greatly reduced complement of *nlp* gene orthologs, with only 10 identified in the *G. pallida* genome assembly, compared with 22 and 17 for *M. incognita* and *B. xylophilus* and 37 *C. elegans* genes. *C. elegans* *nlp-24-33* encode putative anti-microbial peptides with likely roles in non-neuronal signalling .

**SUPPORTING FIGURES**

**
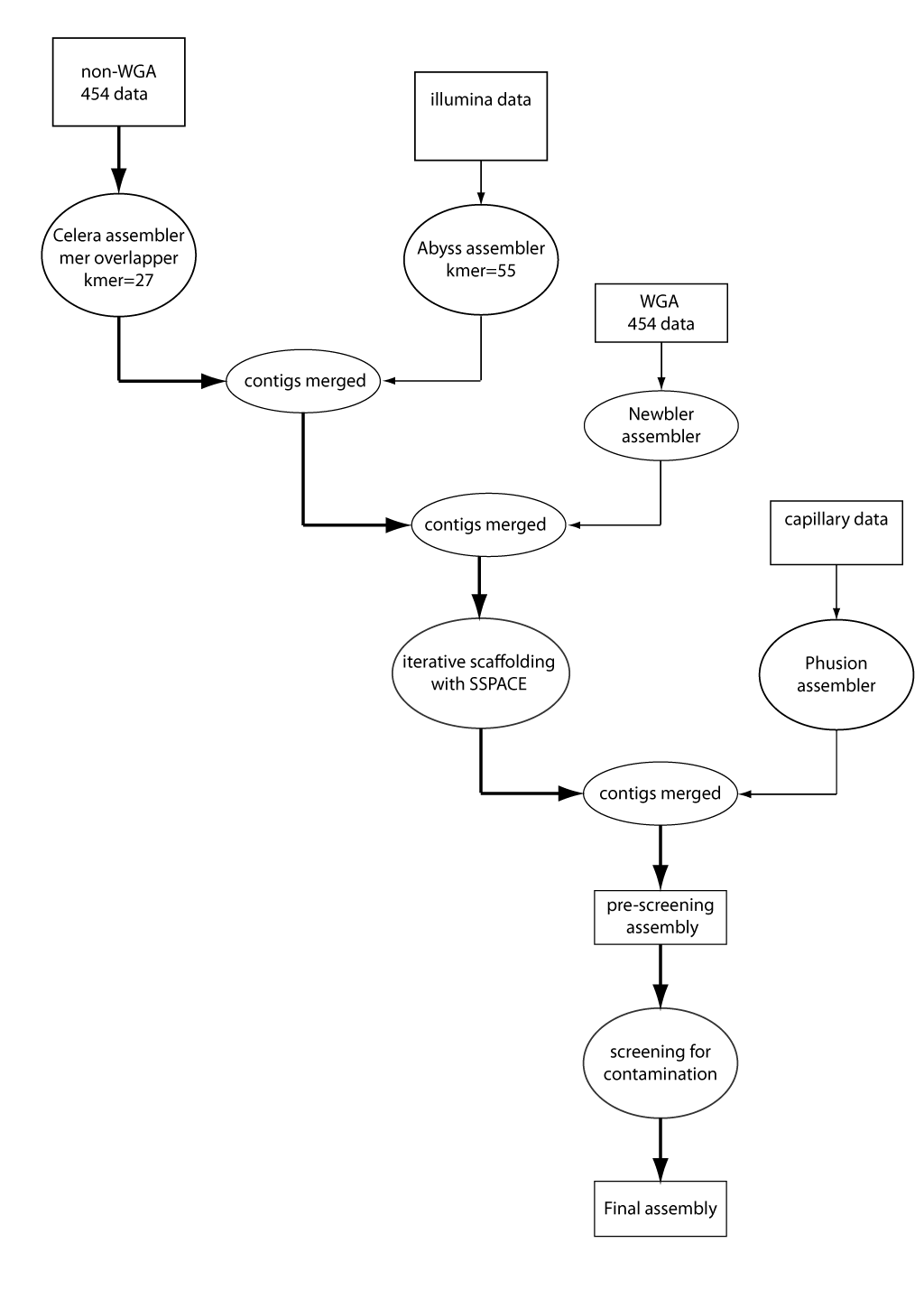
**

**Figure S1. Flowchart of *Globodera pallida* assembly process.**

Bold arrows indicate the principle contributions to the final assembly – other data was used only to extend and join contigs from this path. See Supporting Methods for full details.

**
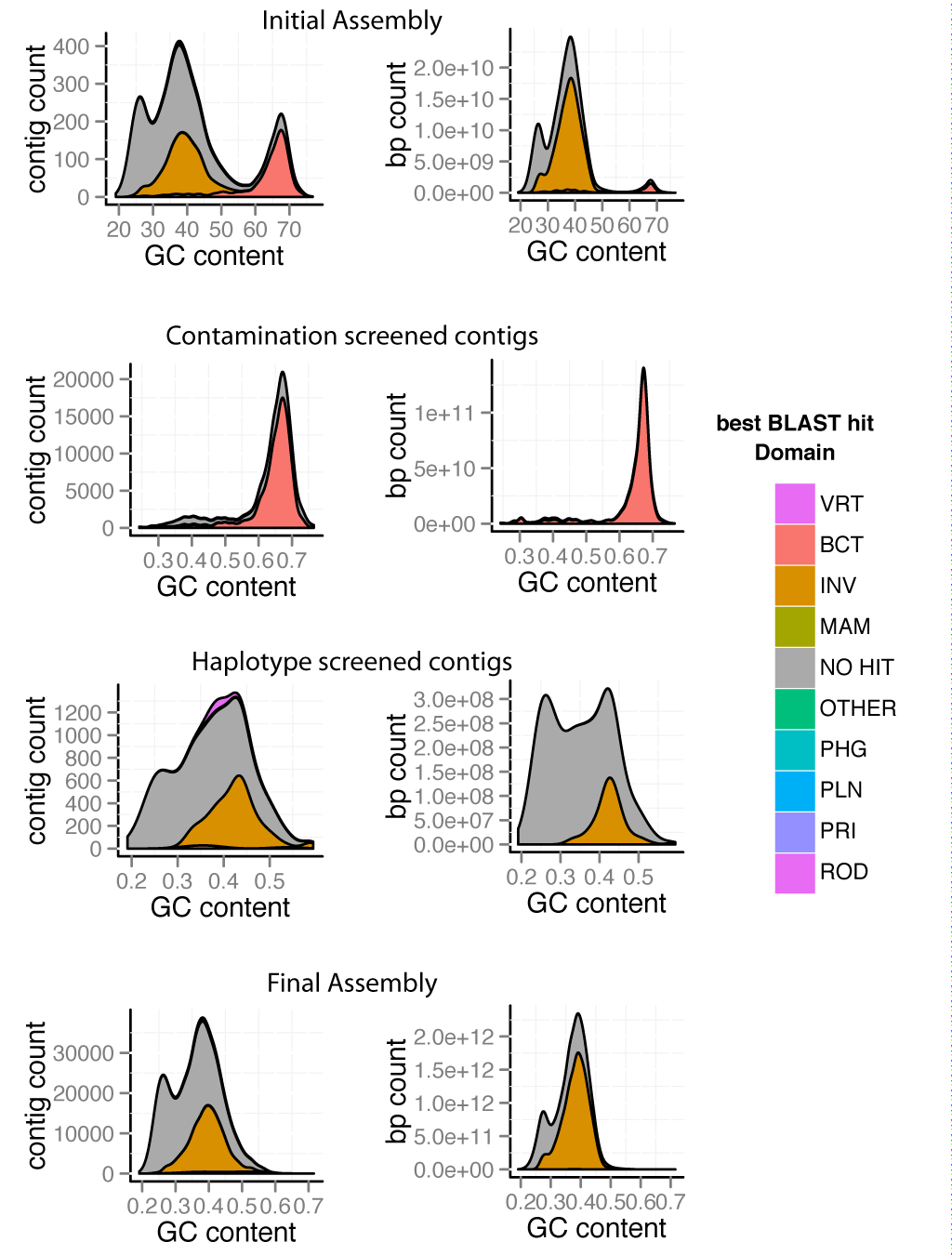
**

**Figure S2. GC content and taxonomic distribution of contigs in *Globodera pallida* assembly at different stages of contamination filtering.** Each figure shows the distribution of GC content for contigs with best BLAST hits to different Genbank taxonomy domains during the process of removing putatively contaminant contigs. Figures show distribution of contigs (left column) and of base pairs (right column). Bacterial contaminants were largely small, high-GC contigs.

**
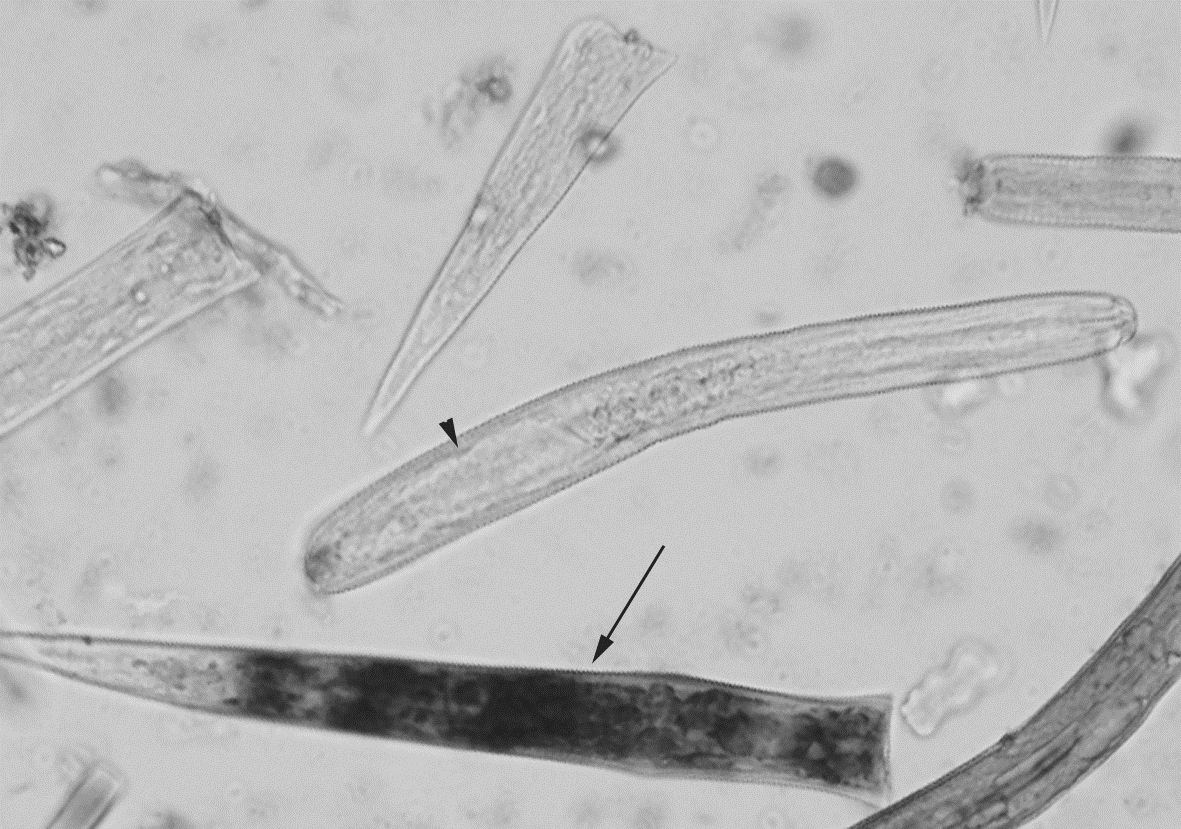
**

**Figure S3. Intestinal expression of one member of the *Globodera pallida* “dorsal gland-specific” gene family.**

*In situ* hybridization showing that expression of one member of the highly expanded *G. pallida* "dorsal gland specific" gene family is restricted to the digestive system (dark staining - arrow) in 2nd-stage juveniles. No evidence of expression in the dorsal gland cells (arrowhead) is observed. *In situ* hybridizations were performed as previously described .

**
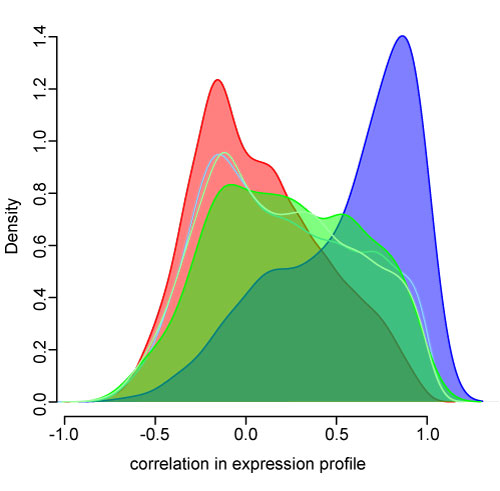
**

**Figure S4. Frequency distribution of expression correlation between pairs of *Globodera pallida* genes.** Closely-spaced (< 200bp apart) pairs of adjacent genes on the same coding strand (dark blue, filled density plot; mean R^2^=0.56) are highly skewed towards highly correlated expression levels across RNA-seq samples than either more distant adjacent gene pairs on the same strand (light blue curve; mean R^2^=0.20), or either close- or distant adjacent gene pairs on different strands (filled, dark green and open light green curves; mean R^2^ 0.23 and 0.19 respectively), or 10,000 randomly chosen pairs of *G. pallida* genes (red curve; mean R^2^ 0.07).

**
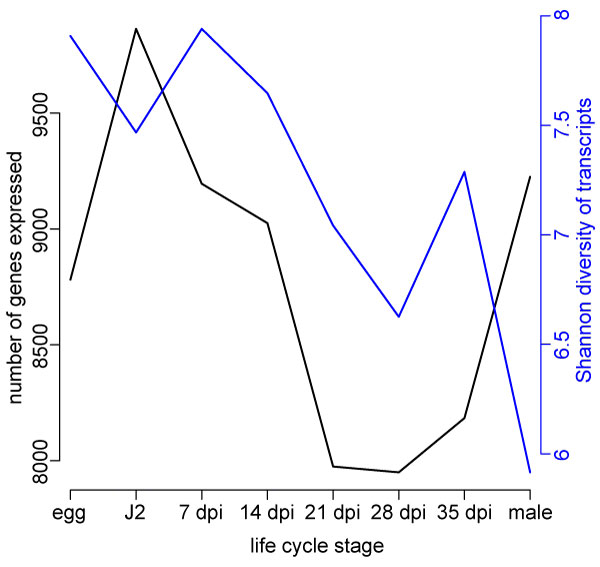
**

**Figure S5. Global variation in expression levels across *Globodera pallida* lifecycle stages**. Black line shows the total number of genes expressed above intragenic background level for each lifecycle stage. Blue shows the Shannon’s diversity index for transcripts at each stage, describing the complexity of the transcript pool.

**
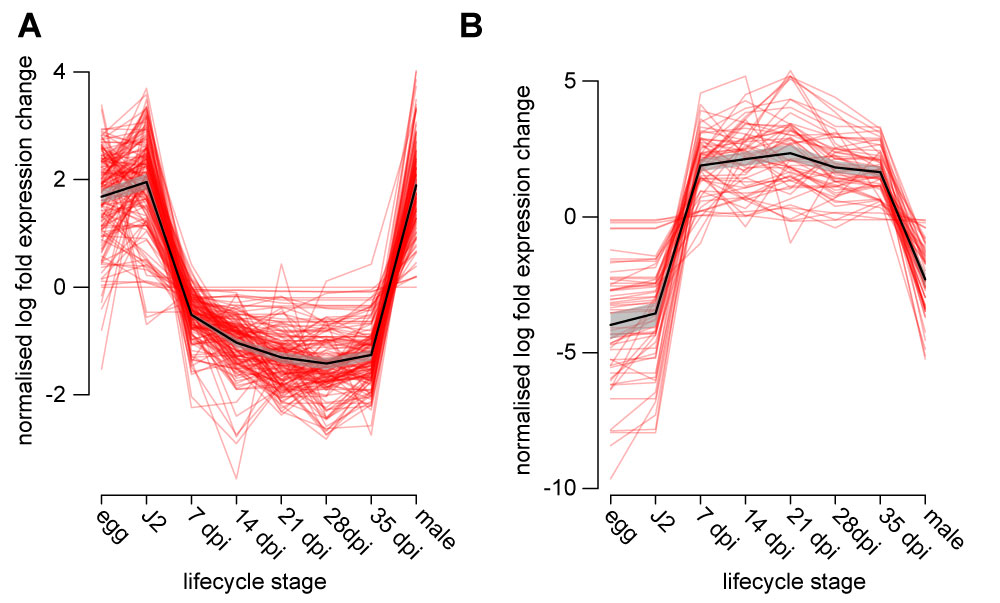
**

**Figure S6.** **Clustering of genes by expression dynamics.** (A) A cluster of 154 genes uniquely up-regulated in J2 and adult males enriched in genes involved in neuromuscular function, specifically potassium ion transport, G-protein coupled receptor signaling, glutamine metabolic process and neurotransmitter:sodium symporter activity, (B) A cluster of 59 genes upregulated in parasitic (feeding) stage nematodes which could reflect the fact that these life stages are the only stages that feed and that undergo moulting. This set is enriched in genes involved in proteolysis, structural constituent of cuticle and metalloendopeptidase activity. Red lines show expression levels of individual genes, black lines are the mean expression for each cluster, grey shading indicates 99% exponential confidence interval for the mean. Note that the clustering approach groups genes with similar patterns, but potentially very different magnitudes, of variation in expression across stages.


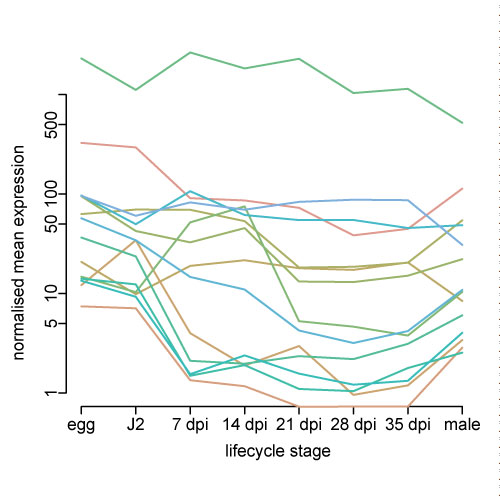


**Figure S7. Expression levels of diapause-related genes.** Each line shows DESeq normalized expression levels for each lifecycle stage for one of the diapause-related genes listed in Table S14.


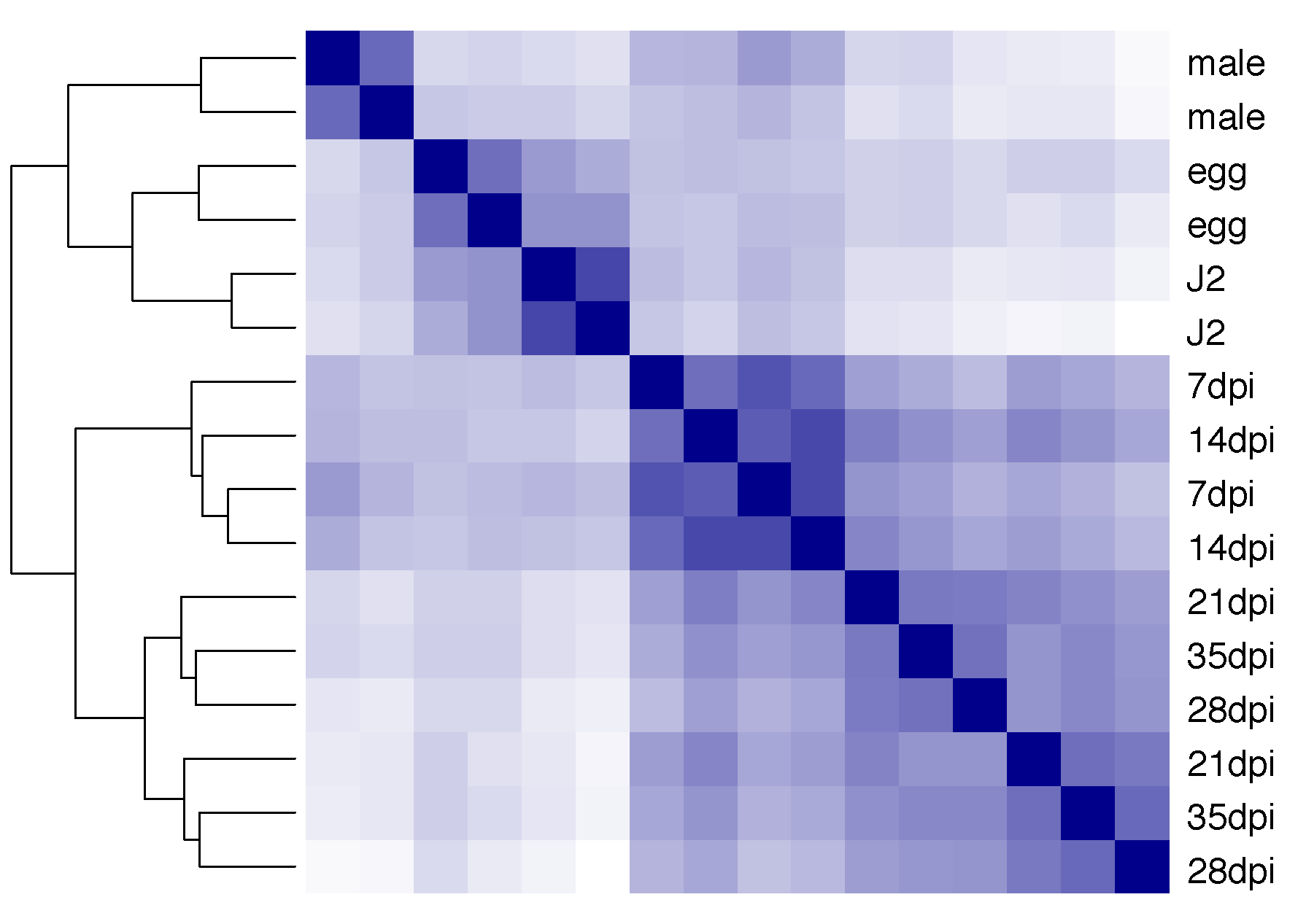


**Figure S8**. **Heatmap showing similarity of different transcriptome libraries.** Euclidean distance between samples based on the variance stabilized data from DESeq clustered using the heatmap function in R, with darker blue colour indicating closer correlation of expression levels between RNAseq libraries. This overview of the lifecycle suggests that males, eggs and J2 stages have distinct transcriptomes, furthermore early post-infective stages (7dpi and 14dpi) are distinct from later infective stages (21, 28, 35dpi). However transcriptomes do not vary much within early or late post-infective stages.

**SUPPORTING TABLES**

**Table S1. Genomic sequencing libraries included in the assembly.** Data are shown for (A) Capillary sequencing of clone libraries and (B) 454 and Illumina sequencing libraries. Statistics for 454 and capillary sequencing (Sanger) reads are all post-trimming of low quality bases. *all reads in an Illumina sequencing run are the same length, before trimming/clipping, other technologies give variable read lengths.

**(A**)

| **(internal) Library ID** | **Sequencing technology** | **Number of sequencing reads** | **Total length of reads (bp)** | **Mean read length (bp)** | **Target insert length** | **Whole genome amplified material** | **Vector** | **Trace archive SEQ_LIB_ID** |
| --- | --- | --- | --- | --- | --- | --- | --- | --- |
| 124544 | Sanger | 723 | 477,223 | 660.1 | 2-3kb | Y | pOTW12 | 124544 |
| 124545 | Sanger | 468 | 256,030 | 547.1 | 3-4kb | Y | pOTW12 | 124545 |
| 124546 | Sanger | 361 | 211,775 | 586.6 | 4-5kb | Y | pOTW12 | 124546 |
| 124547 | Sanger | 417 | 258,954 | 621.0 | 5-6kb | Y | pOTW12 | 124547 |
| 124548 | Sanger | 85,521 | 44,891,135 | 524.9 | 6-9kb | Y | pMAQ1Sac_BstXI | 124548 |
| 124549 | Sanger | 36,708 | 20,277,233 | 552.4 | 9-12kb | Y | pMAQ1Sac_BstXI | 124549 |
| 130307 | Sanger | 17,461 | 6,718,583 | 384.8 | 38-42kb | Y | pCC1Fos | 130307 |
| 132888 | Sanger | 2,411 | 979,339 | 406.2 | 38-42kb | Y | pCC1Fos | 132888 |

**(B**)

| **(internal) Library ID** | **Sequencing technology** | **Number of sequencing reads** | **Total length of reads (bp)** | **Mean read length (bp)** | **% paired in sequencing** | **Target insert length (UP for unpaired ‘shotgun’ sequencing reads** | **Whole genome amplified material** | **Study accession number** | **Sample accession number** |
| --- | --- | --- | --- | --- | --- | --- | --- | --- | --- |
| 2009_03_18_FLX3_Ti | 454FLX Ti | 659,699 | 247,828,891 | 375.67 | UP | UP | Y | ERP000297 | ERS002003 |
| 2009_04_06_FLX3_Ti | 454FLX Ti | 591,596 | 216,258,478 | 365.55 | UP | UP | Y | ERP000297 | ERS002003 |
| 2009_07_20_FLX3_Ti | 454FLX Ti | 1,248,815 | 468,657,577 | 375.28 | UP | UP | Y | ERP000297 | ERS002003 |
| 2010_01_05_FLX3_Ti | 454FLX Ti | 714,708 | 179,962,496 | 251.8 | UP | UP | Y | ERP000297 | ERS002003 |
| 2010_01_13_FLX1_Ti | 454FLX Ti | 982,973 | 330,195,294 | 335.91 | UP | UP | Y | ERP000297 | ERS002003 |
| 2010_02_17_FLX3_Ti | 454FLX Ti | 1,082,559 | 419,850,932 | 387.83 | UP | UP | Y | ERP000297 | ERS002003 |
| 2009_08_20_FLX3_Ti | 454FLX Ti | 1,284,493 | 213,592,326 | 166.29 | 49.4 | 3kb | Y | ERP000297 | ERS002003 |
| 2010_01_06_FLX3_Ti | 454FLX Ti | 828,119 | 113,634,570 | 137.22 | 31.3 | 3kb | Y | ERP000297 | ERS002003 |
| 2010_01_08_FLX3_Ti | 454FLX Ti | 713,812 | 93,616,079 | 131.15 | 28.5 | 3kb | Y | ERP000297 | ERS002003 |
| 2010_02_19_FLX3_Ti | 454FLX Ti | 1,560,509 | 285424653 | 182.9 | 62.7 | 3kb | Y | ERP000297 | ERS002003 |
| 2009_06_23_FLX3_Ti | 454FLX Ti | 152,615 | 50,454,581 | 330.6 | UP | UP | N | ERP000297 | ERS196663 |
| 2009_07_28_FLX3_Ti | 454FLX Ti | 1,003,621 | 367,468,407 | 366.14 | UP | UP | N | ERP000297 | ERS196663 |
| 2010_03_26_FLX3_Ti | 454FLX Ti | 928,265 | 347,174,136 | 374.01 | UP | UP | N | ERP000297 | ERS196663 |
| 2010_04_16_FLX3_Ti | 454FLX Ti | 835,42 | 303,855,549 | 363.75 | UP | UP | N | ERP000297 | ERS196663 |
| 2010_05_26_FLX3_Ti | 454FLX Ti | 563,343 | 196,576,588 | 348.95 | UP | UP | N | ERP000297 | ERS196663 |
| 2009_08_21_FLX3_Ti | 454FLX Ti | 1,497,240 | 264,319,955 | 176.54 | 60.6 | 3kb | N | ERP000297 | ERS002003 |
| 2010_01_21_FLX1_Ti | 454FLX Ti | 713,522 | 185,741,335 | 260.32 | UP | UP | Y | ERP000297 | ERS196662 |
| 2010_02_04_FLX3_Ti | 454FLX Ti | 1,157,258 | 435,500,702 | 376.32 | UP | UP | Y | ERP000297 | ERS196662 |
| 2010_02_12_FLX1_Ti | 454FLX Ti | 680,510 | 104,761,570 | 153.95 | UP | UP | Y | ERP000297 | ERS196662 |
| 2010_02_12_FLX3_Ti | 454FLX Ti | 1,016,559 | 325,171,083 | 319.87 | UP | UP | Y | ERP000297 | ERS196662 |
| 2010_04_01_FLX3_Ti | 454FLX Ti | 750200 | 114726417 | 152.93 | 64.9 | 3kb | N | ERP000297 | ERS196664 |
| 2010_04_07_FLX3_Ti | 454FLX Ti | 718478 | 108541623 | 151.07 | 64.4 | 3kb | N | ERP000297 | ERS196664 |
| 2010_04_15_FLX3_Ti | 454FLX Ti | 1085060 | 165872052 | 152.9 | 63.7 | 3kb | N | ERP000297 | ERS196664 |
| 2010_05_25_FLX3_Ti | 454FLX Ti | 898745 | 137722297 | 153.2 | 64.5 | 3kb | N | ERP000297 | ERS196664 |
| 2010_08_06_FLX3_Ti | 454FLX Ti | 1,460,100 | 269,620,397 | 184.65 | 57.8 | 8kb | N | ERP000297 | ERS196665 |
| 2010_08_17_FLX3_Ti | 454FLX Ti | 1,136,776 | 188,273,043 | 165.62 | 44.4 | 8kb | N | ERP000297 | ERS196665 |
| 2010_08_18_FLX3_Ti | 454FLX Ti | 1,310,758 | 225,234,911 | 171.83 | 59.4 | 8kb | N | ERP000297 | ERS196665 |
| 2011_04_05_FLX1_Ti | 454FLX Ti | 1,076,742 | 170,584,888 | 158.43 | 42.4 | 20kb | N | ERP000297 | ERS196666 |
| 2011_04_14_FLX1_Ti | 454FLX Ti | 1,441,877 | 263,220,448 | 182.55 | 59.9 | 20kb | N | ERP000297 | ERS196666 |
| 3801_1 | Illumina GA2 | 30,810,664 | 2,341,610,464 | 76* | 100 | 250-350bp | N | ERP000297 | ERS002005 |
| 3801_2 | Illumina GA2 | 25,589,582 | 1,944,808,232 | 76* | 100 | 250-350bp | N | ERP000297 | ERS002006 |
| 4491_2 | Illumina GA2 | 37,605,766 | 4,061,422,728 | 108* | 100 | 250-350bp | N | ERP000297 | ERS002005 |
| 4491_3 | Illumina GA2 | 26853002 | 2,900,124,216 | 108* | 100 | 250-350bp | N | ERP000297 | ERS002006 |

**Table S2. Genome and gene model statistics for *Globodera pallida* compared to those for other published nematode genomes.** Values for *M. hapla* are from , and those for *B. xylophilus* from . Other statistics are derived from data available in Wormbase (release 221 for *M. incognita, C. elegans, P. pacificus* and *B. malayi*; release 235 for *A. suum* and *T. spiralis*). Completeness values are based on CEGs analysed with the CEGMA software package.

|  |  |  | ***Clade IV*** | | | ***Clade V*** | | ***Clade III*** | | ***Clade I*** |
| --- | --- | --- | --- | --- | --- | --- | --- | --- | --- | --- |
|  |  | *Globodera pallida* | *Bursaphelenchus xylophilus* | *Meloidogyne hapla* | *Meloidogyne incognita* | *C. elegans* | *Pristionchus pacificus* | *Brugia malayi* | *Ascaris suum* | *Trichinella spiralis* |
| **Genome statistics** | Estimated genome size (Mb) | 100 | 63-75 | 54 | 47-51 | 100 | Not available | 90-95 | 250 | 71 |
|  | Haploid chromosome # | 9 | 6 | 16 | Varies | 6 | 6 | 6 | 12 | 3 |
|  | Assembly length (Mb) | 124.7 | 74.6 | 53 | 86 | 100 | 172.5 | 95.8 | 272.8 | 64.3 |
|  | # Scaffolds | 6,873 | 1,231 | 1,523 | 2,817 | 7 | 18,083 | 8,180 | 1,618 | 8,794 |
|  | Scaffold N50 (kb) | 122 | 1,158 | 84 | 83 | 17,493 | 1,244 | 94 | 408 | 1,739 |
|  | Longest scaffold (kb) | 600 | 3,612 | 360 | 593 | 20,924 | 5,268 | 6,534 |  | 9,739 |
|  | GC content | 36.7 | 40.4 | 27.4 | 31.4 | 35.4 | 42 | 30.5 | 37.9 | 34 |
| **Gene model statistics** | Number of gene models | 16,419 | 18,074 | 14,420 | 19,212 | 20,056 | 23,500 | 18,348 | 18,542 | 15,808 |
|  | Gene density (genes / Mb) | 132 | 242 | 272 | 223 | 200 | 136 | 192 | 68 | 246 |
|  | Mean protein length (aa) | 361 | 345 | 310 | 354 | 440 | 332 | 312 | 327 | 317 |
|  | Mean/Median exon len. (bp) | 135 / 116 | 289 / 183 | 172 / 145 | 169 / 136 | 202 / 145 | 97 / 85 | 160 / 138 | 153 / 137 | 128 / 129 |
|  | Mean/median exons/gene | 8.01 / 6 | 4.5 /4 | 6.1 / 4 | 6.6 / 5 | 6.5 / 5 | 10.3 /8 | 5.9 / 3 | 6.4/5.0 | 5.78 / 4 |
|  | Mean/median intron len. (bp) | 190 / 91 | 153 / 69 | 154/55 | 230 /82 | 320/66 | 309/141 | 280 / 215 | 1023/690 | 198 / 83 |
| **Completeness** | CEGMA completeness  (% complete/partial) | 81/85 | 97/98 | 95/96 | 73/77 | 100/100 | 95/98 | 95/96 | 94/96 | 95/95 |
|  | CEG gene count (complete/partial) | 1.3/1.4 | 1.08/1.09 | 1.07/1.12 | 1.53/1.61 | 1.05/1.06 | 1.20/1.23 | 1.07/1.11 | 1.13/1.14 | 1.13/1.16 |

**Table S3. Summary of repeat families in the *Globodera pallida*** genome

| **Repeat type** | **Category** | **Families** | **No. copies** | **Coverage (bp)** | **% Genome** |
| --- | --- | --- | --- | --- | --- |
| LINE | LINE | 17 | 316 | 76,118 | 0.1% |
|  |  |  | (75) | (46,302) | (0.04%) |
| LTR retrotransposons | LTR | 218 | 3,015 | 726,087 | 0.6% |
|  |  |  | (513) | (450,225) | (0.4%) |
| TIR+Helitron+mu+mariner | DNA | 197 | 9,849 | 1,492,212 | 1.2% |
|  |  |  | (3,126) | (657,072) | (0.5%) |
| no TE feature |  | 880 | 147,164 | 19,390,648 | 15.6% |
|  |  |  | (64,894) | (11,071,848) | (8.9%) |
| Total |  | 1,312 | 160,344 | 21,685,065 | 17.4% |
|  |  |  | (68,608) | (12,225,447) | (9.8%) |

Values in parentheses correspond to the numbers with hits at least 50% length of consensus sequences.

**Table S4. Transcriptome (RNA-seq) sequencing libraries**

| **(internal) Library ID** | **ENA accession ID (sample)** | **Sequencing technology** | **Read length** | **Number of sequencing reads** | **Number of mapped reads** | **% reads mapped** | **% both paired maps** | **Average insert length** | **Life stage sampled** |
| --- | --- | --- | --- | --- | --- | --- | --- | --- | --- |
| 4912_1 | ERS091755 | Illumina GA2 | 76 | 52,227,148 | 24,976,944 | 47.8 | 60.8 | 974.1 | egg |
| 6566_6 | ERS092427 | Illumina GA2 | 76 | 48,731,544 | 30,372,935 | 62.3 | 79.4 | 585.2 | egg |
| 3251_5 | ERS001595 | Illumina GA2 | 76 | 25,827,170 | 11,397,345 | 44.1 | 79.8 | 453 | J2 |
| 5417_7 | ERS092081 | Illumina GA2 | 76 | 57,445,284 | 36,226,024 | 63.1 | 78.0 | 936 | J2 |
| 6566_5 | ERS092426 | Illumina GA2 | 76 | 55,324,762 | 35,873,697 | 64.8 | 79.3 | 651.4 | J2 |
| 6197_1 | ERS092348 | Illumina GA2 | 76 | 42,353,444 | 22,748,180 | 53.7 | 66.5 | 762.1 | 7 dpi |
| 6797_6#2 | ERS092525 | Illumina HiSeq | 100 | 105,328,064 | 58,995,842 | 56.0 | 75.2 | 802.8 | 7 dpi |
| 5145_2 | ERS091953 | Illumina GA2 | 76 | 67,062,672 | 39,785,840 | 59.3 | 75.5 | 925.6 | 14 dpi |
| 6985_8 | ERS092579 | Illumina HiSeq | 100 | 219,424,944 | 121,873,505 | 55.5 | 75.1 | 808.8 | 14 dpi |
| 3570_6 | ERS001598 | Illumina GA2 | 76 | 27,504,044 | 12,402,725 | 45.1 | 61.4 | 419.6 | 21 dpi |
| 6197_2 | ERS092349 | Illumina GA2 | 76 | 31,926,516 | 16,785,645 | 52.6 | 66.7 | 1119.9 | 21 dpi |
| 3251_3 | ERS001809 | Illumina GA2 | 76 | 27,685,290 | 12,394,391 | 44.8 | 76.6 | 344.6 | 28 dpi |
| 6197_3 | ERS092350 | Illumina GA2 | 76 | 40,236,262 | 21,950,290 | 54.6 | 67.2 | 1017.1 | 28 dpi |
| 3570_7 | ERS002001 | Illumina GA2 | 76 | 22,996,304 | 14,667,087 | 63.8 | 68.6 | 504.8 | 35 dpi |
| 6197_5 | ERS092351 | Illumina GA2 | 76 | 39,841,674 | 22,210,881 | 55.7 | 68.6 | 1009.8 | 35 dpi |
| 5145_1 | ERS091952 | Illumina GA2 | 76 | 67,462,472 | 40,925,685 | 60.7 | 75.8 | 1130.4 | Adult male |
| 6797_6#1 | ERS092525 | Illumina HiSeq | 100 | 95,704,886 | 53,807,634 | 56.2 | 74.6 | 1693.7 | Adult male |

**Table S5. Functional properties of *Globodera pallida*-restricted proteins.**

Shown are all GO terms significantly (p < 0.01) over-represented in annotations of *G. pallida* singleton proteins and proteins in *G. pallida*-specific gene families, based on top GO p-values shown in right-most column.

| Biological Process | | |
| --- | --- | --- |
| GO:0008152 | metabolic process | 5.90E-10 |
| GO:0009124 | nucleoside monophosphate biosynthetic process | 3.70E-06 |
| GO:0006508 | proteolysis | 1.10E-05 |
| GO:0006333 | chromatin assembly or disassembly | 3.10E-05 |
| GO:0006796 | phosphate metabolic process | 3.60E-05 |
| GO:0005991 | trehalose metabolic process | 7.70E-05 |
| GO:0015074 | DNA integration | 0.00012 |
| GO:0043170 | macromolecule metabolic process | 0.00037 |
| GO:0019226 | transmission of nerve impulse | 0.00211 |
| GO:0071702 | organic substance transport | 0.00215 |
| GO:0007592 | protein-based cuticle development | 0.00366 |
| GO:0017038 | protein import | 0.0069 |
| GO:0022008 | neurogenesis | 0.00735 |
| GO:0006952 | defense response | 0.00807 |
| GO:0044237 | cellular metabolic process | 0.00847 |
| GO:0000160 | two-component signal transduction system | 0.00974 |
| Molecular Function | | |
| GO:0003824 | catalytic activity | 6.00E-17 |
| GO:0016787 | hydrolase activity | 1.40E-09 |
| GO:0004190 | aspartic-type endopeptidase activity | 2.80E-07 |
| GO:0032559 | adenyl ribonucleotide binding | 3.20E-07 |
| GO:0005544 | calcium-dependent phospholipid binding | 3.80E-06 |
| GO:0004555 | alpha,alpha-trehalase activity | 1.40E-05 |
| GO:0003885 | D-arabinono-1,4-lactone oxidase activity | 7.30E-05 |
| GO:0003723 | RNA binding | 9.20E-05 |
| GO:0004672 | protein kinase activity | 1.00E-04 |
| GO:0003682 | chromatin binding | 0.00012 |
| GO:0016740 | transferase activity | 0.00093 |
| GO:0008408 | 3'-5' exonuclease activity | 0.00383 |
| GO:0003887 | DNA-directed DNA polymerase activity | 0.00433 |
| GO:0004252 | serine-type endopeptidase activity | 0.00433 |
| GO:0016829 | lyase activity | 0.00682 |
| GO:0016301 | kinase activity | 0.00742 |
| GO:0016772 | transferase activity | 0.00786 |
| GO:0000156 | two-component response regulator activity | 0.00858 |
| Cellular Component | | |
| GO:0000785 | chromatin | 7.10E-06 |
| GO:0031224 | intrinsic to membrane | 0.00037 |
| GO:0044421 | extracellular region part | 0.00445 |
| GO:0015630 | microtubule cytoskeleton | 0.00803 |
| GO:0044464 | cell part | 0.00882 |

**Table S6. RNA-seq evidence for diverse spliced leader sequences.** Counts of RNA-seq reads found with significant similarity to the spliced leader sequences previously reported . Columns show total numbers of reads hitting equal sequence, the number of reads hitting only a single SL sequence, and the number of reads hitting only SL sequences within a ‘subtype’ – indicated by the numerical part of the SL sequence name.

| **Spliced Leader sequence** | **total reads hit** | **reads uniquely hit** | **reads unique to subtype** |
| --- | --- | --- | --- |
| SL1 | 222437 | 11332 | 222437 |
| SL1a | 3512 | 463 | 3512 |
| SL1b | 231446 | 4157 | 231446 |
| SL1c | 2157 | 463.5 | 2157 |
| SL1d | 230560 | 1740 | 230560 |
| SL1e | 2697 | 731 | 2697 |
| SL1f | 217696 | 2147 | 217696 |
| SL1g | 3172 | 1279 | 3172 |
| SL1h | 244490 | 9454 | 244490 |
| SL1i | 3700 | 2251 | 3700 |
| Total reads hitting SL1-type sequences | | 289,531 | |
| SL2ag | 1105 | 5 | 1105 |
| SL2b | 300 | 174 | 300 |
| SL2c | 1021 | 41 | 1021 |
| SL2d | 307 | 137 | 307 |
| SL2e | 6724 | 5765 | 6724 |
| SL2f | 1009 | 35 | 1009 |
| SL2h | 207 | 75 | 207 |
| SL2i | 1031 | 51 | 1031 |
| Total reads hitting SL2-type sequences | | 7,579 | |
| SL3a | 597 | 243 | 597 |
| SL3b | 553 | 45 | 553 |
| SL3c | 7097 | 323 | 7097 |
| SL3d | 14571 | 5626 | 14571 |
| SL3e | 9416 | 472 | 9416 |
| SL3f | 245 | 111 | 245 |
| Total reads hitting SL3-type sequences | | 16,809 | |
| SL4a | 332 | 332 | 332 |
| SL4b | 0 | 0 | 0 |
| SL4c | 0 | 0 | 0 |
| SL4d | 0 | 0 | 0 |
| SL4e | 0 | 0 | 0 |
| SL4f | 108 | 108 | 108 |
| Total reads hitting SL4-type sequences | | 440 | |
| Total SL reads | | 314,359 | |

**Table S7. *Globodera pallida* effectors similar to effectors from other plant-parasitic nematodes (not including the SPRYSECS)**

| **Gene number** | **Putative function** |
| --- | --- |
| GPLIN_000591100 | *G. pallida* IVG9 effector |
| GPLIN_001541500 | Paralogue of IVG9 effector |
| GPLIN_000293500 | Paralogue of IVG9 effector |
| GPLIN_001098200 | Possible paralogue of IVG9 effector |
| GPLIN_001110200 | Possible paralogue of IVG9 effector |
| GPLIN_000638300 | *G. pallida* IA7 effector |
| GPLIN_000740500 | Paralogue of IA7 effector |
| GPLIN_000359000 | Similar to *G. rostochiensis* effector 1106 |
| GPLIN_000235400 | Similar to *G. rostochiensis* effector 1106 |
| GPLIN_000793000 | Similar to *G. rostochiensis* effector 1106 |
| GPLIN_000119200 | Similar to *G. rostochiensis* effector 1106 |
| GPLIN_000314000 | Similar to *G. rostochiensis* effector 1106 |
| GPLIN_000768400 | Similar to *G. rostochiensis* effector 1106 |
| GPLIN_000850500 | Similar to *G. rostochiensis* effector 1106 |
| GPLIN_001613000 | Similar to *G. rostochiensis* effector 1106 |
| GPLIN_000684200 | Similar to *G. rostochiensis* effector 1106 |
| GPLIN_001295300 | Similar to *G. rostochiensis* effector 1106 |
| GPLIN_000683800 | Similar to *G. rostochiensis* effector 1106 |
| GPLIN_001043600 | Similar to *G. rostochiensis* candidate effector |
| GPLIN_000812600 | Similar to *G. rostochiensis* candidate effector |
| GPLIN_000931100 | Similar to *G. rostochiensis* candidate effector |
| GPLIN_000376700 | Chorismate mutase effector |
| GPLIN_000666500 | Chorismate mutase effector |
| GPLIN_000594000 | Similar to *G. rostochiensis* C52 effector candidate |
| GPLIN_000697600 | Member of CLE effector protein family, 4 CLE repeats |
| GPLIN_001090600 | Member of CLE effector protein family, one CLE motif |
| GPLIN_001090500 | Member of CLE effector protein family |
| GPLIN_000950900 | Member of CLE effector protein family |
| GPLIN_000950800 | Member of CLE effector protein family, one CLE motif |
| GPLIN_000201400 | Similar to *G. rostochiensis* candidate effector E9 |
| GPLIN_000057600 | Similar to *G. rostochiensis* candidate effector E9 |
| GPLIN_000760900 | Similar to *G. rostochiensis* candidate effector E9 |
| GPLIN_000187800 | Similar to *G. rostochiensis* candidate effector E9 |
| GPLIN_000854400 | *G. pallida* orthologue of *H. glycines* G16H02 effector |
| GPLIN_000780600 | *G. pallida* orthologue of *H. glycines* effector G19C07 |
| GPLIN_001203000 | *G. pallida* orthologue of *H. glycines* effector 10C02 |
| GPLIN_000668700 | *G. pallida* orthologue of *H. glycines* effectors 25A01 and 30G12 |
| GPLIN_000015300 | *G. pallida* orthologue of *H. glycines* effector G7E05 |
| GPLIN_000167300 | Possible orthologue of *H glycines* G10A06 effector; similarity to E3 Ligases, secreted |
| GPLIN_000785400 | Possible orthologue of *H glycines* G10A06 effector; similarity to E3 Ligases, secreted |
| GPLIN_000393900 | Large protein includes sequence similar to *H glycines* effector scn1120. |
| GPLIN_001559100 | Similar to *H. glycines* secretory protein 11 putative effector. Similar to transthyretin-like proteins |
| GPLIN_000178900 | Similar to *H. glycines* secretory protein 11 putative effector. Similar to transthyretin-like proteins |
| GPLIN_000869800 | Similar to *H. glycines* secretory protein 11 putative effector. Similar to transthyretin-like proteins |
| GPLIN_000738800 | Similar to *H. glycines* secretory protein 11 putative effector. Similar to transthyretin-like proteins |
| GPLIN_000870000 | Similar to *H. glycines* secretory protein 11 putative effector. Similar to transthyretin-like proteins |
| GPLIN_000169700 | Similar to *H. glycines* secretory protein 12 putative effector. Similar to metalloprotease inhibitor |
| GPLIN_000621200 | Similar to *H. glycines* secretory protein 8 putative effector. |
| GPLIN_001317500 | Similar to *G. rostochiensis* candidate effector peptide |
| GPLIN_000901900 | Similar to *G. rostochiensis* candidate effector peptide |
| GPLIN_000901700 | Similar to *G. rostochiensis* candidate effector peptide |
| GPLIN_000325200 | Similar to *G. rostochiensis* candidate effector peptide |
| GPLIN_001199500 | Similar to *G. rostochiensis* candidate effector peptide |
| GPLIN_000207700 | Similar to *G. rostochiensis* candidate effector peptide |
| GPLIN_000442900 | Contains *G. pallida* orthologue of *H. glycines* G8A07 effector |
| Not annotated, present on scaffold 480 | Similar to *G. rostochiensis* A42 effector candidate family |
| Not annotated present on scaffold 50 | Similar to *G. rostochiensis* A42 effector candidate family |
| GPLIN_000604400 | Similar to *M. incognita* effector AY135365, J2 specific |
| GPLIN_000555600 | Similar to *M. incognita* effector AY135365, J2 specific |
| GPLIN_001416500 | Similar to *H. glycines* effector G19B10 |
| GPLIN_000370900 | Similar to *H. glycines* effector G19B10 |
| GPLIN_000996800 | Similar to *H. glycines* effector G12H04 |
| GPLIN_000926600 | Similar to *H. glycines* G20E03 effector |
| GPLIN_000962200 | Similar to *H. glycines* G20E03 effector |
| GPLIN_000662500 | Similar to *H. glycines* G20E03 effector |
| GPLIN_000977100 | Similar to *H. glycines* G20E03 effector |
| GPLIN_000668700 | Similar to *H. glycines* 30G12 effector |
| GPLIN_000638800 | Similar to *H. glycines* 30G12 effector |
| GPLIN_000637900 | Similar to *H. glycines* 30G12 effector |
| GPLIN_000668600 | Similar to *H. glycines* 30G12 effector |
| GPLIN_001339200 | Similar to *H. glycines* 30G12 effector |
| GPLIN_000120300 | Similar to *H. glycines* 30G12 effector |
| GPLIN_000667500 | Similar to *H. glycines* G4G05 and 30G12 effectors |
| GPLIN_000574800 | Similar to *H glycines* effector gland cell secretory protein 3. Contains thioredoxin-like domain |
| GPLIN_000990400 | Similar to *H glycines* effector gland cell secretory protein 3.  Contains thioredoxin-like domain |
| GPLIN_001205000 | Similar to *H glycines* effector gland cell secretory protein 3.  Contains thioredoxin-like domain |
| GPLIN_000248100 | Similar to *H. glycines* effector G16A01 |
| GPLIN_000933000 | Similar to *H. glycines* effector G17G01 |
| GPLIN_001526900 | Similar to *H. glycines* effector G17G01 |
| GPLIN_000297600 | Similar to *H. glycines* effector G17G01 |
| GPLIN_000167700 | GpUBI-EP effector similar to Ubiquitin extension proteins |
| GPLIN_000642100 | GpUBI-EP effector similar to Ubiquitin extension proteins |
| GPLIN_001038900 | Similar to *H. glycines* G18H08 effector |
| GPLIN_000060800 | Similar to *H. glycines* effectors 4D06 and G16B09 |
| GPLIN_001471200 | Similar to *H. glycines* effectors 4D06 and G16B09 |
| GPLIN_001038900 | Similar to *H. glycines* effectors 4D06 and G16B09 |
| GPLIN_000388900 | Similar to *H. glycines* effectors 4D06 and G16B09 |
| GPLIN_001255700 | Similar to *H. glycines* effectors 4D06 and G16B09 |
| GPLIN_000203300 | Similar to *H. glycines* effectors 4D06 and G16B09 |
| GPLIN_000481100 | Similar to *H. glycines* effectors 4D06 and G16B09 |
| GPLIN_000796500 | Similar to *H. glycines* effectors 4D06 and G16B09 |
| GPLIN_000912100 | Similar to *H. glycines* effectors 4D06 and G16B09 |
| GPLIN_000969800 | Similar to *H. glycines* effectors 4D06 and G16B09 |
| GPLIN_000970000 | Similar to *H. glycines* effectors 4D06 and G16B09 |
| GPLIN_001606400 | Similar to *H. glycines* effectors 4D06 and G16B09 |
| GPLIN_001221800 | Similar to *H. glycines* effectors 4D06 and G16B09 |
| GPLIN_001596100 | Similar to *H. glycines* effectors 4D06 and G16B09 |
| GPLIN_000950100 | Similar to *H. glycines* effectors 4D06 and G16B09 |
| GPLIN_000243800 | Similar to *H. glycines* effectors 4D06 and G16B09 |
| GPLIN_001390400 | Similar to *H. glycines* effectors 4D06 and G16B09 |
| GPLIN_000243700 | Similar to *H. glycines* effectors 4D06 and G16B09 |
| GPLIN_000950600 | Similar to *H. glycines* effectors 4D06 and G16B09 |
| GPLIN_001221900 | Similar to *H. glycines* effectors 4D06 and G16B09 |
| GPLIN_000860700 | Similar to *H. glycines* effectors 4D06 and G16B09 |
| GPLIN_001162100 | Similar to *H. glycines* effectors 4D06 and G16B09 |
| GPLIN_000970100 | Similar to *H. glycines* effectors 4D06 and G16B09 |
| GPLIN_001030900 | Similar to *H. glycines* effectors 4D06 and G16B09 |
| GPLIN_000803500 | Similar to *H. glycines* effectors 4D06 and G16B09 |
| GPLIN_000792900 | Similar to *H. glycines* effectors 4D06 and G16B09 |
| GPLIN_001337800 | Similar to *H. glycines* effectors 4D06 and G16B09 |
| GPLIN_001358800 | Similar to *H. glycines* effectors 4D06 and G16B09 |
| GPLIN_000969900 | Similar to *H. glycines* effectors 4D06 and G16B09 |
| GPLIN_000072400 | Similar to *H. glycines* effectors 4D06 and G16B09 |
| GPLIN_001456900 | Similar to *H. glycines* effectors 4D06 and G16B09 |
| GPLIN_000407400 | Similar to *H. glycines* effectors 4D06 and G16B09 |
| GPLIN_001431400 | Similar to *H. glycines* effectors 4D06 and G16B09 |
| GPLIN_001443600 | Similar to *H. glycines* effectors 4D06 and G16B09 |
| GPLIN_000126500 | Similar to *H. glycines* effectors 4D06 and G16B09 |
| GPLIN_000308900 | Similar to *H. glycines* effectors 4D06 and G16B09 |
| GPLIN_000309000 | Similar to *H. glycines* effectors 4D06 and G16B09 |
| GPLIN_001390500 | Similar to *H. glycines* effectors 4D06 and G16B09 |
| GPLIN_001582700 | Similar to *H. glycines* effectors 4D06 and G16B09 |
| GPLIN_001384700 | Putative effector similar to *H glycines* esophageal gland cell protein Hgg-20. |
| GPLIN_000349200 | Putative effector similar to *H. avenae* gland cell protein and *H. glycines* effector Hgg 20 |
| GPLIN_001475500 | Similar to RKN effector (gland cell protein 28). Similar to other nematode secreted proteins |
| GPLIN_000763000 | Similar to *H. glycines* effector G23G11 |
| GPLIN_000872800 | Similar to *H. glycines* effector 33A09 |
| GPLIN_000188200 | Putative effector similar to *H. avenae* gland cell protein |
| GPLIN_000107400 | Putative effector similar to *H. glycines* Hgg17 effector |
|  |  |

**Table S8. Cell wall modifying proteins in *Globodera pallida***

| **Gene number** | **Putative function** |
| --- | --- |
| GPLIN_000092400 | Putative expansin |
| GPLIN_000293400 | Putative expansin |
| GPLIN_000293700 | Putative expansin |
| GPLIN_000536200 | Putative expansin |
| GPLIN_000590900 | Putative expansin |
| GPLIN_000599100 | Putative expansin |
| GPLIN_000599200 | Putative expansin |
| GPLIN_001571600 | Putative expansin |
| GPLIN_001621500 | Putative expansin |
| GPLIN_000536400 | CBM2 domain |
| GPLIN_000616300 | CBM2 domain |
| GPLIN_000694900 | CBM2 domain |
| GPLIN_000706300 | CBM2 domain |
| GPLIN_000707900 | CBM2 domain |
| GPLIN_001031600 | CBM2 domain |
| GPLIN_000674600 | Putative GH43 Arabinase |
| GPLIN_000304900 | Putative GH5 cellulase (beta 1,4, endoglucanase) |
| GPLIN_000313600 | Putative GH5 cellulase (beta 1,4, endoglucanase) |
| GPLIN_000536400 | Putative GH5 cellulase (beta 1,4, endoglucanase) |
| GPLIN_000552400 | Putative GH5 cellulase (beta 1,4, endoglucanase) |
| GPLIN_000616300 | Putative GH5 cellulase (beta 1,4, endoglucanase) |
| GPLIN_000694900 | Putative GH5 cellulase (beta 1,4, endoglucanase) |
| GPLIN_000755100 | Putative GH5 cellulase (beta 1,4, endoglucanase) |
| GPLIN_000755200 | Putative GH5 cellulase (beta 1,4, endoglucanase) |
| GPLIN_000779000 | Putative GH5 cellulase (beta 1,4, endoglucanase) |
| GPLIN_000779200 | Putative GH5 cellulase (beta 1,4, endoglucanase) |
| GPLIN_000827200 | Putative GH5 cellulase (beta 1,4, endoglucanase) |
| GPLIN_001111200 | Putative GH5 cellulase (beta 1,4, endoglucanase) |
| GPLIN_001111300 | Putative GH5 cellulase (beta 1,4, endoglucanase) |
| GPLIN_001185800 | Putative GH5 cellulase (beta 1,4, endoglucanase) |
| GPLIN_001215600 | Putative GH5 cellulase (beta 1,4, endoglucanase) |
| GPLIN_001308700 | Putative GH5 cellulase (beta 1,4, endoglucanase) |
| GPLIN_000142900 | Putative GH53 arabinogalactan endo-1,4-beta-galactosidase |
| GPLIN_000143000 | Putative GH53 arabinogalactan endo-1,4-beta-galactosidase |
| GPLIN_000142600 | Putative PL3 Pectate lyase (similar to pectate lyase 2 family) |
| GPLIN_000294400 | Putative PL3 Pectate lyase (similar to pectate lyase 1 family) |
| GPLIN_000294500 | Putative PL3 Pectate lyase (similar to pectate lyase 1 family) |
| GPLIN_000322300 | Putative PL3 Pectate lyase (similar to pectate lyase 1 family) |
| GPLIN_000412300 | Putative PL3 Pectate lyase (similar to pectate lyase 2 family) |
| GPLIN_000467400 | Putative PL3 Pectate lyase (similar to pectate lyase 1 family) |
| GPLIN_000673000 | Putative PL3 Pectate lyase (similar to pectate lyase 1 family) |

**Table S9. *Globodera pallida* proteins containing a SPRY domain, including SPRYSECS.**

| GPLIN_000736500 | GPLIN_001465400 | GPLIN_001224300 | GPLIN_001225300 |
| --- | --- | --- | --- |
| GPLIN_000376500 | GPLIN_001310400 | GPLIN_001265900 | GPLIN_000507600 |
| GPLIN_000047700 | GPLIN_001058700 | GPLIN_000260100 | GPLIN_001378400 |
| GPLIN_001463100 | GPLIN_000426400 | GPLIN_000413700 | GPLIN_000800200 |
| GPLIN_000460700 | GPLIN_001363400 | GPLIN_000203800 | GPLIN_001327800 |
| GPLIN_000855400 | GPLIN_000822000 | GPLIN_000389800 | GPLIN_000242100 |
| GPLIN_001105100 | GPLIN_001465500 | GPLIN_001186200 | GPLIN_000158300 |
| GPLIN_000403000 | GPLIN_000632100 | GPLIN_000363400 | GPLIN_001135400 |
| GPLIN_000794700 | GPLIN_000312600 | GPLIN_001227400 | GPLIN_000800300 |
| GPLIN_000203700 | GPLIN_000057100 | GPLIN_000038300 | GPLIN_000385000 |
| GPLIN_000531200 | GPLIN_001246500 | GPLIN_001126000 | GPLIN_001598500 |
| GPLIN_000789100 | GPLIN_001253900 | GPLIN_001349800 | GPLIN_001185900 |
| GPLIN_000756600 | GPLIN_001048200 | GPLIN_000789300 | GPLIN_000867100 |
| GPLIN_000632600 | GPLIN_000984200 | GPLIN_000698900 | GPLIN_000312500 |
| GPLIN_001398800 | GPLIN_000716900 | GPLIN_001487300 | GPLIN_000260200 |
| GPLIN_001378200 | GPLIN_000043300 | GPLIN_000318800 | GPLIN_001206200 |
| GPLIN_000556700 | GPLIN_000200100 | GPLIN_001235900 | GPLIN_000530500 |
| GPLIN_001258400 | GPLIN_000627100 | GPLIN_000358100 | GPLIN_001440300 |
| GPLIN_000583000 | GPLIN_001096800 | GPLIN_000385300 | GPLIN_001418900 |
| GPLIN_000195600 | GPLIN_001166000 | GPLIN_001489200 | GPLIN_000659600 |
| GPLIN_001348800 | GPLIN_000909700 | GPLIN_001258100 | GPLIN_000467500 |
| GPLIN_001520400 | GPLIN_000259400 | GPLIN_001253800 | GPLIN_000880300 |
| GPLIN_001496800 | GPLIN_000908700 | GPLIN_000254600 | GPLIN_000157600 |
| GPLIN_001501200 | GPLIN_000312300 | GPLIN_001315800 | GPLIN_001352900 |
| GPLIN_001059500 | GPLIN_000531100 | GPLIN_001323300 | GPLIN_001004800 |
| GPLIN_001035300 | GPLIN_000381900 | GPLIN_000657200 | GPLIN_001566300 |
| GPLIN_000099300 | GPLIN_001000300 | GPLIN_001128900 | GPLIN_000639300 |
| GPLIN_001246900 | GPLIN_000530700 | GPLIN_000105400 | GPLIN_000038900 |
| GPLIN_000413600 | GPLIN_001178500 | GPLIN_000318600 | GPLIN_000008700 |
| GPLIN_001171400 | GPLIN_000632500 | GPLIN_000183900 | GPLIN_001009200 |
| GPLIN_000776300 | GPLIN_000200200 | GPLIN_001189400 | GPLIN_000426600 |
| GPLIN_000426700 | GPLIN_001260200 | GPLIN_000438000 | GPLIN_000312100 |
| GPLIN_000757500 | GPLIN_000046400 | GPLIN_000284600 | GPLIN_000893400 |
| GPLIN_000555800 | GPLIN_001327500 | GPLIN_000008900 | GPLIN_001332300 |
| GPLIN_001408700 | GPLIN_000583100 | GPLIN_000427100 | GPLIN_000427000 |
| GPLIN_000898200 | GPLIN_000930100 | GPLIN_001105500 | GPLIN_000437600 |
| GPLIN_000785600 | GPLIN_001310900 | GPLIN_001253600 | GPLIN_001253500 |
| GPLIN_000414100 | GPLIN_000196800 | GPLIN_001115700 | GPLIN_000626800 |
| GPLIN_000350100 | GPLIN_001586900 | GPLIN_000051600 | GPLIN_001060000 |
| GPLIN_000266800 | GPLIN_000259500 | GPLIN_000636900 | GPLIN_001378700 |
| GPLIN_000179400 | GPLIN_000659700 | GPLIN_000132500 | GPLIN_000636800 |
| GPLIN_000798500 | GPLIN_000047500 | GPLIN_001428700 | GPLIN_000626900 |
| GPLIN_000437400 | GPLIN_000450400 | GPLIN_001436900 | GPLIN_000787400 |
| GPLIN_000725400 | GPLIN_001362700 | GPLIN_000892800 | GPLIN_000892900 |
| GPLIN_001520200 | GPLIN_001099700 | GPLIN_001477200 | GPLIN_000177900 |
| GPLIN_000245400 | GPLIN_001168900 | GPLIN_000788900 | GPLIN_001150700 |
| GPLIN_001453900 | GPLIN_000292100 | GPLIN_001181800 | GPLIN_000971300 |
| GPLIN_001506200 | GPLIN_000756700 | GPLIN_001375400 | GPLIN_000238900 |
| GPLIN_000132400 | GPLIN_001310300 | GPLIN_001265800 | GPLIN_000862600 |
| GPLIN_001535200 | GPLIN_001131500 | GPLIN_001587400 | GPLIN_000008300 |
| GPLIN_001189000 | GPLIN_000414000 | GPLIN_000569300 | GPLIN_000382500 |
| GPLIN_001536900 | GPLIN_000696800 | GPLIN_000756400 | GPLIN_001253700 |
| GPLIN_001005900 | GPLIN_001522400 | GPLIN_000697500 | GPLIN_000509600 |
| GPLIN_000995700 | GPLIN_001173900 | GPLIN_001415300 | GPLIN_000755000 |
| GPLIN_000433800 | GPLIN_001488500 | GPLIN_000937900 | GPLIN_001022100 |
| GPLIN_000426000 | GPLIN_001446300 | GPLIN_001385900 | GPLIN_001271400 |
| GPLIN_000372100 | GPLIN_001035200 | GPLIN_001472400 | GPLIN_000803200 |
| GPLIN_000284700 | GPLIN_000099200 | GPLIN_000608300 | GPLIN_000632300 |
| GPLIN_000788000 | GPLIN_000905800 | GPLIN_001059100 | GPLIN_000152800 |
| GPLIN_000008400 | GPLIN_000074200 | GPLIN_000507800 | GPLIN_000133000 |
| GPLIN_001169300 | GPLIN_000320000 | GPLIN_001300800 | GPLIN_000082300 |
| GPLIN_000245500 | GPLIN_001083600 | GPLIN_000637000 | GPLIN_000252200 |
| GPLIN_000148800 | GPLIN_001480400 | GPLIN_000626700 | GPLIN_001032500 |
| GPLIN_001378600 | GPLIN_001424900 | GPLIN_000196200 | GPLIN_001171800 |
| GPLIN_000179900 | GPLIN_001212700 | GPLIN_001312600 | GPLIN_001082800 |
| GPLIN_001493900 | GPLIN_000620000 | GPLIN_000700300 | GPLIN_000802900 |
| GPLIN_000057000 | GPLIN_000390200 | GPLIN_001059400 | GPLIN_000299400 |
| GPLIN_001013600 | GPLIN_000294100 | GPLIN_000776500 | GPLIN_001059800 |
| GPLIN_000626500 | GPLIN_000725500 | GPLIN_001082900 | GPLIN_000756200 |
| GPLIN_000794500 | GPLIN_000843100 | GPLIN_000530300 | GPLIN_001223200 |
| GPLIN_000180800 | GPLIN_000094400 | GPLIN_000673400 | GPLIN_001060400 |
| GPLIN_000700500 | GPLIN_000531000 | GPLIN_001427300 | GPLIN_000369500 |
| GPLIN_001470700 | GPLIN_000531300 | GPLIN_001587100 | GPLIN_001551100 |
| GPLIN_000603900 | GPLIN_000328200 | GPLIN_001007400 | GPLIN_000495800 |
| GPLIN_000776700 | GPLIN_000800100 | GPLIN_001059900 |  |

**Table S10. Novel *Globodera pallida* secreted proteins up-**regulated in J2 or early parasitic stages that may represent novel effector candidates.

| GPLIN_000948600 | GPLIN_001463000 | GPLIN_000834600 |
| --- | --- | --- |
| GPLIN_001318000 | GPLIN_000847100 | GPLIN_000028200 |
| GPLIN_000319500 | GPLIN_000342300 | GPLIN_001232800 |
| GPLIN_001185000 | GPLIN_001263700 | GPLIN_000466900 |
| GPLIN_001268500 | GPLIN_000361100 | GPLIN_001391000 |
| GPLIN_000510600 | GPLIN_000744000 | GPLIN_000318900 |
| GPLIN_000957300 | GPLIN_000555400 | GPLIN_001008400 |
| GPLIN_001016900 | GPLIN_000208800 | GPLIN_001138500 |
| GPLIN_000927400 | GPLIN_000027900 | GPLIN_000142200 |
| GPLIN_000357600 | GPLIN_000886700 | GPLIN_000187400 |
| GPLIN_001262300 | GPLIN_000228700 | GPLIN_001335500 |
| GPLIN_000061100 | GPLIN_000063700 | GPLIN_000608100 |
| GPLIN_000713500 | GPLIN_001196900 | GPLIN_000897000 |
| GPLIN_000943100 | GPLIN_001153300 | GPLIN_000819000 |
| GPLIN_000172000 | GPLIN_000897600 | GPLIN_001127400 |
| GPLIN_000776900 | GPLIN_001004000 | GPLIN_000966000 |
| GPLIN_000126000 | GPLIN_001223000 | GPLIN_000886500 |
| GPLIN_000919700 | GPLIN_000609400 | GPLIN_000122100 |
| GPLIN_000723200 | GPLIN_000376600 | GPLIN_001080000 |
| GPLIN_000280900 | GPLIN_000281300 | GPLIN_000516100 |
| GPLIN_000495300 | GPLIN_000818900 | GPLIN_000271900 |
| GPLIN_000185800 | GPLIN_001244900 | GPLIN_000167000 |
| GPLIN_000424400 | GPLIN_000100500 | GPLIN_001030400 |
| GPLIN_001344300 | GPLIN_000886600 | GPLIN_000698800 |
| GPLIN_000283500 | GPLIN_000208700 | GPLIN_000195900 |
| GPLIN_001066900 | GPLIN_001099200 | GPLIN_001030700 |
| GPLIN_000120500 | GPLIN_000614900 | GPLIN_000589200 |
| GPLIN_001040900 | GPLIN_000641200 | GPLIN_001138300 |
| GPLIN_001031700 | GPLIN_000696300 | GPLIN_000689500 |
| GPLIN_001417900 | GPLIN_001184500 | GPLIN_000610000 |
| GPLIN_001319300 | GPLIN_000758500 | GPLIN_001304400 |
| GPLIN_000943000 | GPLIN_000187600 | GPLIN_001183800 |
| GPLIN_000333100 | GPLIN_000063100 | GPLIN_000241600 |
| GPLIN_000616800 | GPLIN_000319000 | GPLIN_001550200 |
| GPLIN_000333000 | GPLIN_000807000 | GPLIN_000140200 |
| GPLIN_001153200 | GPLIN_001138700 | GPLIN_000821100 |
| GPLIN_001592300 | GPLIN_000560800 | GPLIN_000258900 |
| GPLIN_001292400 | GPLIN_000758200 | GPLIN_001146800 |
| GPLIN_000075700 | GPLIN_000209100 | GPLIN_000925000 |

**Table S11. Comparison of putative detoxification genes identified in *Globodera pallida* with those found in *Meloidogyne incognita* and *Caenorhabditis elegans*.** Numbers of genes in each category are shown. Data for *C. elegans* and *M. incognita* are taken from . Only *C. elegans* gene families with a homolog in either *M. incognita* or *G. pallida* are shown.

| **Function** | **Gene family** | ***C. elegans*** | ***M. incognita*** | ***G. pallida*** |
| --- | --- | --- | --- | --- |
| Antioxidant | Catalase | 3 | 3 | 1 |
|  | Peroxiredoxin | 3 | 7 | 5 |
|  | Superoxide dismutase | 5 | 3 | 10 |
|  | Copper chaperonin | 1 | 2 | 2 |
|  | Glutathione peroxidase | 6 | 2 | 2 |
|  | Glutathione synthetase | 1 | 4 | 52 |
| Cytochrome P450 | CYP2 | 0 | 0 | 2 |
|  | CYP13 | 14 | 6 | 3 |
|  | CYP23 | 1 | 1 | 1 |
|  | CYP25 | 6 | 1 | 0 |
|  | CYP29 | 0 | 0 | 5 |
|  | CYP31 | 4 | 2 | 2 |
|  | CYP32 | 1 | 3 | 1 |
|  | CYP33 | 17 | 11 | 19 |
|  | CYP36 | 0 | 0 | 0 |
|  | CYP42 | 1 | 2 | 1 |
| Glutathione transferase | GST class sigma | 26 | 5 | 12 |
|  | GST class omega | 4 | 0 | 0 |
|  | GST class zeta | 2 | 0 | 0 |
|  | GST other classes | 12 | 0 | 1 |
| Glucuronosyl transferase | UGT | 64 | 38 | 34 |
| ABC transporter | ABC | 60 | 36 | 27 |

**Table S12. Presence of *C. elegans* immune response genes in *Globodera pallida* and other organisms.** Data for *M. incognita*, *C. briggsae*, *B. malayi*, *D. melanogaster* taken from .

| ***C. elegans*** | ***M. incognita*** | ***C. briggsae*** | ***B. malayi*** | ***D. melanogaster*** | ***G. pallida*** |
| --- | --- | --- | --- | --- | --- |
| **TGF-beta signalling pathway** |  |  |  |  |  |
| dbl-1 | Y | Y | Y | Y | Y |
| sma-2 | Y | Y | Y | Y | Y |
| sma-3 | Y | Y | Y | Y | Y |
| sma-4 | Y | Y | Y | Y | Y |
|  |  |  |  |  |  |
| **ERK MAPK signalling pathway** |  |  |  |  |  |
| lin-45 | Y | Y | Y | Y | Y |
| mak-2 | Y | Y | Y | Y | Y |
| mpk-1 | Y | Y | Y | Y | Y |
|  |  |  |  |  |  |
| **P39 MAPK signalling pathway** |  |  |  |  |  |
| nsy-1 | Y | Y | Y | Y | Y |
| pmk-1 | Y | Y | Y | Y | Y |
| sek-1 | Y | Y | Y | Y | Y |
| tir-1 | Y | Y | Y | Y | Y |
|  |  |  |  |  |  |
| **Toll signalling pathway** |  |  |  |  |  |
| tol-1 | Y | Y |  | Y | Y |
| trf-1 |  | Y |  | Y |  |
| lkb-1 | Y | Y | Y |  |  |
| plk-1 | Y | Y | Y | Y | Y |

**Table S13. Comparison of nuclear hormone receptors identified in *Globodera pallida* with those found in other organisms.** Data from *C. elegans* are from . Data and nomenclature from *B. malayi* are from . Data for *M. incognita* are from , with only receptors for which there are clear orthology relationships with other known receptors indicated in the table. Groups which are unrepresented in nematode species are excluded from the table.

| Group | *C. elegans* | *B. malayi* | *M. incognita* | *G. pallida* |
| --- | --- | --- | --- | --- |
| 0A | odr-7 | BmNHR-B |  | GPLIN_000471400 |
| 1D | nhr-85 | BmNHR11 |  | GPLIN_000228400 |
| 1E + G | sex-1  CNRD |  |  | GPLIN_000153900 |
| 1F | (HR3)  NHR-23 | BmNHR13 | Minc10028 Minc03383 | GPLIN_001187300  GPLIN_001482800  GPLIN_000052400  GPLIN_000052600 |
| 1H |  | BmNHR3 |  |  |
| 1J + K | DAF-12 NHR-8 NHR-48 | BmNHR17 BmNHR31 | Minc18589 Minc13296 | GPLIN_001266500  GPLIN_000678700 |
| 2A | supNRs | supNRs | supNRs |  |
| 2B |  | BmNHR4 |  |  |
| 2D | NHR-41 | BmNHR5 |  | GPLIN_001122300  GPLIN_001105600  GPLIN_000098300 |
| 2E | NHR-67 FAX-1 | BmNHR15 BmNHR16 | Minc12751 Minc02801 | GPLIN_000079400  GPLIN_000669800 |
| 2F | unc-55 | BmNHR25 |  |  |
| 4A | (CNR8)  NHR-6 |  |  | GPLIN_001187300 |
| 5A | NHR-25 | BmNHR14 |  | GPLIN_001106000  GPLIN_000548600 |
| 6A | NHR-91 | BmNHR21 |  | GPLIN_000099400 |
| SupNR | NHR-1  NHR-3  NHR-5  NHR-7  NHR-14  NHR-17  NHR-19  NHR-31  NHR-32  NHR-33  NHR-35  NHR-40  NHR-47  NHR-49  NHR-61  NHR-64  NHR-66  NHR-70  NHR-71  NHR-80  NHR-88  NHR-91  NHR-97  NHR-101  NHR-105  NHR-107  NHR-109  NHR-138  NHR-168  NHR-173  NHR-205  NHR-236  NHR-258  NHR-277 | BmNHR22  BmNHR10  BmNHR24  BmNHR18  BmNHR19 | Minc15185  Minc01725  Minc11307  Minc11538  Minc17538  Minc02316  Minc02318  Minc15420  Minc11986  Minc01325  Minc16419  Minc15059 | GPLIN_000337500  GPLIN_001003100  GPLIN_000669000  GPLIN_000279200  GPLIN_001447800  GPLIN_000327200  GPLIN_000616500  GPLIN_000219800  GPLIN_000268400  GPLIN_001534100  GPLIN_000628500  GPLIN_000805100  GPLIN_001410700  GPLIN_000989800  GPLIN_000612800  GPLIN_000765100  GPLIN_001175700  GPLIN_000284100  GPLIN_000297000  GPLIN_001175800  GPLIN_001410900  GPLIN_001629700  GPLIN_000663400  GPLIN_000607300  GPLIN_000168900  GPLIN_000452800  GPLIN_001543200  GPLIN_000686000  GPLIN_000890900  GPLIN_001590100  GPLIN_001203600  GPLIN_000456600  GPLIN_000694800  GPLIN_001127800  GPLIN_000282900  GPLIN_000097300  GPLIN_000196500 |
| Total | 14 + 270 supNRs | 13 + 5 supNRs | 6 + 12 supNRs | 18 + 36 supNRs |

**Table S14. *Globodera pallida* orthologs and genes with high similarity to *Caenorhabditis elegans* genes related to diapause*.*** In bold are represented Reciprocal Best Hits using >=40% identities and >=70% coverage; in normal letters are represented genes with >=30% identities and >=50% coverage; -: genes which do not fulfill these requirements. Bit score in brackets.

| ***C. elegans* Pathway** | **Protein** | ***G. pallida*** |
| --- | --- | --- |
| **Guanylyl cyclase pathway** |  |  |
| **DAF-11** | Transmembrane guanylate cyclase | GPLIN_000580700 (628); GPLIN_001400600 (584) |
| **TAX-2** | cGMP-gated channel | **GPLIN_000270000 (720)** |
| **TAX-4** | cGMP-gated channel | GPLIN_000399000 (692) |
| **TGF*β*-like** |  |  |
| **DAF-1** | TGF-β type I receptor | - |
| **DAF-3** | SMAD transcription factor | - |
| **DAF-4** | TGF*β* type II receptor | GPLIN_001316400 (218) |
| **DAF-5** | Proline rich protein | - |
| **DAF-7** | BMP/TGF-β | - |
| **DAF-8** | SMAD transcription factor | - |
| **DAF-14** | SMAD transcription factor | GPLIN_001484500 (96) |
| **SCD-1** | Glutamine rich protein | - |
| **SCD-2** | Tyrosine kinase | - |
| **BRA-1** | Zn-finger protein | - |
| **KIN-8** | Tyrosine kinase | - |
| **EGL-4** | cGMP-dependent protein kinase | - |
| **Insulin/IGF** |  |  |
| **DAF-2** | Insulin receptor | - |
| **DAF-15** | Ortholog RAPTOR protein | GPLIN_000644600 (498) |
| **DAF-16** | FOXO transcription factor | - |
| **DAF-18** | Phosphoinositide 3-phosphatase PTEN | - |
| **DAF-28** | β-insulin | - |
| **AGE-1** | Phosphoinositide 3-kinase | - |
| **PDK-1** | 3-phophoinositide-dependent kinase | **GPLIN_000703300 (417)** |
| **AKT-1** | Serine/threonine kinase | GPLIN_000475200 (404) |
| **AKT-2** | Serine/threonine kinase | GPLIN_000475200 (378) |
| **SGK-1** | Serine/threonine kinase | GPLIN_000373700(267) |
| **Steroid hormone pathway** |  |  |
| **DAF-9** | Cytochrome P450 | - |
| **DAF-12** | Nuclear receptor | - |
| **DAF-36** | Rieske oxygenase, hormone pathway | - |
| **Other processes** |  |  |
| **DAF-6** | amphid morphology | GPLIN_000159500 (733) |
| **DAF-10** | WD-WAA rep | GPLIN_001144000 (937) |
| **DAF-19** | RFX transcription factor | GPLIN_000191300 (225) |
| **DAF-21** | HSP-90 | **GPLIN_000887800 (1083)** |

**Table S15. Presence of *C. elegans* RNAi pathway genes in *Globodera pallida* and other nematodes.** Data for other nematodes taken from and .

| ***C. elegans*** | ***B. xylophilus*** | ***A. suum*** | ***B. malayi*** | ***M. hapla*** | ***M. incognita*** | ***G. pallida*** |
| --- | --- | --- | --- | --- | --- | --- |
| **Small RNA biosynthetic proteins** |  |  |  |  |  |  |
| drh-3 | Y | Y | Y | Y | Y | Y |
| drsh-1 | Y | Y | Y | Y | Y | Y |
| xpo-1 | Y | Y | Y | Y | Y | Y |
| xpo-2 | Y | Y | Y | Y | Y | Y |
| dcr-1 | Y | Y | Y |  | Y | Y |
| drh-1 | Y | Y | Y | Y | Y |  |
| pash-1 | Y |  | Y | Y | Y | Y |
| rde-4 | Y |  | Y |  |  |  |
| xpo-3 |  | Y | Y |  |  |  |
|  |  |  |  |  |  |  |
| **dsRNA uptake and spreading** |  |  |  |  |  |  |
| **Amplification** |  |  |  |  |  |  |
| smg-2 | Y | Y | Y | Y | Y | Y |
| smg-6 | Y | Y | Y | Y | Y | Y |
| ego-1 | Y | Y | Y | Y | Y | Y |
| rrf-3 | Y | Y | Y |  |  |  |
| rrf-1 | Y | Y |  |  |  |  |
| smg-5 |  |  |  |  |  |  |
| rsd-2 |  |  |  |  |  |  |
| **Spreading** |  |  |  |  |  |  |
| rsd-3 |  | Y | Y | Y | Y | Y |
| sid-1 |  |  |  |  |  |  |
| rsd-6 | Y |  |  |  |  |  |
| sid-2 |  |  |  |  |  |  |
|  |  |  |  |  |  |  |
| **Argonautes** |  |  |  |  |  |  |
| alg-1 | Y | Y | Y | Y | Y | Y |
| R06C7.1 | Y | Y | Y | Y | Y | Y |
| C04F12.1 |  | Y | Y | Y | Y |  |
| F58G1.1 |  | Y | Y | Y | Y | Y |
| alg-4 |  | Y |  |  | Y |  |
| rde-1 | Y |  |  |  |  |  |
| C16C10.3 |  |  |  |  |  | Y |
| ppw-1 |  |  |  |  |  |  |
| csr-1 | Y |  |  |  |  |  |
| ppw-2 |  |  |  |  |  |  |
| sago-1 |  |  |  |  |  |  |
| T22B3.2 | Y |  |  |  |  |  |
| T22H9.3 |  |  |  |  | Y | Y |
| alg-2 | Y | Y |  | Y | Y |  |
| ergo-1 |  |  |  |  |  |  |
| prg-1 |  |  |  |  |  |  |
| F55A12.1 |  | Y |  |  |  | Y |
| T23D8.7 | Y |  |  |  |  |  |
| nrde-3 |  |  |  |  |  |  |
| sago-2 |  |  |  |  |  |  |
| T23B3.2 |  |  |  |  |  |  |
| Y49F6A.1 | Y |  |  |  |  | Y |
| ZK1248.7 | Y | Y |  |  |  | Y |
| prg-2 |  |  |  |  |  |  |
|  |  |  |  |  |  |  |
| **Other RISC components** |  |  |  |  |  |  |
| tsn-1 | Y | Y | Y | Y | Y | Y |
| ain-1 | Y | Y | Y | Y | Y |  |
| vig-1 | Y | Y | Y |  |  |  |
| ain-2 |  |  | Y |  |  |  |
|  |  |  |  |  |  |  |
| **RNAi inhibitors** |  |  |  |  |  |  |
| eri-1 |  | Y | Y | Y | Y | Y |
| xrn-2 | Y | Y | Y | Y | Y | Y |
| adr-2 | Y | Y |  | Y |  |  |
| xrn-1 |  | Y | Y |  |  |  |
| adr-1 |  | Y | Y |  |  |  |
| lin-15b |  |  |  |  |  |  |
| eri-5 |  |  |  |  |  |  |
| eri-6/7 |  |  |  |  |  |  |
| eri-3 |  |  |  |  |  |  |
|  |  |  |  |  |  |  |
| **Nuclear RNAi effectors** |  |  |  |  |  |  |
| mut-7 | Y | Y | Y | Y |  |  |
| cid-1 | Y | Y | Y | Y | Y | Y |
| ekl-1 | Y |  | Y | Y | Y |  |
| gfl-1 | Y | Y | Y |  | Y | Y |
| mes-2 | Y | Y | Y |  | Y | Y |
| ekl-4 | Y | Y | Y | Y | Y | Y |
| mes-6 | Y | Y | Y | Y |  |  |
| rha-1 | Y | Y | Y | Y | Y | Y |
| ekl-6 | Y |  | Y |  |  |  |
| zfp-1 |  | Y | Y | Y |  |  |
| mut-2 |  |  |  |  |  |  |
| ekl-5 |  |  |  |  |  |  |
| mes-3 |  |  |  |  |  |  |
| mut-16 |  |  |  |  |  |  |
| rde-2 |  |  |  |  |  |  |

**Table S16. Comparison of neurotransmitter receptor families between *Caenorhabditis elegans* and *Globodera pallida***. The number of genes present representing each receptor type is indicated.

| **Receptor type** | ***C. elegans* genes** | ***G. pallida* genes** |
| --- | --- | --- |
| **Acetylcholine** | | |
| ACR-16 type nAChR | 11 | 4 |
| UNC-38 type nAChR | 3 | 3 |
| UNC-29 type nAChR | 4 | 4 |
| DEG-3 type nAChR | 8 | 7 |
| ACR-8 type nAChR | 3 | 3 |
|  | | |
| **Serotonin** | | |
| GPCR | 4 | 2 |
| Ligand-gated ion channel | 1 | 1 |
|  | | |
| **Dopamine** | | |
| GPCR | 5 | 4 |
|  | | |
| **Tyramine** | | |
| GPCR | 2 | 2 |
|  | | |
| **Octopamine** | | |
| GPCR | 2 | 2 |
|  | | |
| **Glutamate** | | |
| glutamate-gated chloride channel | 6 | 4 |
| ionotropic glutamate receptor | 11 | 9 |
| metabotropic glutamate receptor | 3 | 4 |
|  | | |
| **GABA** | | |
| GABA-anion channel receptor | 2 | 2 |
| metabotropic GABA receptor | 2 | 3 |

**Table S17. Presence of neurotransmitter biosynthesis, transport and metabolism genes in *Globodera pallida.*** Yes indicates presence of a clear reciprocal ortholog of the *C. elegans* gene; No indicates the absence of a clear ortholog.

| ***G. pallida* ortholog** | **Gene function** | ***C. elegans* gene** |
| --- | --- | --- |
| **Acetylcholine** | | |
| Yes | choline acetyltransferase | *cha-1* |
| Yes | synaptic acetylcholine transporter | *unc-17* |
| Yes | choline transporter | *cho-1* |
| Yes | post-synaptic transporter | *snf-6* |
| Yes | acetylcholinesterase | *ace-1* |
| Yes | acetylcholinesterase | *ace-2* |
| Yes | acetylcholinesterase | *ace-3* |
| No | acetylcholinesterase | *ace-4* |
|  | | |
| **Serotonin** | | |
| Yes | tryptophan hydroxylase | *tph-1* |
| Yes | GTP-cyclohydrolase I | *cat-4* |
| Yes | aromatic AA decarboxylase | *bas-1* |
| Yes | vesicular monoamine transporter | *cat-1* |
| Yes | serotonin reuptake transporter | *mod-5* |
| No | monoamine oxidase | *amx-1* |
| No | monoamine oxidase | *amx-2* |
| No | monoamine oxidase | *amx-3* |
|  | | |
| **Dopamine** | | |
| No | tyrosine hydroxylase | *cat-2* |
| Yes | dopamine reuptake transporter | *dat-1* |
|  | | |
| **Tyramine** | | |
| Yes | tyrosine decarboxylase | *tdc-1* |
|  | | |
| **Octopamine** | | |
| Yes | tyramine β-hydroxylase | *tbh-1* |
|  | | |
| **Glutamate** | | |
| Yes | vesicular glutamate transporter | *eat-4* |
| Yes | plasma membrane glutamate transporter | *glt-1* |
|  | | |
| **GABA** | | |
| Yes | glutamate decarboxylase | *unc-25* |
| Yes | vesicular GABA transporter | *unc-47* |
| Yes | GABA transporter | *snf-11* |
| Yes | GABA transaminase | *gta-1* |

**Table S18. Presence of *flp* neuropeptide-encoding genes in *G. pallida* and comparison with *M. incognita* and *B. xylophilus*.** Data for *M. incognita* taken from and for *B. xylophilus* from .

| ***flp* gene** | ***G. pallida*** | ***M. incognita*** | ***B. xylophilus*** |
| --- | --- | --- | --- |
| 1 | Yes – cDNA clone | yes | yes |
| 2 |  |  | yes |
| 3 | yes | yes | yes |
| 4 |  |  | yes |
| 5 | yes | yes | yes |
| 6 | yes - 2 copies | yes | yes |
| 7 | Yes - EST | yes | yes |
| 8 |  |  | yes |
| 9 |  |  |  |
| 10 |  |  |  |
| 11 | yes - more than one gene |  | yes |
| 12 | yes | yes | yes |
| 13 | yes | yes | yes |
| 14 | yes | yes | yes |
| 15 |  |  |  |
| 16 | yes - 2 different genes | yes | yes |
| 17 |  |  | yes |
| 18 | yes | yes | yes |
| 19 | yes | yes | yes |
| 20 |  | yes | yes |
| 21 | yes | yes | yes |
| 22 |  | yes | yes |
| 23 |  |  |  |
| 24 |  |  |  |
| 25 | yes | yes |  |
| 26 |  |  |  |
| 27 | yes | yes |  |
| 28 |  |  |  |
| 29 |  |  |  |
| 30 | yes | yes |  |
| 31 |  | yes |  |
| 32 | yes | yes | yes |
| 33 |  |  | yes |

**Table S19. Presence of *nlp* neuropeptide-encoding genes in *Globodera pallida* and comparison with *Meloidogyne incognita* and *Bursaphelenchus xylophilus***. Data for *M. incognita* taken from and for *B. xylophilus* from .

| ***nlp* gene** | ***G. pallida* gene** | ***M. incognita*** | ***B. xylophilus*** |
| --- | --- | --- | --- |
| 1 | GPLIN_000148300 | yes |  |
| 2 |  | yes | yes |
| 3 | GPLIN_000306500 | yes | yes |
| 4 |  |  |  |
| 5 |  |  |  |
| 6 |  | yes |  |
| 7 |  |  | yes |
| 8 | GPLIN_000702900 | yes | yes |
| 9 |  | yes | yes |
| 10 | GPLIN_000270800 | yes | yes |
| 11 |  |  |  |
| 12 | GPLIN_001153700 | yes | yes |
| 13 |  | yes | yes |
| 14 |  | yes | yes |
| 15 | GPLIN_000384700 | yes | yes |
| 16 |  |  |  |
| 17 |  | yes |  |
| 18 |  | yes |  |
| 19 |  |  |  |
| 20 |  |  | yes |
| 21 | GPLIN_000942800 | yes |  |
| 22 |  | yes |  |
| 23 |  |  |  |
| 34 |  |  |  |
| 35 |  |  |  |
| 36 |  | yes |  |
| 37 |  | yes | yes |
| 38 | GPLIN_001127600 | yes | yes |
| 39 |  |  |  |
| 40 | GPLIN_001156000 | yes | yes |
| 41 |  |  |  |
| 42 | GPLIN_000071400 | yes | yes |
| 43 |  |  |  |
| 44 |  | yes |  |
| 45 |  |  |  |
| 46 |  |  |  |
| 47 |  |  |  |

**Supporting References**
